# An X‑Shaped Chiral Non‑Fullerene n‐Type Semiconductor: Application in Perovskite Solar Cells and Chirality‑Induced Spin Selectivity

**DOI:** 10.1002/anie.9296585

**Published:** 2026-08-26

**Authors:** Yuki Sakamoto, Koki Otsuka, Kuraudo Ishihara, Shuang Li, Ai Shimazaki, Megumi Haramatsu, Junichiro Hirano, Juntaro Nogami, Hiroshi Shinokubo, Hiroki Uratani, Hironori Kaji, Fumitaka Ishiwari, Akinori Saeki, Tomoya Nakamura, Atsushi Wakamiya, Norihito Fukui

**Affiliations:** ^1^ Department of Molecular and Macromolecular Chemistry Graduate School of Engineering, and Integrated Research Consortium on Chemical Science (IRCCS) Nagoya University Nagoya Aichi Japan; ^2^ Institute for Chemical Research Kyoto University Uji Kyoto Japan; ^3^ Department of Applied Chemistry Graduate School of Engineering The University of Osaka Suita Osaka Japan; ^4^ Department of Molecular Engineering Graduate School of Engineering Kyoto University Kyoto Japan; ^5^ Department of Applied Chemistry for Environment Graduate School of Urban Environmental Sciences Tokyo Metropolitan University Hachioji Tokyo Japan; ^6^ HIKARI‐COOL Kyoto, Kyoto University Institute for Advanced Study Yoshida Ushinomiya‐cho Kyoto Japan

**Keywords:** CBBC, chirality‐induced spin‐selectivity, non‐fullerene acceptor, n‐type semiconductor, perovskite solar cell

## Abstract

The development of a new building block for creating solution‐processable amorphous n‐type semiconductors provides a platform of fundamental importance for advanced organic electronic materials. We have recently demonstrated that oxidative inner‑bond cleavage of readily available dibenzo[*g*,*p*]chrysene affords a chiral figure‑eight macrocycle that bears electron‑withdrawing carbonyl groups, namely cyclobisbiphenylenecarbonyl (CBBC). In this study, we report a highly electron‑accepting derivative of CBBC (CBBC‑TIC), in which the CBBC core is connected to four electron‑withdrawing dicyanomethylene‑3‑indanone (IC) units via thiophene spacers. The distinctive X‑shaped molecular structure of CBBC‑TIC enables the solution‐process fabrication of a homogeneous amorphous film with good electron conductivity. Perovskite solar cells that contain CBBC‑TIC as the electron‐transport layer achieved a power conversion efficiency of 20.6%, which is comparable to that obtained with the benchmark fullerene derivative [6,6]‐phenyl‐C_61_‐butyric acid methyl ester (PCBM). Furthermore, the enantiomers of CBBC‐TIC exhibited spin‐selective electron transport arising from the chirality‐induced spin‐selectivity (CISS) effect, with remarkably high enantiospecific magnetic conductance asymmetry values of up to 80% in amorphous films. These findings demonstrate that CBBC represents a versatile molecular component for the creation of advanced non‑fullerene n‐type semiconductors.

## Graphical abstract

A highly electron‑accepting derivative of cyclobisbiphenylenecarbonyl (CBBC‐TIC) is reported. CBBC‐TIC functions as an electron‐transport layer for perovskite solar cells, achieving a power conversion efficiency of 20.6%. Furthermore, the enantiomers of CBBC‐TIC exhibited spin‐selective electron transport with high enantiospecific magnetic conductance asymmetry values of up to 80%.

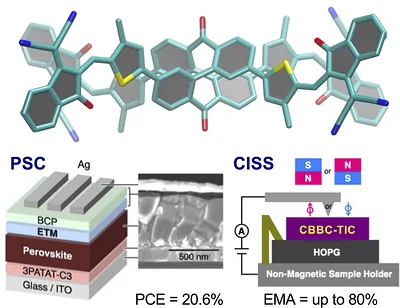

## 1 Introduction

Organic n‐type semiconductors are essential components in organic electronic devices, including organic light‐emitting diodes (OLEDs), organic field‐effect transistors (OFETs), organic photovoltaics (OPVs), and perovskite solar cells (PSCs) [1, 2, 3, 4, 5, 6, 7, 8]. Of the various small‐molecule n‐type semiconductors used in OPVs and PSCs, fullerene derivatives such as [6, 6]‐phenyl‐C_61_‐butyric acid methyl ester (PCBM) have long served as a benchmark material (Figure 1a) [9, 10, 11]. The advantage of these fullerene derivatives is the spherical structure that enables high electron mobility via isotropic electron‐transport. However, fullerenes suffer from inherent drawbacks, which include high production costs, limited tunability of their energy levels, and insufficient light‐harvesting ability.

**FIGURE 1:**
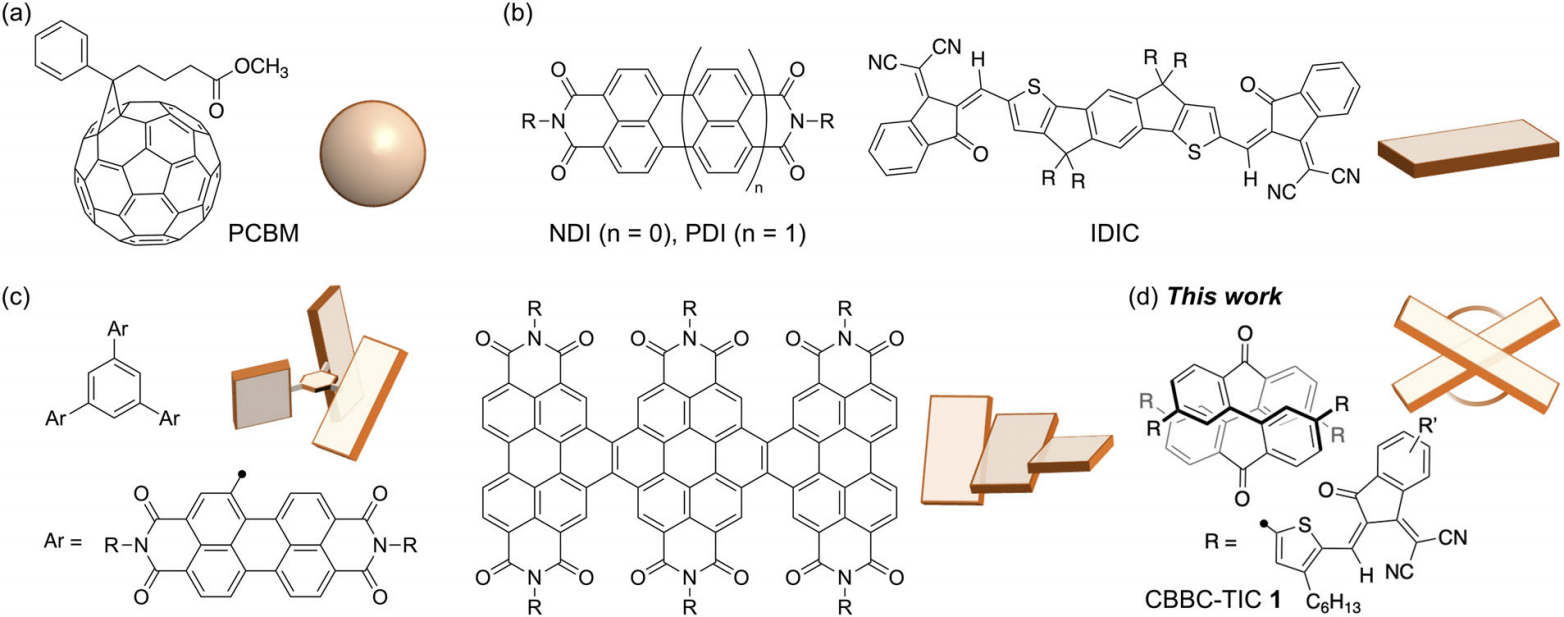
Small‐molecule electron‐transporting materials with distinct molecular geometries.

To overcome these limitations, non‐fullerene acceptors (NFAs) have been developed over the last two decades [12, 13, 14, 15]. Representative NFAs include rylene diimides such as naphthalene diimide (NDI) and perylene diimide (PDI) [16, 17, 18, 19, 20, 21, 22], as well as acceptor–donor–acceptor (A–D–A)‐type systems such as indacenodithiophene bis(dicyanomethylene‐3‐indanone) (IDIC) (Figure 1b) [23, 24, 25, 26, 27]. These molecules typically adopt flat, plate‐like structures, as is conventional for π‐conjugated systems. In contrast, the incorporation of structural nonplanarity into the design of NFAs has recently emerged as a promising design strategy as nonplanar architectures can suppress excessive aggregation and facilitate more isotropic electron transport in the solid state due to their three‐dimensional geometries. Reported nonplanar NFAs can be broadly subdivided into two categories: (1) propeller‐shaped systems, wherein multiple PDI units are connected to a central π‐core via single bonds [28, 29, 30, 31], and (2) helically twisted systems, in which PDI units are fused in a manner that induces steric repulsion (Figure 1c) [32, 33, 34].

Meanwhile, demand has increased for chiral organic n‐type semiconductors due to their various attractive functions, which include chiroptical sensing and chirality‐induced spin selectivity (CISS) [35, 36, 37, 38, 39]. Recently, several chiral single‐molecule p‐type semiconductors have shown high enantiospecific magnetic conductance asymmetry (EMA) values (up to ∼ 80%) [40, 41, 42], which are comparable to those reported for highly ordered assemblies such as supramolecular polymers (50%–85%) [43, 44] and inorganic two‐dimensional materials (90%) [45]. In contrast, the development of n‐type organic semiconductors that exhibit similarly high EMA values remains challenging because the current molecular design strategies for chiral NFAs predominantly rely on peripheral modifications of otherwise achiral π‐conjugated cores.

Previously, we have developed a concise and scalable synthesis of a nonplanar π‐acceptor via a skeletal‐transformation approach (Figure 2). Specifically, oxidative inner‐bond cleavage of readily available dibenzo[*g*,*p*]chrysene (DBC) furnished cyclobisbiphenylenecarbonyl (CBBC) on the gram scale [46, 47, 48]. CBBC can be regarded as a cyclic benzophenone dimer with electron‐withdrawing carbonyl groups embedded in a macrocyclic conjugation. Moreover, CBBC adopts a highly symmetric nonplanar chiral structure with *D*_2_ symmetry, which has encouraged the exploration of its various distinctive functions [49, 50, 51, 52]. These structural and electronic features prompted us to explore CBBC as a building block for NFAs.

**FIGURE 2:**
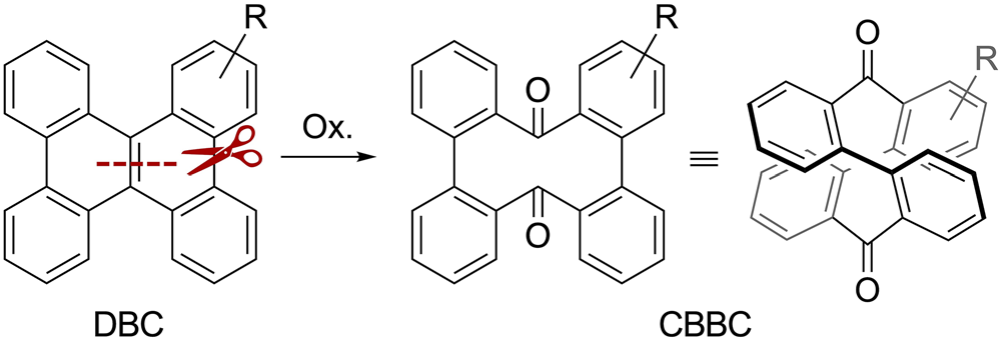
Concise synthesis of CBBC via oxidative inner‐bond cleavage.

Here, we report the synthesis of a CBBC‐TIC **1**, that is, a CBBC‐based nonplanar NFA in which the CBBC core is connected to four electron‐withdrawing dicyanomethylene‐3‐indanone (IC) units via thiophene spacers (Figure 1d). The X‐shaped geometry of CBBC‐TIC **1** is reminiscent of that of spirobifluorene derivatives such as Spiro‐OMeTAD [53, 54], a benchmark hole‐transporting material (HTM) for PSCs [27]. We evaluated the potential of CBBC‐TIC **1** as an electron‐transporting material (ETM) in inverted (p‐i‐n) PSCs, which are particularly attractive due to their low‐temperature processability and compatibility with flexible and tandem solar cells [8, 55, 56, 57, 58, 59, 60, 61, 62, 63, 64, 65, 66, 67]. We also evaluated the spin‐filter function of CBBC‐TIC **1** using spin‐polarized magnetic conductive atomic force microscopy (mc‐AFM).

## 2 Results and Discussion

### 2.1 Synthesis

Three CBBC‐TIC derivatives, **1a–1c**, were synthesized with varying numbers of methyl substituents on the terminal benzene rings (Scheme 1). 3,6,11,14‐Tetrabromo CBBC **2** was synthesized via oxidative inner‐bond cleavage of the corresponding brominated DBC, as previously reported [50]. The Miyaura borylation of brominated CBBC **2** with bis(pinacolato)diboron (B_2_pin_2_) afforded borylated CBBC **3** in 91% yield. The boryl groups of **3** were converted to 4‐formyl‐3‐hexylthienyl groups via a Suzuki–Miyaura cross‐coupling reaction with 5‑bromo‑3‑hexylthiophene‑2‑carbaldehyde (**4**) to afford **5** in 58% yield. The condensation of **5** with 1‐(dicyanomethylene)‐3‐indanone (**6a**) in CHCl_3_/pyridine furnished CBBC‐TIC **1a** in 81% yield. The terminal tetra‐ and octa‐methyl derivatives **1b** and **1c** were synthesized from the corresponding mono‐ and di‐methyl indanone derivatives **6b** and **6c**, respectively. The synthesis cost for **1a** was roughly estimated based on the materials used (Figure S27 and Table S5), indicating that CBBC‐TIC **1a** can be produced without any high‐cost steps. We also synthesized benzophenone derivative BP‐TIC **8** as a reference half unit of CBBC‐TIC **1a** from borylated benzophenone **9**.

**SCHEME 1:**
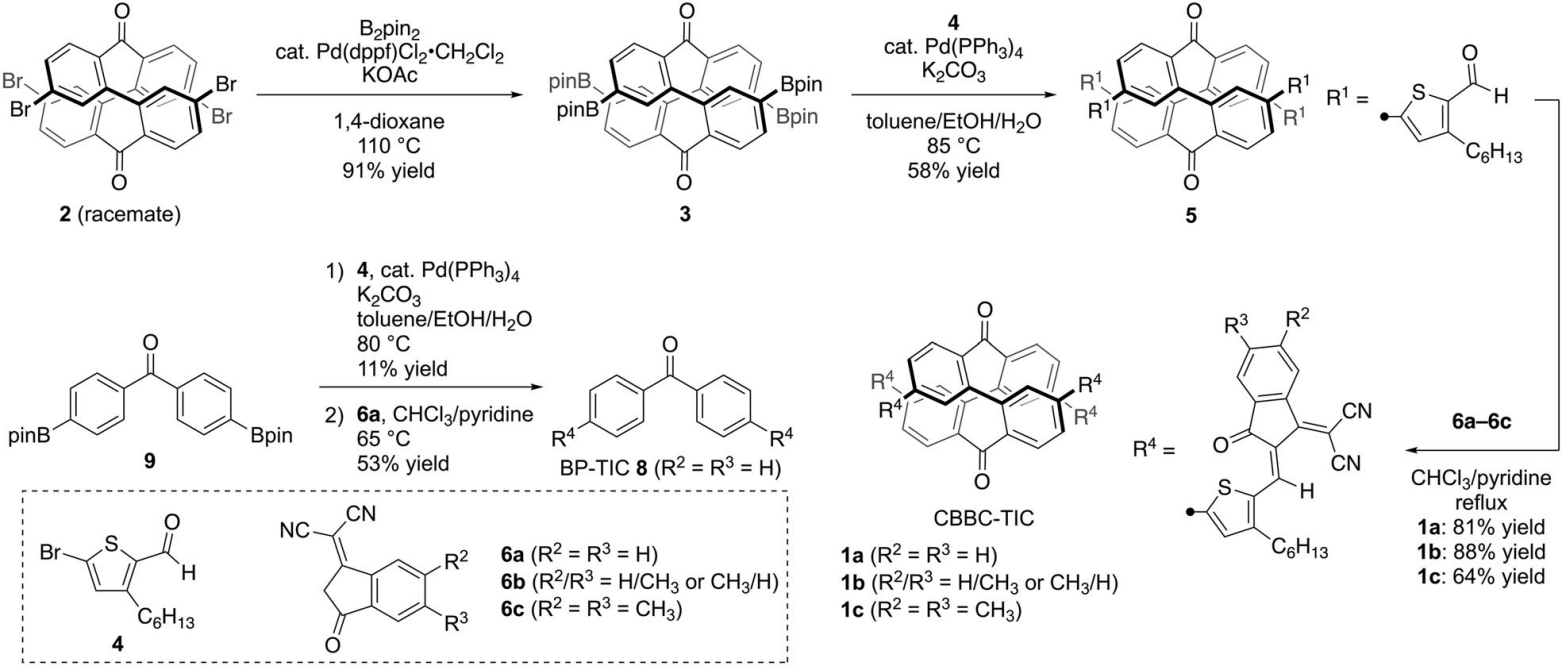
Synthesis of CBBC‐TICs **1a–1c** and BP‐TIC **8**.

At room temperature, CBBC‐TIC **1a** is virtually insoluble in common organic solvents such as CH_2_Cl_2_, toluene, hexane, THF, DMF, or MeOH. However, in boiling 1,1,2,2‐tetrachloroethane (TCE), CBBC‐TIC **1a** dissolves effectively with a high solubility value of 15 mg mL^−1^. Importantly, precipitation was not observed after the solution was cooled to room temperature, enabling a solution‐based process for device fabrication (*vide infra*). BP‐TIC **8** exhibited better solubility than CBBC‐TIC **1a**, as it was moderately soluble in CH_2_Cl_2_. Methyl‐substituted CBBC‐TICs **1b** and **1c** also exhibited improved solubility compared to **1a**, presumably due to the interrupted stacking in the solid states.

CBBC is chiral, and its enantiomers are configurationally stable [45]. Thus, we prepared the enantiomers of CBBC‐TIC **1a**. The low solubility of **1a** hampered its resolution using preparative chiral high‐performance liquid chromatography (HPLC). Hence, precursor **5** was subjected to chiral HPLC using DAICEL CHIRALPAK IA‐3 as the chiral stationary phase (eluent: CH_2_Cl_2_/hexane = 1/1). Then, the condensation of the separated enantiomers with **6** furnished the enantiomers of CBBC‐TIC **1a** without loss of enantiomeric excess. The absolute configuration of the enantiomers of **1a** was determined via time‐dependent density‐functional‐theory (TD‐DFT) calculations. The racemization barrier of CBBC‐TIC **1a** was determined by heating one enantiomer at 443 K in TCE and monitoring the decrease of the CD signal (Figure S23). The obtained half‐life of 12 h is higher than that of the non‐substituted CBBC (1.5 h).

### 2.2 Electronic Properties in Solution

The UV/vis absorption spectra of **1a**, **1b**, **1c**, **5**, and **8** in TCE are shown in Figure 3a. CBBC‐TIC **1a** exhibits a broad absorption band at 535 nm with a high extinction coefficient (1.5 × 10^5^ cm^−1^ M^−1^) that tails to approximately 640 nm. The good light‐harvesting ability of **1a** is advantageous for its application in OPVs. The absorption of **1a** is much more red‐shifted than that of its precursor **5** and that of non‐substituted CBBC, indicating the presence of effective conjugation over the condensed IC units. The spectral shape of **1a** resembles that of BP‐TIC **8**, suggesting that the molecules are electronically similar. The absorption spectra of methyl‐substituted CBBC‐TICs **1b** and **1c** are also essentially similar to that of **1a**, showing slight blue shifts with increasing methyl substitution. An emission from CBBC‐TIC **1a** was not detected, neither in CH_2_Cl_2_ nor in a polymethyl methacrylate (PMMA) film. The circular dichroism (CD) spectra of the enantiomers of CBBC‐TIC **1a** are shown in Figure 3b. These spectra are mirror images of each other. Owing to the extended π‐conjugation of CBBC‐TIC **1a**, the CD spectra cover the entire visible region, tailing to approximately 600 nm with moderate dissymmetry factors (*g*) of up to 0.6 × 10^−3^ (Figure S24).

**FIGURE 3:**
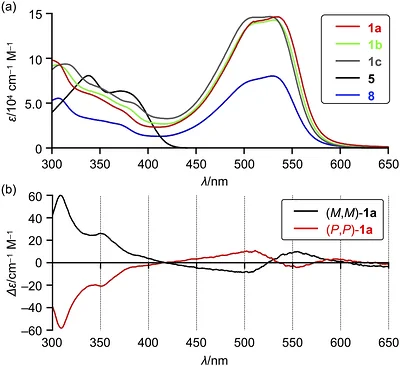
(a) UV/vis absorption spectra of **1a**, **1b**, **1c**, **5**, and **8**. (b) CD spectra of the enantiomers of **1a**. Solvent: TCE; *λ*: wavelength; *ε*: extinction coefficient.

The cyclic voltammograms of CBBC‐TIC **1a** and BP‐TIC **8** were measured in CH_2_Cl_2_/TCE using 0.1 M Bu_4_NPF_6_ as the supporting electrolyte and Ag/AgNO_3_ as the reference electrode (Figure S22). The ferrocene/ferrocenium couple (Fc/Fc^+^) was used as an external reference. CBBC‐TIC **1a** exhibits one irreversible reduction wave at −1.05 V. The reduction potential of BP‐TIC **8** is identical to that of **1a**, suggesting its suitability as a reference compound. These reduction potentials are comparable to that of PCBM [68].

The electronic properties of **1a** were further evaluated via DFT calculations at the B3LYP/6‐31G(d) level using model compound **1a'**, in which the hexyl groups are replaced with methyl groups to reduce calculation costs. The LUMO of **1a'** is effectively delocalized over the entire π‐system (Figure 4), which is preferable for an efficient electron transport in the solid state. Similar calculations for **1b'** and **1c'** indicate that methyl substitution on the IC units slightly raises the LUMO energy level compared with **1a'**, by 0.08 and 0.14 eV, respectively. This result is consistent with the experimentally observed blue shifts in the absorption spectra, highlighting the potential of the CBBC‐TIC framework for fine‐tuning of electron‐accepting ability via peripheral structural modification. According to the TD‐DFT calculations the intense absorption at 535 nm should be assigned to the allowed S_0–_S_1_ transition with an oscillator strength of 1.09. Consequently, the ratio between the transition electronic and magnetic dipole moments | ***μ*** |/| ***m*** | (3.2 × 10^3^) is much higher than 1, while the arrangement of the moments is parallel due to the *D*_2_‐symmetric structure, thus resulting in a small *g* value of 1.2 × 10^−3^.

**FIGURE 4:**
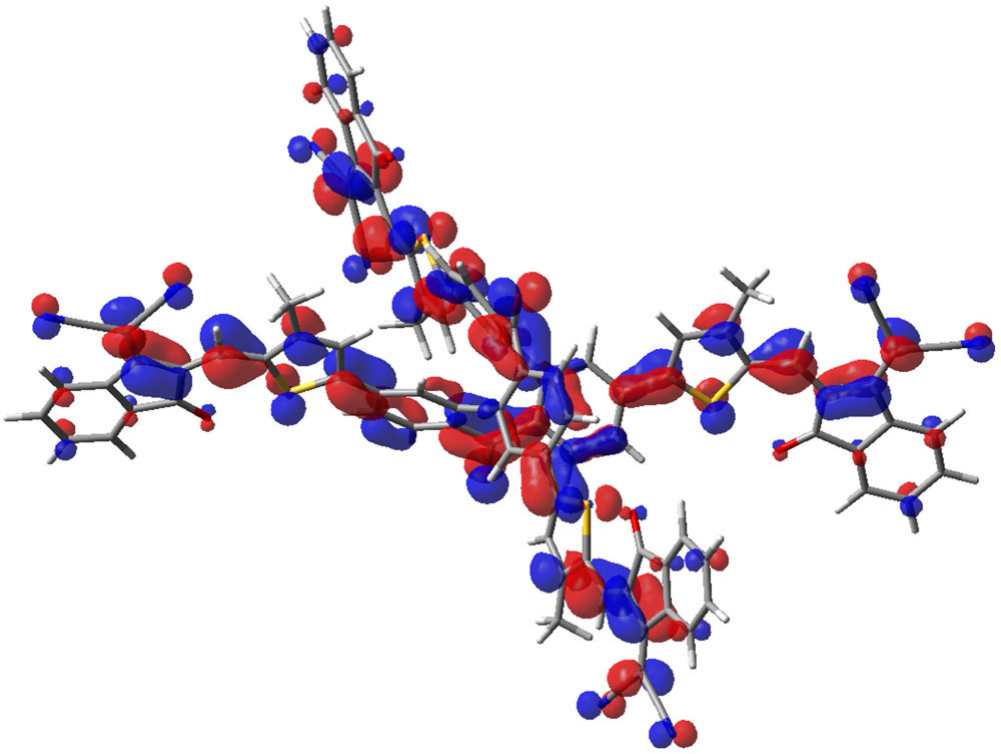
LUMO of **1a'** calculated at the B3LYP/6‐31G(d) level (isovalue = 0.02).

### 2.3 Thin‐Film Properties

Thin films were fabricated on glass or Si substrates by spin‐coating solutions of CBBC‐TIC **1a** and BP‐TIC **8** in TCE (15 mg mL^−1^). Ellipsometry measurements determined the film thicknesses of CBBC‐TIC **1a** and BP‐TIC **8** to be 35.7 ± 0.2 nm and 31.7 ± 0.1 nm, respectively. The UV/vis absorption spectra of **1a** and **8** in the film state show an absorption band at ∼530 nm, similar to the corresponding solution spectra (Figure S29a). The optical band gaps of **1a** and **8** were estimated to be 2.08 eV using Tauc plots (Figure S29b). The crystallinity and surface morphology of the thin films were investigated using two‐dimensional grazing‐incidence x‐ray diffraction (2D‐GIXD) and AFM. The 2D‐GIXD patterns of both materials exhibited no distinct Bragg diffraction peaks, indicating predominantly amorphous character (Figure 5a). The AFM image of the BP‐TIC **8** film showed needle‐like aggregates at the surface with a root‐mean‐square (RMS) roughness of 7.23 nm (Figure 5b). In contrast, the CBBC‐TIC **1a** films displayed a smooth surface with a significantly lower RMS roughness of 0.27 nm. The homogeneous morphology of the CBBC‐TIC **1a** films highlights the advantage of the nonplanar CBBC core, which is able to suppress molecular aggregation during film formation.

**FIGURE 5:**
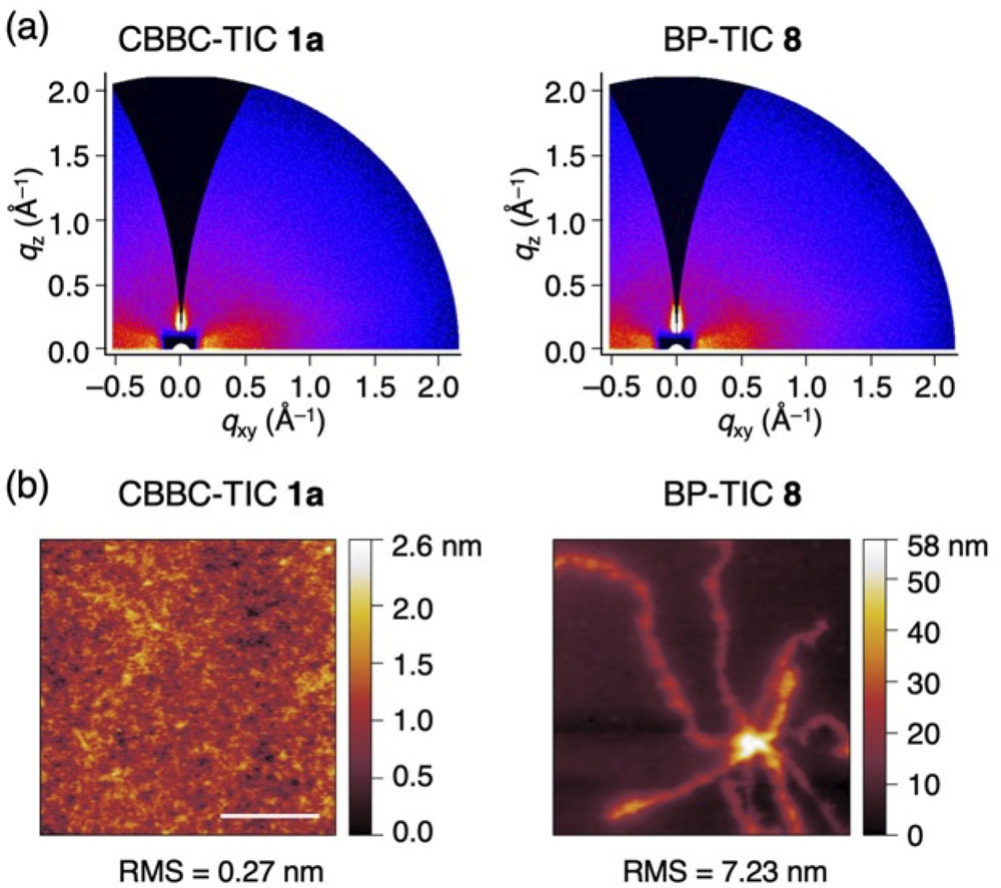
(a) Two‐dimensional grazing‐incidence x‐ray diffraction (2D‐GIXD) patterns and (b) AFM images for thin films of CBBC‐TIC **1a** and BP‐TIC **8** fabricated on Si substrates.

Space‐charge limited current (SCLC) measurements were carried out in order to evaluate the electron mobility (*µ*_e_). Electron‐only devices were fabricated with an indium tin oxide (ITO)/tin oxide (SnO_2_)/ETM/bathocuproine (BCP)/Ag structure (Figure S30a). The current‐density/voltage (*J–V*) characteristics were analyzed using the Mott–Gurney law (*J* = (9/8) *εε*_0_ *µ*_e_ *V* ^2^L^−3^), where *ε*_0_ is the permittivity of free space (8.85 × 10^−12^ F m^−1^), *ε* is the relative permittivity of the material (∼3 for organic semiconductors), and *L* is the film thickness (Figure S30b). The estimated electron mobilities were 3.9 ×10^−5^ cm^2^ V^−1^s^−1^ for CBBC‐TIC **1a** and 2.0 ×10^−5^ cm^2^ V^−1^s^−1^ for BP‐TIC **8**.

To clarify the origin of the higher electron mobility of CBBC‐TIC **1a** compared to that of BP‐TIC **8**, we simulated electron‐transport processes via a multiscale simulation [69, 70]. Amorphous‐aggregate structures of racemic CBBC‐TIC **1a** and BP‐TIC **8** were constructed using molecular dynamics (MD) simulations. The reorganization energies, intermolecular electronic couplings, and LUMO energy gaps for molecular pairs in the aggregate structures were evaluated using quantum chemical calculations. Electron‐transport processes were then simulated using the kinetic Monte–Carlo method based on intermolecular electron‐hopping‐rate constants estimated according to the Marcus formula, using the calculated reorganization energies, electronic couplings, and energy gaps. Further methodological details are provided in Supporting Information.

Figure 6 shows the resulting electron mobilities as a function of the square root of the applied electric field (*F* ^1/2^), indicating that CBBC‐TIC **1a** exhibits higher mobility than BP‐TIC **8**. The calculated mobilities of CBBC‐TIC **1a** and BP‐TIC **8** fall in the range of 2.1 ×10^−6^–2.4 × 10^−5^ and 2.4 × 10^−7^–1.2 × 10^−5^ cm^2^ V^−1^s^−1^, respectively. These simulated mobilities are in reasonable agreement with the experimental values. It should be noted here that in general, the measured electron mobilities depend strongly on the measurement technique and that our multiscale simulations mimic time‐of‐flight measurements, which differ from SCLC. As the amorphous‐aggregate structures used in our simulations are macroscopically homogeneous and free from defects and boundaries, the results suggest that the superior electron mobilities in CBBC‐TIC **1a** originate from the bulk properties, rather than from grain boundaries. A possible origin of the better bulk electron transport observed in CBBC‐TIC **1a** than in BP‐TIC **8** is enhanced electronic coupling. CBBC‐TIC **1a** is a dimer consisting of two BP‐TIC subunits, in which the electronic coupling between the two components is significant due to their close proximity and well‐defined orientation. Conversely, in the amorphous aggregate of BP‐TIC **8**, the intermolecular distance and orientation are essentially random.

**FIGURE 6:**
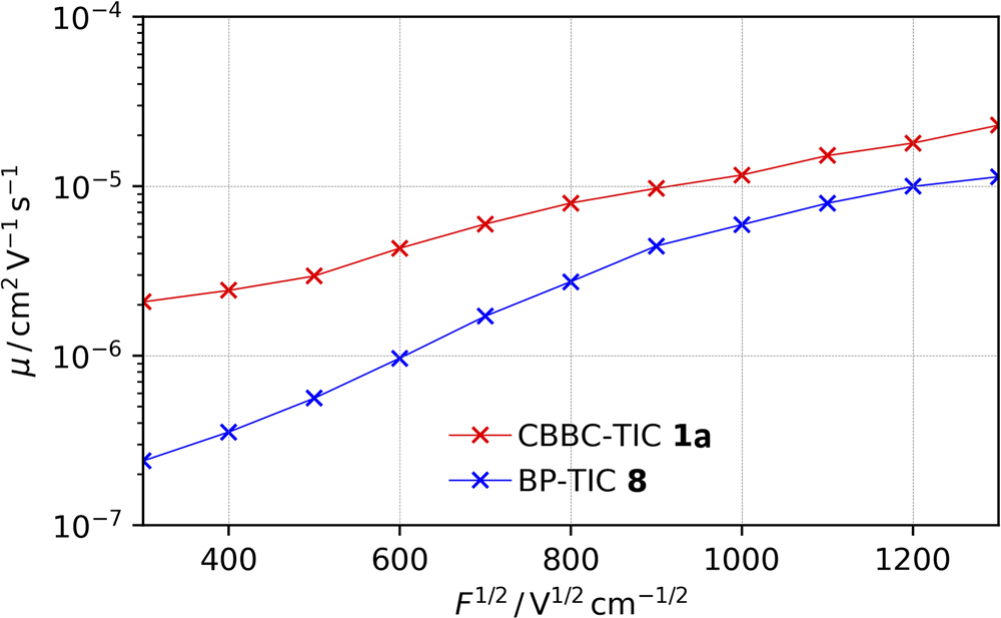
Simulated electron mobilities (*µ*_e_) of racemic CBBC‐TIC **1a** (red) and BP‐TIC **8** (blue) as a function of the square root of the applied electric field (*F* ^1/2^).

### 2.4 NFA Characteristics in PSCs

To assess the potential of the CBBC‐based NFAs as ETMs, p‐i‐n type PSCs were fabricated with a glass/ITO/3PATAT‐C3/Cs_0.05_FA_0.80_MA_0.15_PbI_2.75_Br_0.25_ (FA: formamidinium, MA: methylammonium)/ethylenediammonium diiodide (EDAI_2_)/ETMs /bathocuproine (BCP)/Ag device configuration (Figure 7a). A triazatruxene‐derived tripodal molecule, 3PATAT‐C3 [58], was used as the hole‐collecting monolayer, while EDAI_2_ was applied as a post‐treatment to passivate the perovskite surface and to enhance the open‐circuit voltage [71, 72, 73].

**FIGURE 7:**
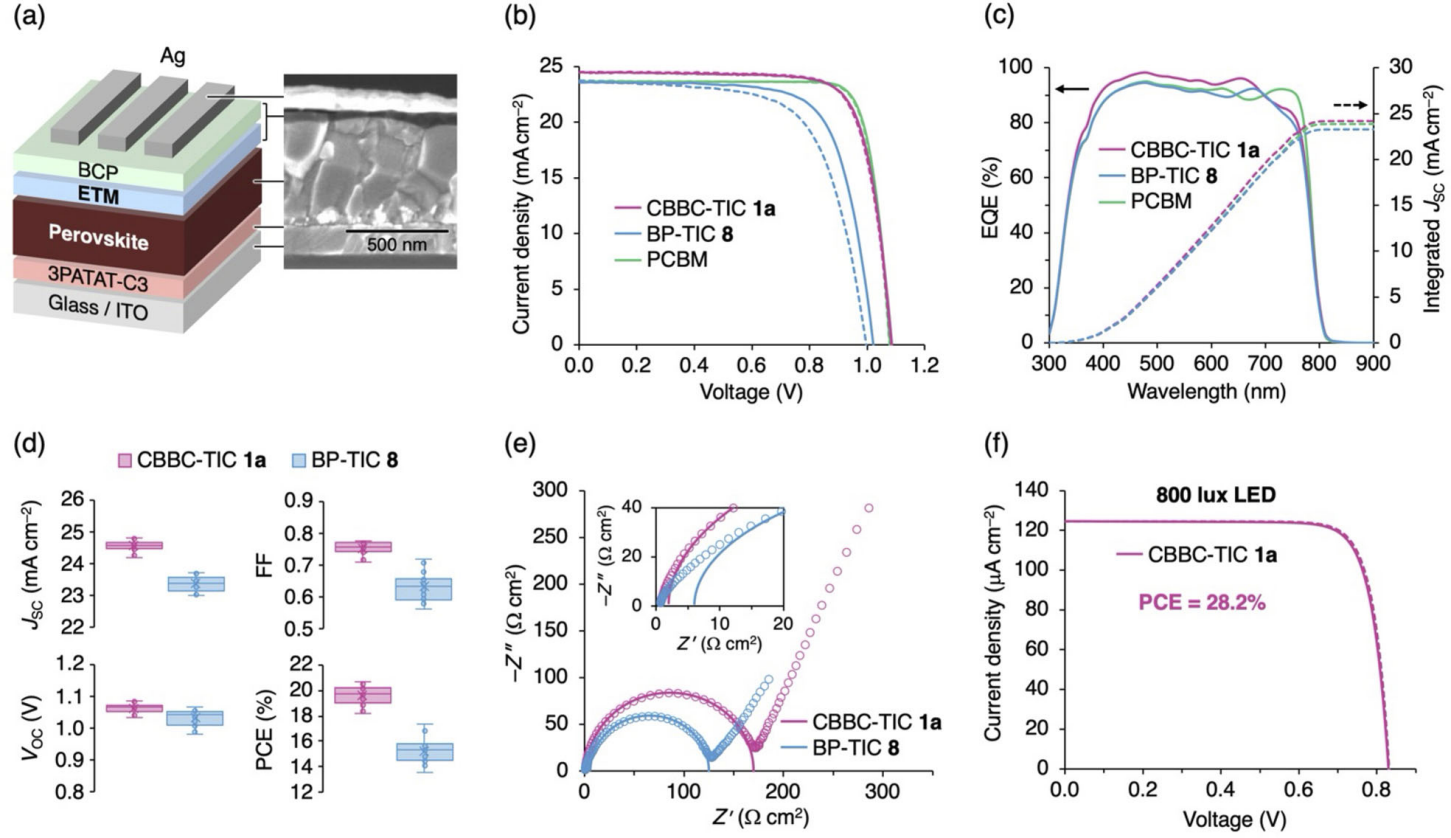
(a) Device structure and cross‐sectional SEM image, (b) current–voltage (*J–V*) curves of forward (*J*_SC_ to *V*_OC_, dotted lines) and reverse (*V*_OC_ to *J*_SC_, solid lines) scans, and (c) external quantum efficiency (EQE) spectra of the perovskite solar cells (PSCs). (d) Statistical distribution of photovoltaic parameters obtained from nine cells measured in both forward and reverse scan directions, giving 18 datasets in total. (e) Electrical impedance spectra under 1‐sun illumination. (f) *J–V* curves under a low light intensity of 800 lux using a white LED.

The current‐density/voltage (*J–V*) curves of the devices are presented in Figure 7b, and the corresponding device parameters are summarized in Table 1. The device using BP‐TIC **8** showed a power conversion efficiency (PCE) of 17.3%, with a short‐circuit current density (*J*_SC_) of 23.6 mA cm^−2^, an open‐circuit voltage (*V*_OC_) of 1.02 V, and a fill factor (FF) of 0.72 (reverse scan). Meanwhile, devices using CBBC‐TIC **1a** exhibited an enhanced *J*_SC_ of 24.5 mA cm^−2^, a *V*_OC_ of 1.09 V, and an FF of 0.78, resulting in a PCE of 20.6%. This efficiency is comparable to that of PCBM‐based devices (*J*_SC_ = 23.7 mA cm^−2^, *V*_OC_ = 1.08 V, FF = 0.83, PCE = 21.2%). The CBBC‐TIC‐based device showed stable power output for 300 s under AM1.5G operation (Figure S31). The higher *J*_SC_ and FF were attributed to the higher electron mobility of CBBC‐TIC **1a** (3.9 × 10^−5^ cm^2^ V^−1^s^−1^) compared to that of BP‐TIC **8** (2.0 ×10^−5^ cm^2^ V^−1^s^−1^), whereas the higher *V*_OC_ is likely due to the higher uniformity of the film, which facilitates effective hole blocking and reduces charge recombination. While the above examination was conducted using racemic CBBC‐TIC **1a**, we also examined one of its enantiomers, (*P*,*P*)‐**1a**. However, the device exhibited a lower PCE of approximately 17% (Figure S32 and Table S6), even though our theoretical calculations suggest that the electron mobility of the enantiomer is comparable to that of the racemate (Figure S28). This result suggests that the lower PCE is not simply explained by the intrinsic electron mobility of the enantiopure material. Instead, device‐level factors, such as film formation and interfacial properties, may contribute to the observed performance difference.

**TABLE 1:** Photovoltaic parameters of the PSCs that use CBBC‐TICs **1a**, **1b**, **1c**, BP‐TIC **8**, or PCBM as ETMs.

ETM	Scan	*J*_SC_ (mA cm^−2^)^a^	*V*_OC_ (V)^a^	FF^a^	PCE (%)^a^
CBBC‐TIC **1a**	Forward	24.5 (24.6 ± 0.2)	1.08 (1.06 ± 0.02)	0.77 (0.75 ± 0.02)	20.6 (19.5 ± 0.7)
	Reverse	24.5 (24.5 ± 0.2)	1.09 (1.07 ± 0.01)	0.78 (0.76 ± 0.02)	20.6 (19.8 ± 0.7)
4Me‐CBBC‐TIC **1b**	Forward	22.3 (21.5 ± 0.5)	0.90 (0.89 ± 0.01)	0.70 (0.69 ± 0.01)	14.1 (13.1 ± 0.5)
	Reverse	22.3 (21.5 ± 0.5)	0.92 (0.91 ± 0.00)	0.73 (0.72 ± 0.01)	14.9 (14.0 ± 0.5)
4Me‐CBBC‐TIC **1b** ^b^	Forward	23.6 (23.6 ± 0.0)	1.02 (1.01 ± 0.03)	0.79 (0.77 ± 0.02)	19.1 (18.4 ± 1.0)
	Reverse	23.6 (23.5 ± 0.0)	1.03 (1.03 ± 0.01)	0.80 (0.80 ± 0.00)	19.4 (19.2 ± 0.3)
8Me‐CBBC‐TIC **1c**	Forward	23.2 (22.7 ± 0.4)	0.62 (0.60 ± 0.03)	0.67 (0.63 ± 0.04)	9.6 (8.6 ± 0.7)
	Reverse	23.0 (22.4 ± 0.4)	0.73 (0.74 ± 0.04)	0.65 (0.63 ± 0.01)	10.9 (10.5 ± 0.6)
8Me‐CBBC‐TIC **1c** ^b^	Forward	23.3 (23.2 ± 0.1)	0.92 (0.90 ± 0.02)	0.74 (0.72 ± 0.01)	15.8 (15.1 ± 0.6)
	Reverse	23.2 (23.2 ± 0.1)	0.93 (0.91 ± 0.02)	0.76 (0.76 ± 0.01)	16.5 (16.0 ± 0.5)
BP‐TIC **8**	Forward	23.7 (23.4 ± 0.2)	1.00 (1.03 ± 0.02)	0.66 (0.61 ± 0.03)	15.6 (14.6 ± 0.6)
	Reverse	23.6 (23.3 ± 0.2)	1.02 (1.04 ± 0.02)	0.72 (0.66 ± 0.03)	17.3 (15.9 ± 0.7)
PCBM	Forward	23.8 (23.2 ± 0.3)	1.08 (1.07 ± 0.01)	0.82 (0.81 ± 0.01)	21.1 (20.2 ± 0.5)
	Reverse	23.7 (23.2 ± 0.3)	1.08 (1.07 ± 0.01)	0.83 (0.82 ± 0.01)	21.2 (20.4 ± 0.4)

^a^ The average and standard‐deviation values for nine devices are given in parentheses.

^b^ The ETM layer was fabricated using a lower‐concentration solution (10 mg mL^−1^ instead of 15 mg mL^−1^).

The external quantum efficiency (EQE) spectra are shown in Figure 7c. The integrated *J*_SC_ values were 24.2, 23.3, and 23.9 mA cm^−2^ for devices using CBBC‐TIC **1a**, BP‐TIC **8**, and PCBM, respectively, which is in good agreement with the values obtained from the *J–V* curves. The statistical distribution of photovoltaic parameters obtained from nine cells measured in both forward and reverse scan directions is summarized in Figure 7d. For all parameters (*J*_SC_, *V*_OC_, and FF), the CBBC‐TIC‐based devices outperformed their BP‐TIC **8** counterparts, leading to a higher average PCE of 19.6 ± 0.7% compared to 15.2 ± 0.9% for the BP‐TIC‐based devices.

To better understand the differences in device performance, electrical impedance spectroscopy (EIS) was performed (Figure 7e). The complex impedance data were fitted using an equivalent circuit consisting of both series and parallel resistance elements along with a parallel capacitance. The series resistance (*R*_s_), estimated from the onset of the semicircle, was 2 Ω cm^2^ for the CBBC‐TIC **1a** device and 6 Ω cm^2^ for the BP‐TIC **8** device. The lower *R*_s_ for the CBBC‐TIC **1a** device can be attributed to its higher electron mobility compared to that of BP‐TIC **8**. The parallel resistance (*R*_p_), determined from the diameter of the main semicircle, was 168 Ω cm^2^ for CBBC‐TIC **1a** and 119 Ω cm^2^ for BP‐TIC **8**. The higher *R*_p_ of CBBC‐TIC **1a** indicates more effective suppression of current leakage and interfacial recombination, consistent with the enhanced *V*_OC_ and FF obtained in the solar cell devices. The comparison with BP‐TIC **8** is particularly informative because **8** can be regarded as a benzophenone‐based half‐unit reference of CBBC‐TIC **1a** and shows electronic properties similar to those of **1a**. Despite this electronic similarity, CBBC‐TIC **1a** forms a much smoother amorphous film, exhibits higher electron mobility, and affords higher PSC performance than BP‐TIC **8**. These results indicate that the X‐shaped nonplanar CBBC framework plays a critical role in suppressing excessive aggregation and enabling efficient electron transport in amorphous films. The CBBC‐TIC device also showed good performance under indoor‐lighting conditions (Figure 7f), achieving a PCE of 28.2% with a *J*_SC_ of 124 µA cm^−2^, a *V*_OC_ of 0.83 V, and an FF of 0.83 under 800 lux white LED illumination (0.30 mW cm^−2^; spectrum shown in Figure S33).

To further evaluate the structural tunability of the CBBC‐based ETM platform, PSCs incorporating tetramethyl‐CBBC‐TIC **1b** and octamethyl‐CBBC‐TIC **1c** were also fabricated (Table 1; Figures S34 and S35). Under the same fabrication conditions used for **1a**, the PCE gradually decreased with increasing numbers of methyl substituents, giving efficiencies of 14.9% for **1b** and 10.9% for **1c**. Interestingly, reducing the concentration of **1b** used for ETM fabrication from 15 to 10 mg mL^−1^ significantly improved device performance, affording a PCE of 19.4%, comparable to that obtained with **1a**. Octamethyl‐CBBC‐TIC **1c** also exhibited its highest performance when processed from a 10 mg mL^−1^ solution, yielding a PCE of 16.5% in the reverse scan. These results demonstrate that the CBBC framework can accommodate peripheral methyl substitution while retaining its functionality as a non‐fullerene ETM in inverted PSCs. At the same time, the strong concentration dependence of device performance and the lower efficiency observed for **1c** indicate that even subtle structural modifications can markedly affect film formation and device operation. Further systematic studies on the relationships among peripheral substitution, film morphology, and interfacial charge extraction will be important for optimizing the performance of CBBC‐based ETMs.

### 2.5 CISS Properties

To investigate whether the homochiral CBBC‐TIC **1a** exhibits spin‐selective electron transport, enantiopure thin films of (*M*,*M*)‐**1a** and (*P*,*P*)‐**1a** were evaluated by spin‐polarized magnetic‐conductive atomic force microscopy (mc‐AFM) [36, 37, 38, 39, 40]. In our previous study using the same experimental setup, a racemic sample showed essentially no CISS effect [40]. The films were prepared by spin‐coating on highly oriented pyrolytic graphite (HOPG), and the spin‐dependent current‐voltage responses (*I*_up_−*V* and *I*_down_−*V*) were measured using a ferromagnetic Co‐Cr cantilever under two opposite magnetization directions (Figure 8a). For each sample, approximately 200 current–voltage curves were recorded at different positions on the film under the two magnetization conditions (Figure S36), and the averaged *I*_up_–*V* and *I*_down_–*V* curves were used for analysis (Figures 8b and S36c,f). For (*M*,*M*)‐**1a**, the averaged *I*_up_–*V* curve showed consistently higher currents than the averaged *I*_down_–*V* curve, whereas the opposite trend was observed for (*P*,*P*)‐**1a** (Figures 8b and S36c,f). Such an enantiomer‐dependent inversion of the magnetization‐dependent current response is a hallmark of the CISS effect. The mean EMA values, which are defined as (*I*_up_–*I*_down_)/(*I*_up_ + *I*_down_)×100, were +80.1 ± 7.5% for (*M*,*M*)‐**1a** and −76.6 ± 3.4% for (*P*,*P*)‐**1a**, respectively (Figure 8c). Notably, these EMA values are high for amorphous chiral organic electronic materials [38, 39, 40, 41, 42]. While the quantitative determination of EMA values by mc‐AFM inevitably involves some uncertainty owing to the inherent experimental challenges, especially for amorphous films with non‐uniform surfaces, the overall trend and magnitude of the observed spin selectivity sufficiently highlight the potential of CBBC‐TIC **1a** as a platform that integrates both electron‐transporting and spin‐filtering functions within a single molecular architecture.

**FIGURE 8:**
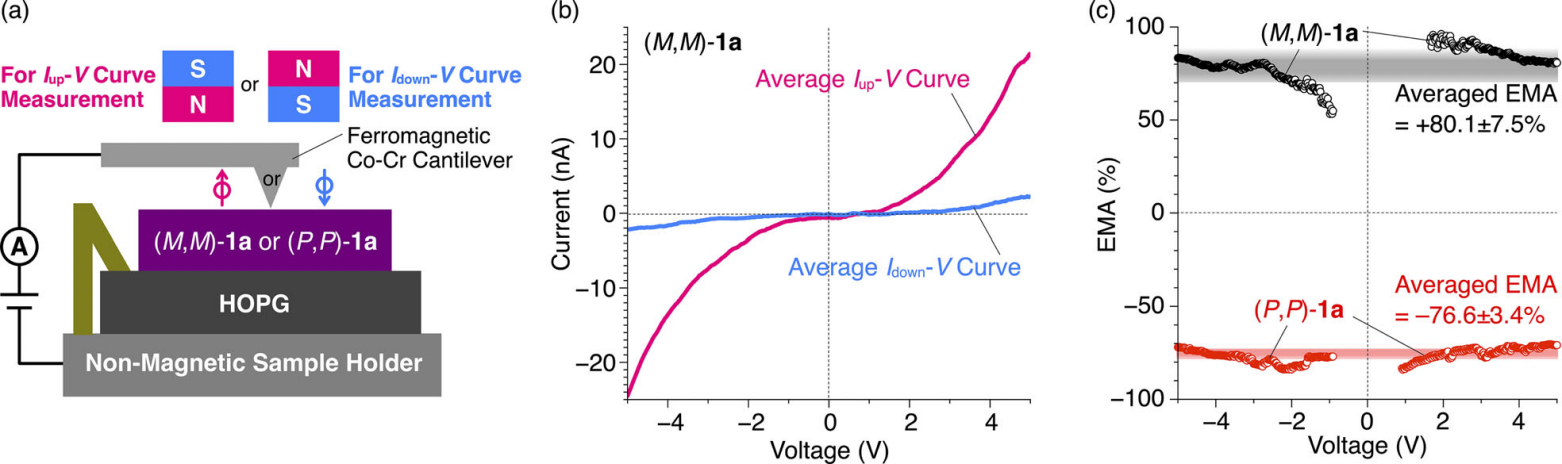
(a) Experimental setup for *I*_up_–*V* and *I*_down_–*V* curve measurements using mc‐AFM with a CoCr ferromagnetic cantilever. (b) Averaged *I*_up_–*V* and *I*_down_–*V* curves of (*M*,*M*)‐**1a**. (c) Enantiospecific magnetic conductance asymmetry (EMA) of (*M*,*M*)‐**1a** (black circles) and (*P*,*P*)‐**1a** (red circles). The EMA values in the voltage ranges of −1 to +2 V for (*M*,*M*)‐**1a** and −1 to +1 V for (*P*,*P*)‐**1a** are not displayed because both currents approach zero in this region, causing large uncertainties in the calculated EMA values due to the division of near‐zero signals. The original, unfiltered data are provided in Figure S37.

## 3 Conclusion

We developed a single‑molecule n‐type semiconductor, CBBC‑TIC, incorporating a figure‑eight CBBC core. CBBC‑TIC adopts an X‑shaped molecular geometry featuring a propeller‑like arrangement of electron‑withdrawing TIC units. This molecule exhibits strong light‑harvesting capability, with absorption extending to approximately 640 nm, whilst its reduction potential is comparable to that of PCBM. Owing to its three‑dimensional structure, CBBC‑TIC **1a** enables the formation of smooth, electron‑conductive amorphous films via solution processing. In contrast, the reference benzophenone analogue BP‑TIC **8** forms relatively rough films with inferior electron conductivity. Consequently, CBBC‑TIC **1a** functions effectively as an ETM in inverted PSCs, achieving a high PCE of 20.6%. Moreover, the chiral structure of CBBC‐TIC **1a** enables spin‑selective electron transport, exhibiting high EMA values of up to 80%. These results highlight the potential of the CBBC framework for the development of advanced non‑fullerene n‐type semiconductors.

## Author Contributions

**Yuki Sakamoto**: investigation. **Koki Otsuka**: investigation. **Kuraudo Ishihara**: investigation. **Shuang Li**: investigation. **Ai Shimazaki**: investigation. **Megumi Haramatsu**: investigation. **Junichiro Hirano**: investigation. **Juntaro Nogami**: investigation. **Hiroshi Shinokubo**: writing – review and editing, funding acquisition. **Hiroki Uratani**: formal analysis, writing – original draft, funding acquisition. **Hironori Kaji**: formal analysis, writing – review and editing, funding acquisition. **Fumitaka Ishiwari**: investigation, writing – review and editing. **Akinori Saeki**: writing – review and editing. **Tomoya Nakamura**: writing – original draft, conceptualization, investigation, formal analysis, funding acquisition. **Atsushi Wakamiya**: conceptualization, writing – review and editing, funding acquisition. **Norihito Fukui**: conceptualization, investigation, funding acquisition, writing – original draft, supervision, project administration.

## Conflicts of Interest

The authors declare no conflicts of Interest.

## Competing Financial Interest

A.W. is a Co‐founder and Chief Advisor (Science & Technology) of EneCoat Technologies Co., Ltd.

## Supporting information



Supporting Information is available. Experimental details, spectral data for all new compounds, and additional materials. The authors have cited additional references within Supporting Information [74–78]. **Supporting File**: anie74151‐sup‐0001‐SuppMat.pdf.

## Data Availability

The data that supports the findings of this study are available in the Supporting Information of this article.
