# Supplementary material for: An X‑Shaped Chiral Non‑Fullerene n‐Type Semiconductor: Application in Perovskite Solar Cells and Chirality‑Induced Spin Selectivity
Source: Angew Chem Int Ed Engl. 2026 Aug 26;65(43):e9296585. doi: 10.1002/anie.9296585 (PMC13676393; doi:10.1002/anie.9296585)
Supplement: Supplementary file 1 [file ANIE-65-e9296585-s001.pdf]

Table of Contents

1. Instrumentation and materials 2

2. Experimental procedures and compound data 4

3. NMR spectra 10

4. Mass spectra 24

5. Electrochemical properties 31

6. Racemization 32

7. CD spectra 33

8. DFT calculations 34

9. Cost of synthesis 42

10. Electron transport simulations 44

11. Fabrication of perovskite solar cells 46

12. Magnetic conductive-AFM measurement 53

13. References 55

1. Instrumentation and materials

Materials: All used reagents, solvents, and materials were purchased from Tokyo Chemical Industry (TCI), FUJIFILM Wako Pure Chemical Corporation, Sigma-Aldrich, Kanto Chemical, Nakalai Tesque, BLDpharm, or Alfa Aesar. Unless otherwise noted, materials obtained from commercial suppliers were used without further purification. Compounds **2**,[50] **4**,[74] **6a**–**6c**[75], and **9**[76] were synthesized according to the literature. Dry toluene and 1,4-dioxane were supplied from the Glass Contour solvent purification system. Unless otherwise mentioned, all reactions were performed under standard conditions at room temperature, at ambient pressure, and under air.

Chromatography: Analytical thin-layer chromatography was performed using fluorescent-labeled silica-coated plates (TLC silica gel 60 F_{254}, Merck). Detection was conducted using UV light (λ_{ex} = 254 nm). Flash column chromatography was performed using silica gel (Wakogel 60N (particle size: 63–212 µm), C-300 (particle size: 45–75 µm), or C-400HG (particle size: 20–40 µm)). Preparative resolution was conducted using a JAI LabACE LC-7080 plus preparative HPLC with a chiral column (DAICEL CHIRALPAK IA-3, φ 20 mm × 250 mm). Analytical HPLC separation was performed on a Shimadzu CBM-20A system with a UV detector (SPD-20A), and a Daicel Chiralpak IA-3 column placed in a column oven (CTO-20AC).

Nuclear magnetic resonance (NMR) spectroscopy: ^{1}H NMR (500 MHz) and ^{13}C NMR (126 MHz) spectra were recorded on a Bruker AVANCE III HD spectrometer. Chemical shifts were reported as the delta scale in ppm relative to TMS (δ = 0.00 ppm in $CDCl_3$) or solvent signal (δ = 6.00 ppm in 1,1,2,2-tetrachloroethane-d_2) for ^{1}H NMR and $CDCl_3$ (δ = 77.16 ppm) or 1,1,2,2-tetrachloroethane-d_2 (δ = 74.00 ppm) for ^{13}C NMR. The following abbreviations were used to describe the multiplicities: s = singlet, bs = broad singlet, d = doublet, t = triplet, q = quartet, quin = quintet, sext = sextet, sep = septet, m = multiplet.

Mass spectrometry (MS): High-resolution atmospheric pressure chemical ionization time-of-flight (APCI-TOF) and electrospray ionization time-of-flight (ESI-TOF) mass spectra were taken on a Bruker micrOTOF instrument. High-resolution matrix-assisted laser desorption and ionization time-of-flight (MALDI-TOF) mass spectra were taken on a Bruker autoflex maX.

Photophysical measurements: UV/vis/NIR absorption spectra were recorded on a JASCO V 670 spectrometer. Circular dichroism (CD) spectra were recorded on a JASCO J-1500 CD spectrometer.

Electrochemical measurements: Redox potentials were measured using cyclic voltammetry (CV) and differential pulse voltammetry (DPV) on an ALS electrochemical analyzer model 612C.

Voltammograms were obtained under the following conditions; solvent: CH_2Cl_2/TCE = 10/1, electrolyte: 0.1 M Bu_4NPF_6, working electrode: glassy carbon, counter electrode: Pt, reference electrode: Ag/$AgNO_3$. Scan rate of CV: 0.05 V/s.

DFT calculations: All calculations for single molecules were carried out using the *Gaussian 16* software package.[77] The structural optimization and frequency calculations were performed with the density functional theory (DFT) method with the restricted B3LYP[78] level, employing the 6-31G(d) basis sets. The TD-DFT calculations were conducted at the B3LYP/6-31G(d) level.

2. Experimental procedures and compound data

3,6,11,14-Tetrakis(4,4,5,5-tetramethyl-1,3,2-dioxaborolan-2-yl) CBBC 3

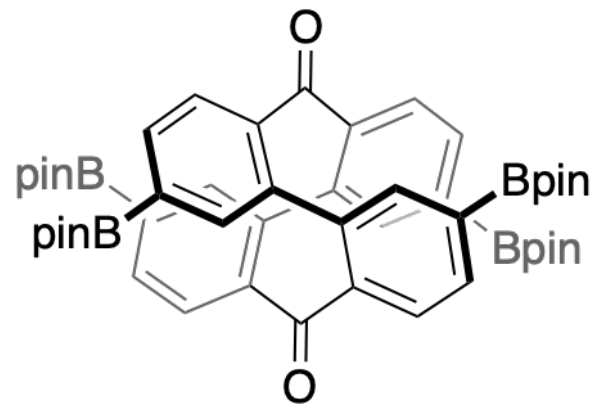

A Schlenk flask containing 3,6,11,14-tetrabromo CBBC **2** (3.4 g, 5.0 mmol), bis(pinacolato)diboron (6.1 g, 24 mmol), $PdCl_2(dppf)•CH_2Cl_2$ (0.49 g, 0.60 mmol), and potassium acetate (2.9 g, 30 mmol) was purged with N_2. To the mixture, degassed 1,4-dioxane (100 mL) was added. The mixture was stirred at 110 °C for 4 d. The reaction mixture was cooled to room temperature. The solvent was removed *in vacuo*. The mixture was purified by $SiO_2/B(OH)_3$ column chromatography (eluent: CH_2Cl_2). After removal of the solvent *in vacuo*, washing with hexane afforded compound **3** (3.9 g, 4.6 mmol, 91%) as a white solid.

^{1}H NMR (500 MHz, $CDCl_3$, 298 K): δ = 7.52 (dd, J_1 = 7.5, J_2 = 1.0 Hz, 4H), 7.42 (d, J = 7.5 Hz, 4H), 7.33 (d, J = 1.0 Hz, 4H), 1.31 (s, 48H) ppm; ^{13}C NMR (126 MHz, $CDCl_3$, 298 K): δ = 198.5, 140.9, 139.3, 137.0, 134.1, 132.1, 128.4, 84.2, 25.1, 24.8 ppm; HRMS (APCI): $[M+H]^+$ Calcd for $C_{50}H_{61}{}^{10}B_4O_{10}$ 862.4743; Found 862.4751.

Compound 5

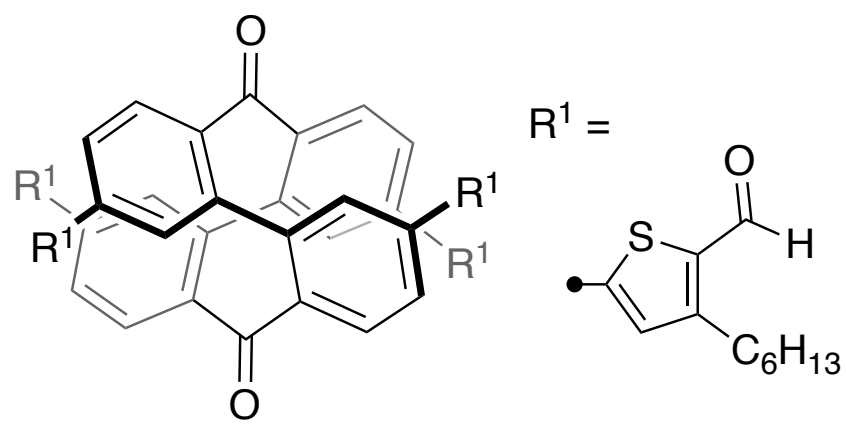

A Schlenk flask containing compound **3** (0.18 g, 0.21 mmol), **4** (0.47 g, 1.7 mmol), $Pd(PPh_3)_4$ (73 mg, 63 μmol), and potassium carbonate (0.35 g, 2.5 mmol) was purged with N_2. To the mixture, degassed dry toluene (4.2 mL), degassed dry EtOH (2.1 mL), and degassed H_2O (2.1 mL) were added. The mixture was stirred at 85 °C for 42 h. The reaction mixture was cooled to room temperature. The solvent was removed *in vacuo*. The mixture was purified by silica gel column chromatography (eluent: CH_2Cl_2/ethyl acetate = 100/1 to 100/3). After removal of the solvent *in vacuo*, recrystallization (CH_2Cl_2/MeOH) afforded compound **5** (0.14 g, 0.12 mmol, 58%) as a yellow solid.

^{1}H NMR (500 MHz, $CDCl_3$, 298 K): δ = 10.0 (s, 4H), 7.61 (d, J = 8.0 Hz, 4H), 7.44 (dd, J_1

= 8.0, J_2 = 2.0 Hz, 4H), 7.13 (s, 4H), 7.12 (d, J = 2.0 Hz, 4H), 2.95 (t, J = 7.5 Hz, 8H), 1.69 (q, J = 7.5 Hz, 8H), 1.45–1.25 (m, 24H), 0.89 (t, J = 8.0 Hz, 12H) ppm; ^{13}C NMR (126 MHz, $CDCl_3$, 298 K): δ = 195.6, 182.0, 154.0, 150.0, 140.5, 138.5, 138.3, 136.1, 130.9, 128.0 (overlap), 126.2, 31.6, 31.5, 29.2, 28.8, 22.7, 14.2 ppm; HRMS (APCI): $[M+H]^+$ Calcd for $C_{70}H_{73}O_6S_4$ 1137.4285; Found 1137.4261. The preparative resolution was conducted by recycling HPLC using a chiral column (Daicel Chiralpak IA-3: 20×250 mm, 254 nm UV detector, rt, eluent: CH_2Cl_2/hexane = 4/1, flow rate: 6 mL/min). Purities of the obtained enantiomers were determined by HPLC analysis using a chiral column (Daicel Chiralpak IA-3: 4.6×250 mm, 254 nm UV detector, 293 K, eluent: 50% CH_2Cl_2 in hexane, flow rate: 1.0 mL/min, retention time: 6.5 min for (*M*,*M*)-**5** and 11.0 min for (*P*,*P*)-**5**).

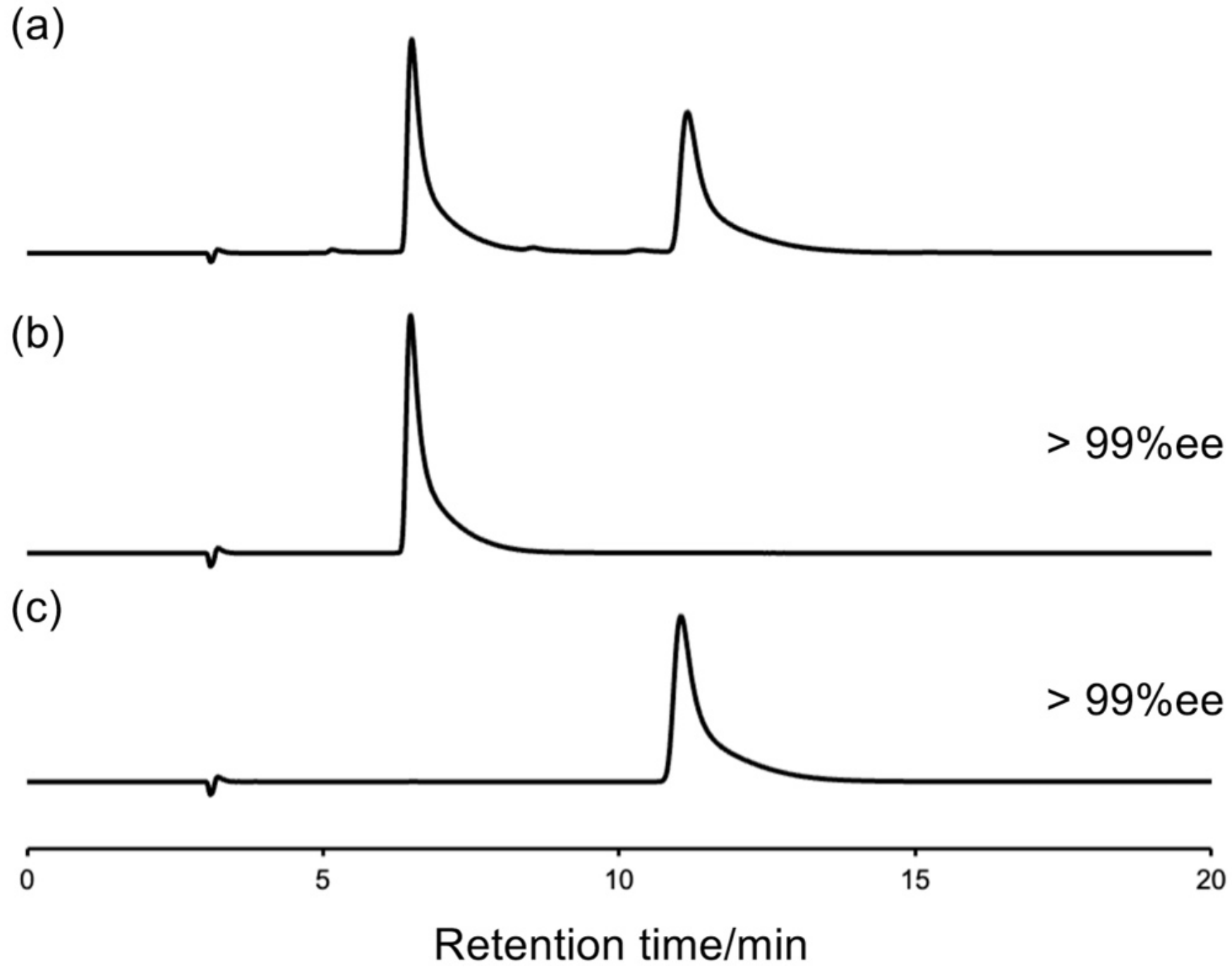

Figure S1. HPLC charts of isolated (a) *rac*-**5**, (b) (*M,M*)-**5**, (c) (*P,P*)-**5**.

CBBC-TIC 1a

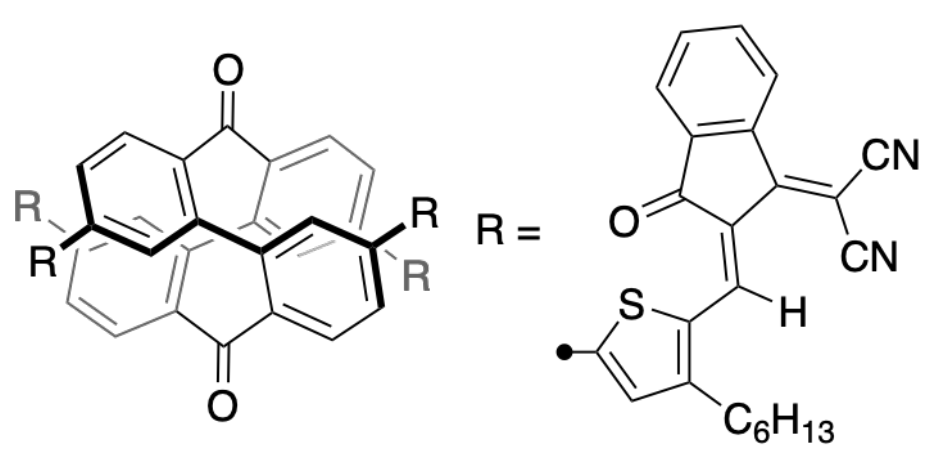

A Schlenk flask containing compound **5** (68 mg, 60 μmol) and compound **6a** (93 mg, 0.48

mmol) was purged with N_2. To the mixture, degassed dry pyridine (0.21 mL) and $CHCl_3$ (9.0 mL) were added. The mixture was refluxed for 19 h. The reaction mixture was cooled to room temperature, yielding red precipitates. The precipitates were collected by filtration and washed with $CHCl_3$. The obtained solid was dissolved in hot TCE. After cooling to room temperature, the solution was purified by silica gel column chromatography (eluent: $CHCl_3$ to $CHCl_3$/ethyl acetate = 50/3). After removal of the solvent *in vacuo*, the obtained solid was stirred in refluxed $CHCl_3$ (20 mL) for 16 h. After cooling to room temperature, the solid was collected by filtration, affording compound **1a** (90 mg, 49 μmol, 81%) as a red solid.

^{1}H NMR (500 MHz, tetrachloroethane-d_2, 298 K): δ = 8.91 (s, 4H), 8.58 (d, J = 8.0 Hz, 4H), 7.80 (d, J = 8.0 Hz, 4H), 7.75–7.65 (m, 8H), 7.64–7.55 (m, 8H), 7.31 (s, 4H), 7.28 (s, 4H), 2.91 (t, J = 7.5 Hz, 8H), 1.62–1.17 (m, 32H), 0.79 (t, J = 6.5 Hz, 12H) ppm; ^{13}C NMR (126 MHz, tetrachloroethane-d_2, 298 K): δ = 195.7, 188.3, 161.7, 161.3, 154.2, 140.3, 139.8, 138.9, 136.9, 135.9, 135.5, 134.9, 134.7, 132.9, 130.7, 128.4, 128.2, 126.6, 125.3, 124.0, 122.7, 115.0, 114.8, 69.7, 32.0, 31.7, 30.4, 29.3, 22.7, 14.3 ppm; HRMS (MALDI): $[M+H]^-$ Calcd for $C_{118}H_{88}N_8O_6S_4$ 1841.5782; Found 1841.5754. Purities of the enantiomers were determined by HPLC analysis using a chiral column (Daicel Chiralpak IA-3: 4.6×250 mm, 254 nm UV detector, 311 K, eluent: 45% CH_2Cl_2 in hexane, flow rate: 1.0 mL/min, retention time: 8.3 min for (*M*,*M*)-**1a** and 11.6 min for (*P*,*P*)-**1a**).

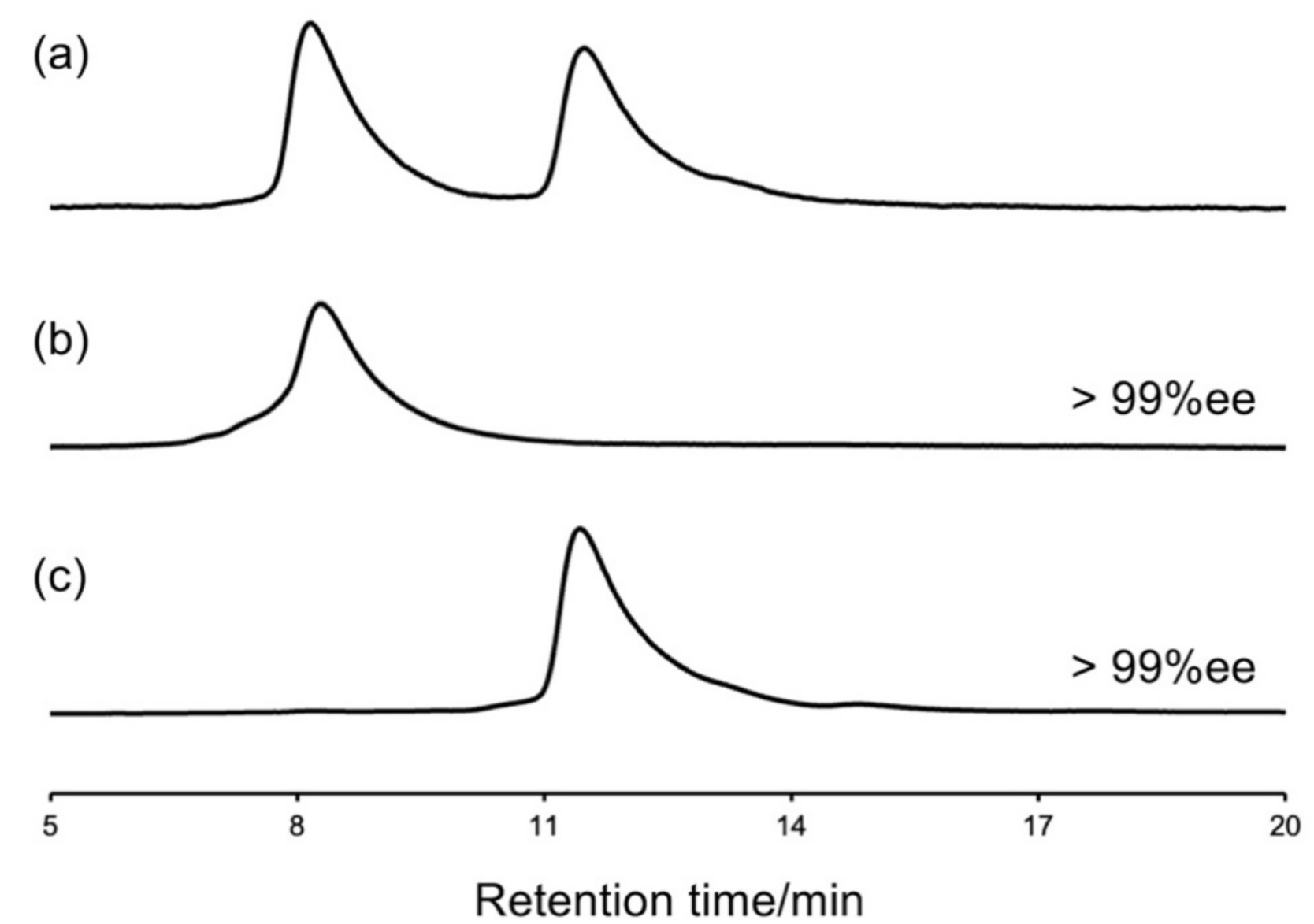

Figure S2. HPLC charts of isolated (a) *rac*-**1a**, (b) (*M,M*)-**1a**, and (c) (*P,P*)-**1a**.

CBBC-TIC 1b

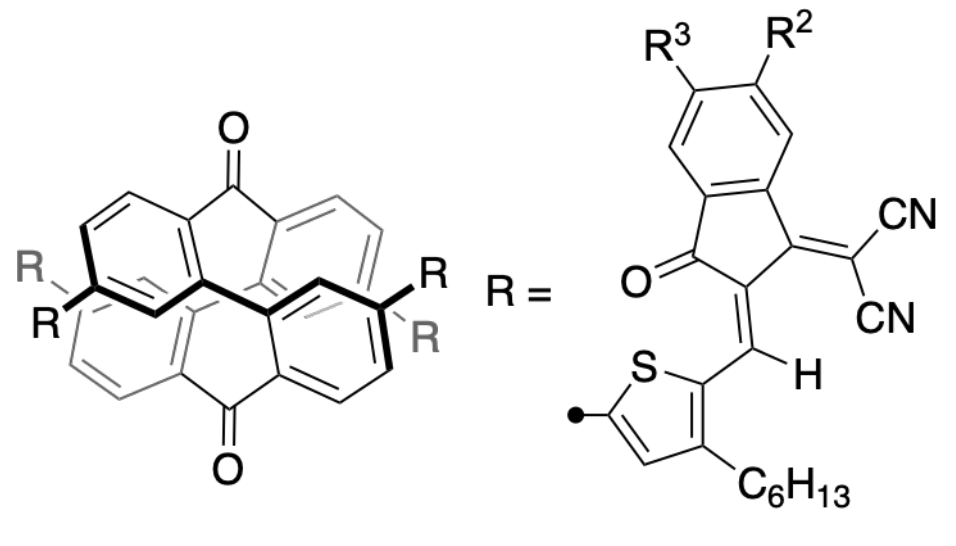

A Schlenk flask containing compound **5** (23 mg, 20 μmol) and compound **6b** (33 mg, 0.16 mmol, R^2/R^3 = H/CH_3 (42%) + CH_3/H (58%)) was purged with N_2. To the mixture, degassed dry pyridine (0.07 mL) and $CHCl_3$ (3.0 mL) were added. The mixture was refluxed for 17 h. The reaction mixture was cooled to room temperature. The crude mixture was directly subjected to silica gel column packed with CH_2Cl_2 as the solvent. Then, the solvent polarity was gradually increased to CH_2Cl_2/ethyl acetate = 50/1, eluting the target molecule. After removal of the solvent *in vacuo*, recrystallization (CH_2Cl_2/MeOH) afforded compound **1b** (33 mg, 17 μmol, 88%) as a red solid.

^{1}H NMR (500 MHz, $CDCl_3$, 296 K) δ = 8.97 (s), 8.54 (d, *J* = 8.5 Hz), 8.46 (s), 7.78 (d, *J* = 8.0 Hz), 7.72–7.50 (m), 7.42–7.30 (m), 2.98 (t, *J* = 8.0 Hz), 2.60–2.50 (m), 1.72 (br s), 1.44 (br s), 1.31 (br s), 0.88 (t, *J* = 7.0 Hz). (Because the target compound was obtained as the mixture of structural isomers, the observed signals are reported without assigning the numbers of protons.); ^{13}C NMR (126 MHz, $CDCl_3$, 298 K): δ = 187.9, 161.1, 140.4, 140.2, 137.5, 136.0, 135.8, 134.8, 134.3, 132.9, 128.1, 127.8, 126.2, 125.6, 123.7, 123.0, 114.8, 114.6, 31.9, 31.6, 30.3, 29.2, 22.6, 22.5, 22.1, 14.1 ppm (Because the target compound was obtained as the mixture of structural isomers, the number of observed signals is not consistent with that predicted for one isomer.); HRMS (MALDI): $[M+H]^+$ Calcd for $C_{122}H_{96}N_8O_6S_4$ 1897.6408; Found 1897.6481.

CBBC-TIC 1c

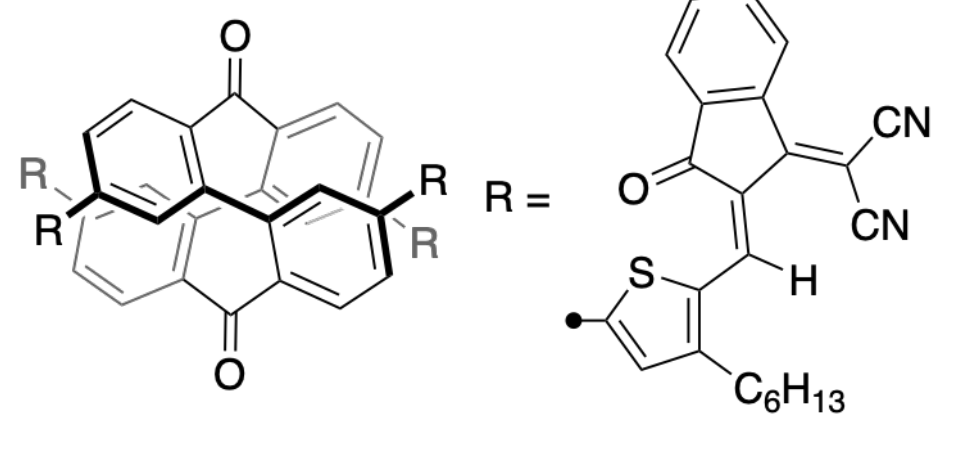

A Schlenk flask containing compound **5** (23 mg, 20 μmol) and compound **6c** (30 mg, 0.14 mmol) was purged with N_2. To the mixture, degassed dry pyridine (0.07 mL) and $CHCl_3$ (3.0 mL)

were added. The mixture was refluxed for 20 h. The reaction mixture was cooled to room temperature. The crude mixture was directly subjected to silica gel column packed with CH_2Cl_2 as the solvent. Then, the solvent polarity was gradually increased to CH_2Cl_2/ethyl acetate = 50/1, eluting the target molecule. After removal of the solvent *in vacuo*, recrystallization (CH_2Cl_2/MeOH) afforded compound **1c** (25 mg, 13 μmol, 64%) as a red solid.

^{1}H NMR (500 MHz, $CDCl_3$, 333 K): δ = 8.91 (s, 4H), 8.39 (s, 4H), 7.61 (d, *J* = 8.8 Hz, 12H), 7.35 (d, *J* = 3.3 Hz, 8H), 2.99 (t, *J* = 7.8 Hz, 8H), 2.46 (s, 12H), 2.44 (s, 12H), 1.74 (quin, *J* = 7.7 Hz, 8H), 1.46 (quin, *J* = 7.3 Hz, 8H), 1.36–1.27 (m, 20H), 0.88 (t, *J* = 7.1 Hz, 12H) ppm; ^{13}C NMR (126 MHz, $CDCl_3$, 323 K): *δ* = 153.70, 146.02, 145.66, 138.33, 135.73, 134.14, 133.18, 130.47, 128.49, 127.83, 126.29, 124.72, 115.05, 114.84, 31.99, 31.80, 30.43, 29.37, 22.71, 21.29, 20.84, 14.15 ppm (Several signals are not observed due to the weak intensity originating from the low solubility.); HRMS (MALDI): $[M+H]^+$ Calcd for $C_{126}H_{104}N_8O_6S_4$ 1953.7034; Found 1953.7010.

Compound S1

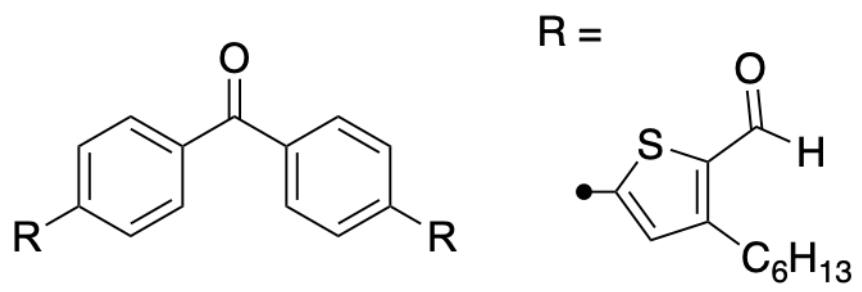

A Schlenk flask containing compound **9** (0.24 g, 0.50 mmol), compound **4** (0.55 g, 2.0 mmol), $Pd(PPh_3)_4$ (58 mg, 0.050 mmol), and potassium carbonate (0.50 g, 3.6 mmol) was purged with N_2. To the mixture, degassed dry toluene (8.0 mL), degassed dry EtOH (3.0 mL), and H_2O (3.0 mL) were added. The mixture was stirred at 80 °C for 24 h. The reaction mixture was cooled to room temperature. The solvent was removed in vacuo. The mixture was purified by silica gel column chromatography (eluent: CH_2Cl_2 to CH_2Cl_2/ethyl acetate = 9/1). After removal of the solvent in vacuo, compound **S1** (32 mg, 0.25 mmol, 11%) was obtained as a yellow solid.

^{1}H NMR (500 MHz, $CDCl_3$, 298 K): *δ* = 10.0 (s, 2H), 7.87 (d, *J* = 8.5 Hz, 2H), 7.79 (d, *J* = 8.5, 2H), 7.35 (s, 2H), 2.99 (t, *J* = 7.5 Hz, 4H), 1.72 (q, *J* = 7.5 Hz, 4H), 1.45–1.26 (m, 12H), 0.90 (t, *J* = 7.5 Hz, 6H) ppm; ^{13}C NMR (126 MHz, $CDCl_3$, 298 K): *δ* = 194.8, 182.1, 154.0, 151.1, 137.9, 137.6, 137.1, 131.0, 127.9, 126.3, 31.7, 13.6, 29.1, 28.8, 22.7, 14.2 ppm; HRMS (APCI): $[M]^+$ Calcd for $C_{35}H_{38}O_3S_2$ 570.2257; Found 570.2266.

Compound 8

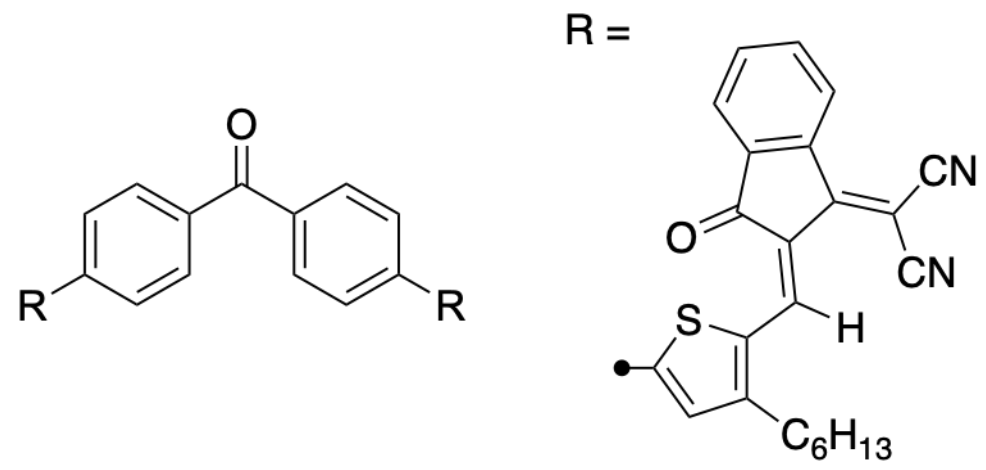

A Schlenk flask containing compound **S1** (0.12 g, 0.20 mmol) and compound **6** (0.17 g, 0.80 mmol) was purged with N_2. To the mixture, degassed dry pyridine (0.35 mL) and $CHCl_3$ (20 mL) were added. The mixture was stirred at 65 °C for 16 h. The reaction mixture was cooled to room temperature, yielding red precipitates. The precipitates were collected by filtration. The mixture was purified by silica gel column chromatography (eluent: $CHCl_3$). After removal of the solvent in vacuo, compound **8** (98 mg, 0.11 mmol, 53%) was obtained as a red solid.

^{1}H NMR (500 MHz, $CDCl_3$, 298 K): δ = 9.07 (s, 2H), 8.74 (d, J = 7.5 Hz, 2H), 8.00–7.86 (m, 10H), 7.84–7.74 (m, 4H), 7.52 (s, 2H), 3.03 (t, J = 8.0 Hz, 4H), 1.74 (q, J = 8.0 Hz, 4H), 1.50–1.25 (m, 12H), 0.91 (t, J = 7.0 Hz, 6H) ppm; ^{13}C NMR (126 MHz, $CDCl_3$, 298 K): δ = 194.8, 188.5, 161.6, 161.3, 155.4, 140.1, 138.0, 137.2, 137.1, 135.4, 135.0, 134.8, 132.8, 131.0, 128.0, 126.7, 125.6, 124.0, 122.6, 115.0, 114.7, 70.0, 32.1, 31.8, 30.4, 29.3, 22.7, 14.2 ppm; HRMS (MALDI): $[M+H]^+$ Calcd for $C_{59}H_{47}N_4O_3S_2$ 923.3084; Found 923.3112.

3. NMR spectra

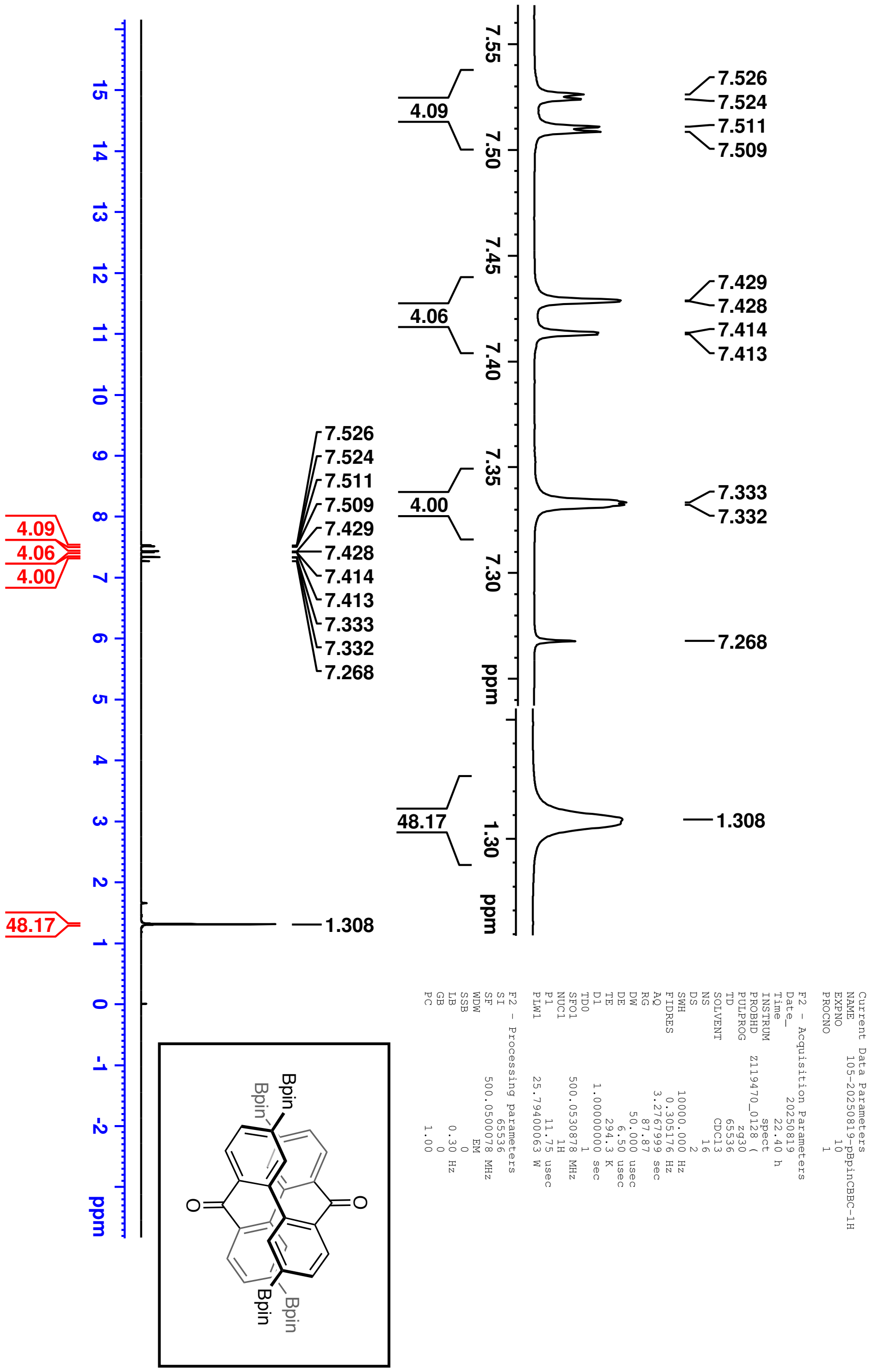

Figure S1. ^{1}H NMR spectrum of **3** in $CDCl_3$ at 25 °C.

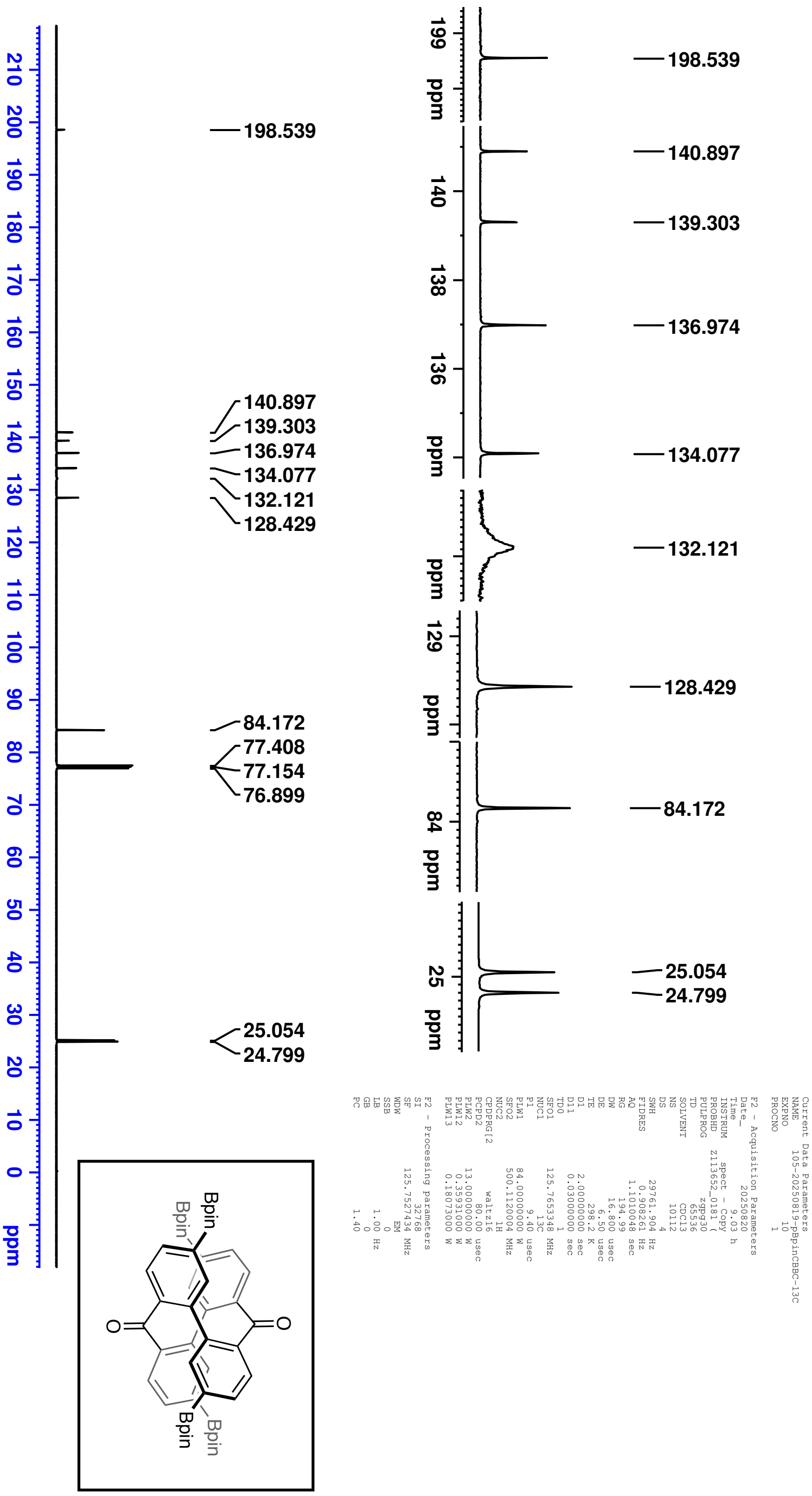

Figure S2. ^{13}C NMR spectrum of **3** in $CDCl_3$ at 25 °C.

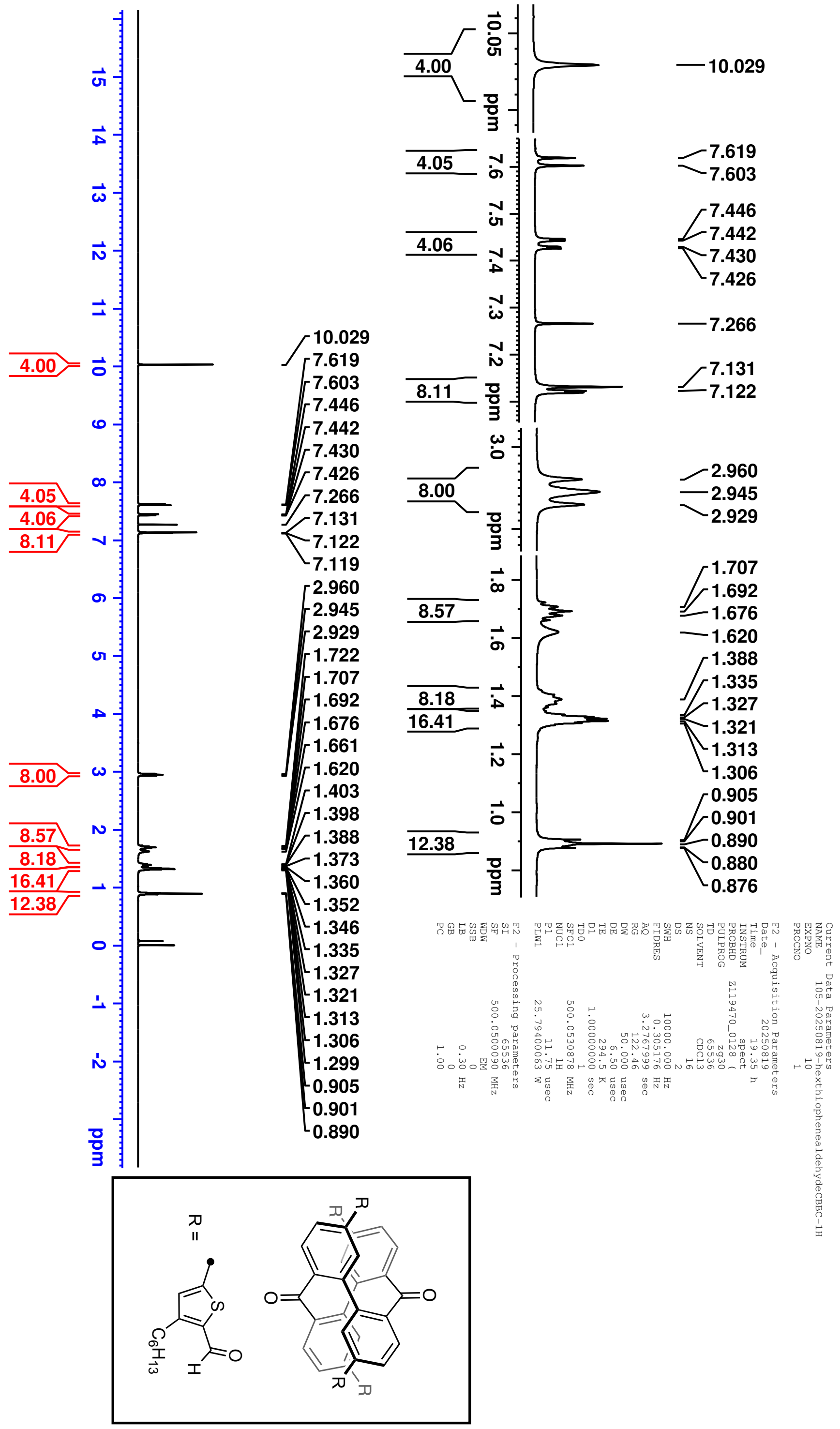

Figure S3. ^{1}H NMR spectrum of **5** in $CDCl_3$ at 25 °C.

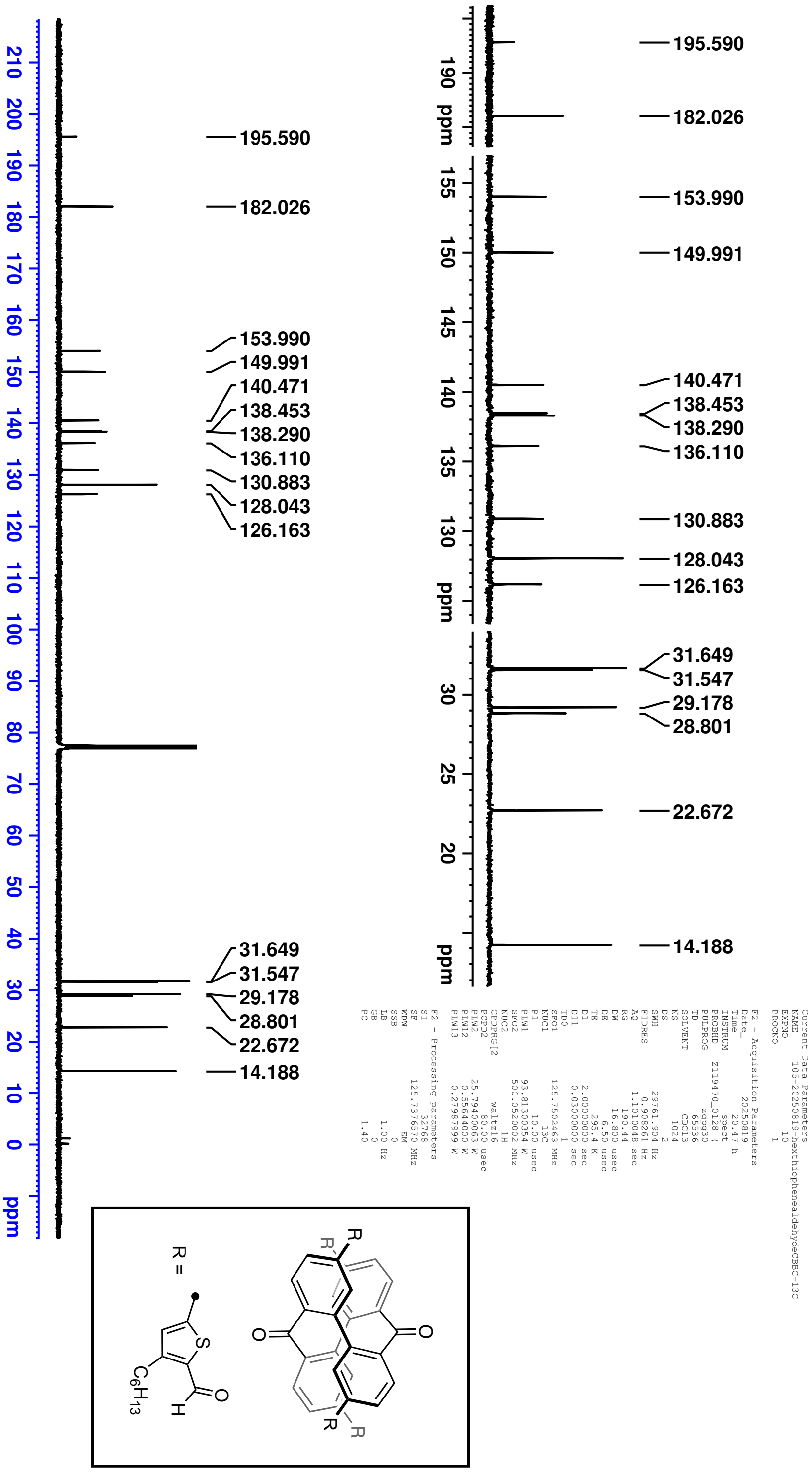

Figure S4. ^{13}C NMR spectrum of **5** in $CDCl_3$ at 25 °C.

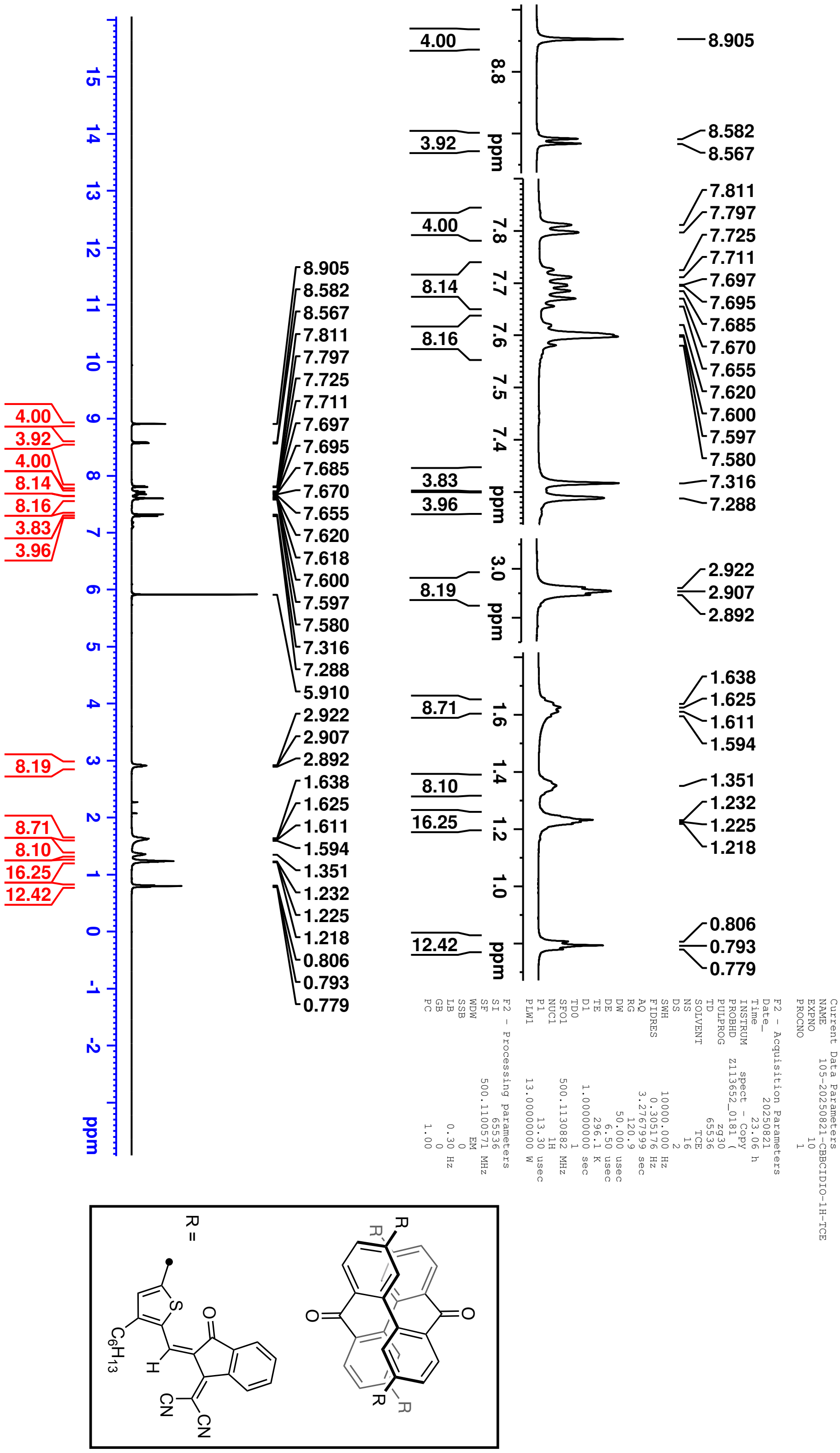

Figure S5. ¹H NMR spectrum of **1a** in tetrachloroethane-d_2 at 25 °C.

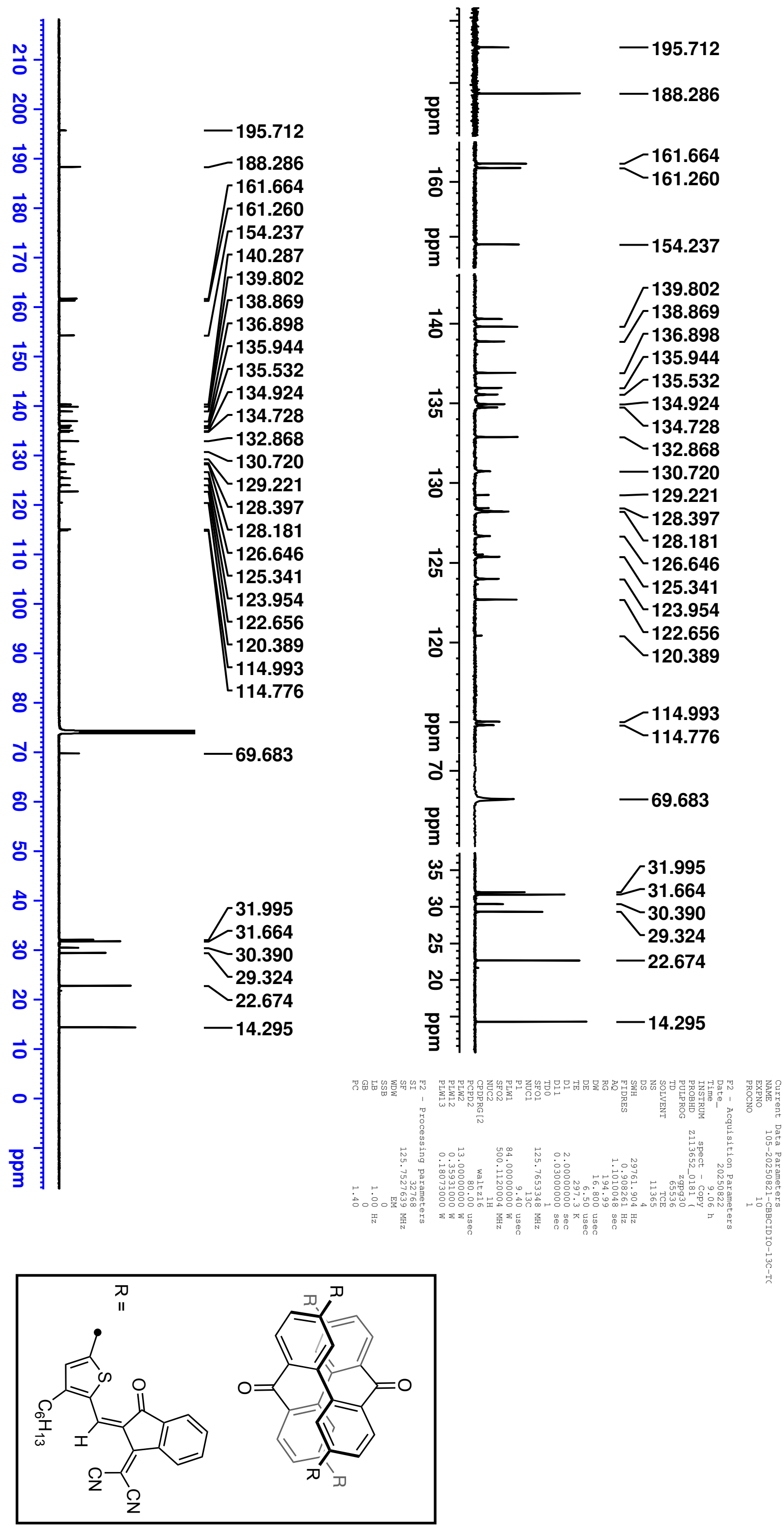

Figure S6. ^{13}C NMR spectrum of **1a** in tetrachloroethane-d_2 at 25 °C.

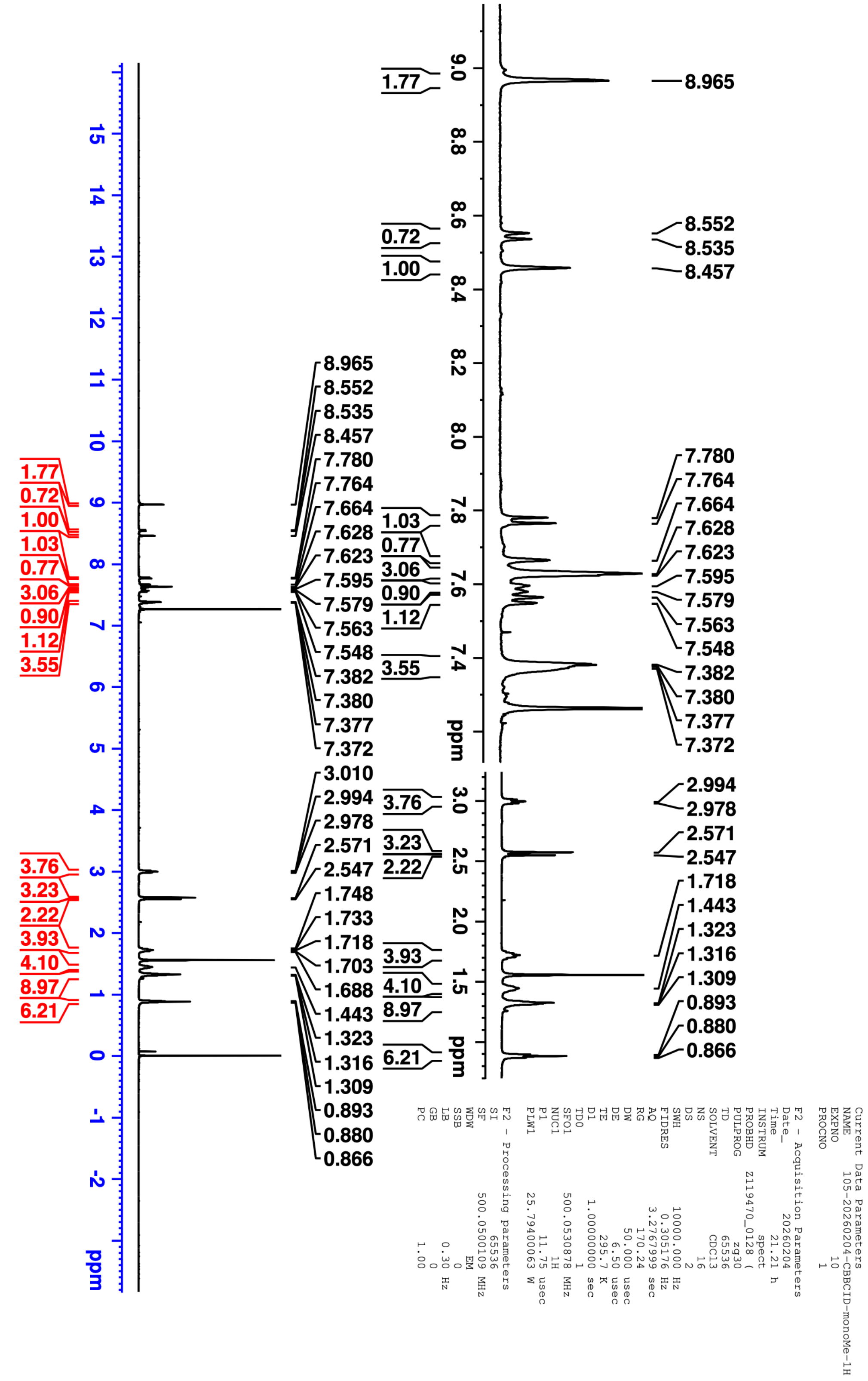

Figure S7. ^{1}H NMR spectrum of **1b** in $CDCl_3$ at 25 °C.

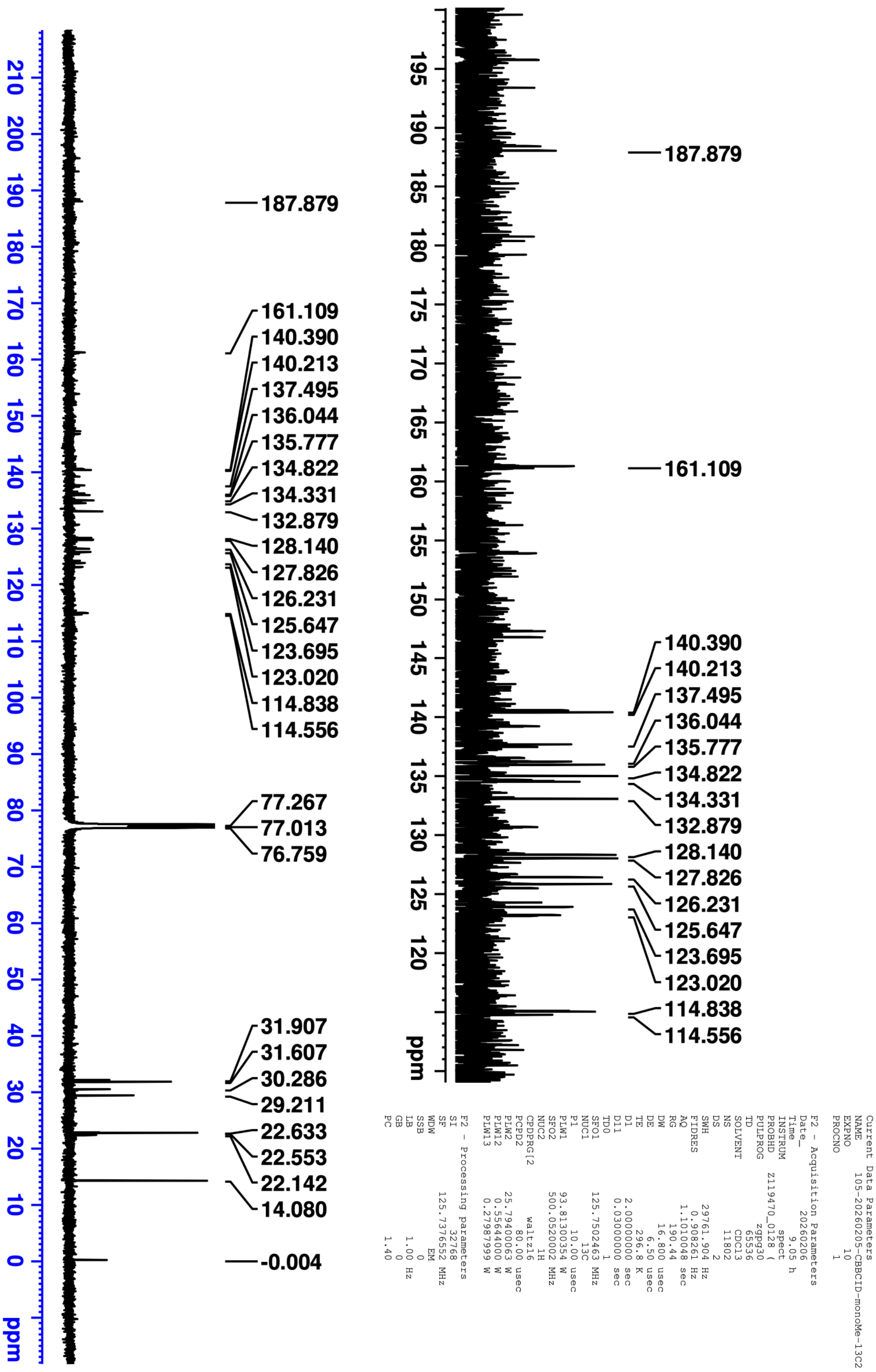

Figure S8. ^{13}C NMR spectrum of **1b** in $CDCl_3$ at 25 °C.

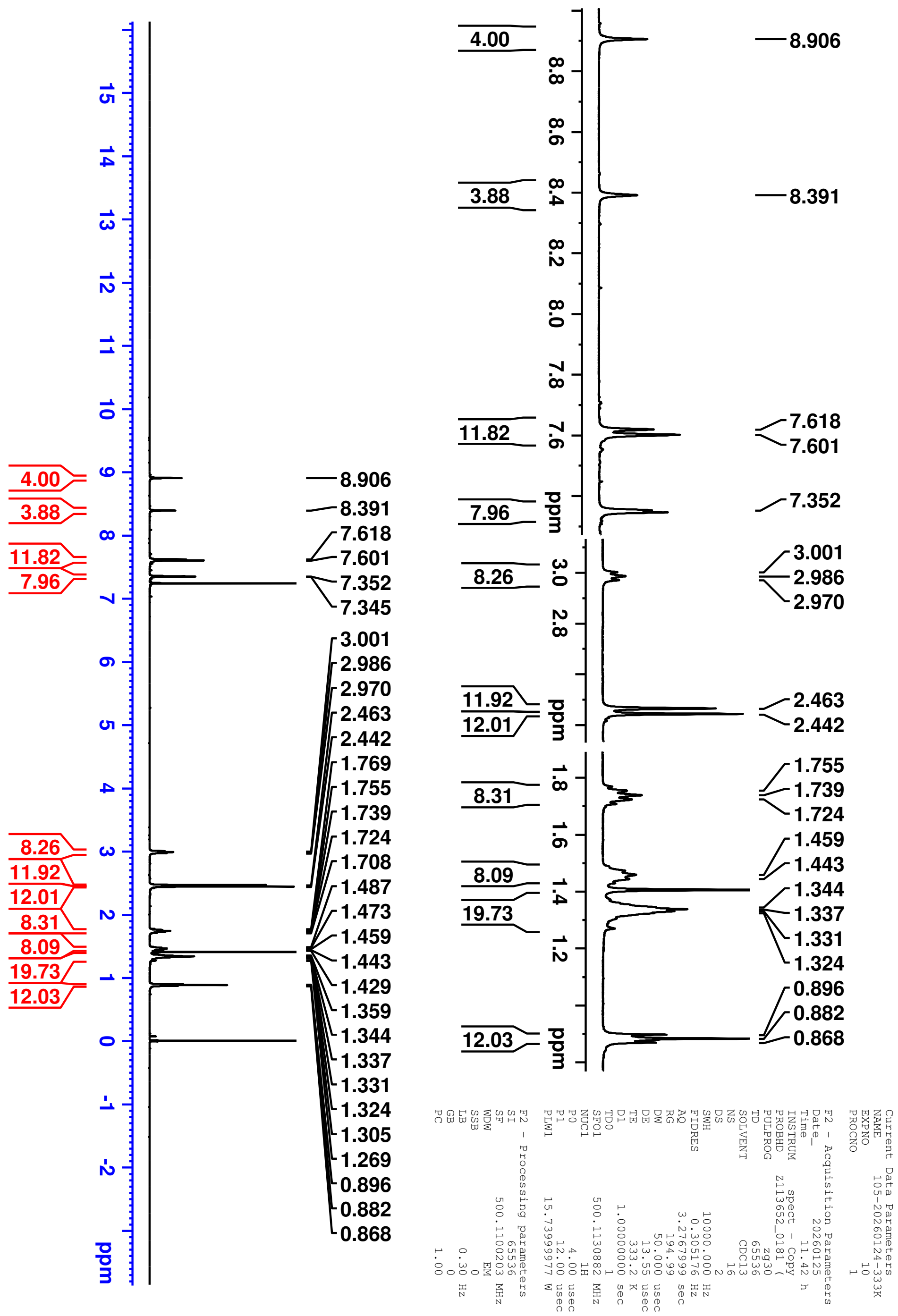

Figure S9. ^{1}H NMR spectrum of **1c** in $CDCl_3$ at 60 °C.

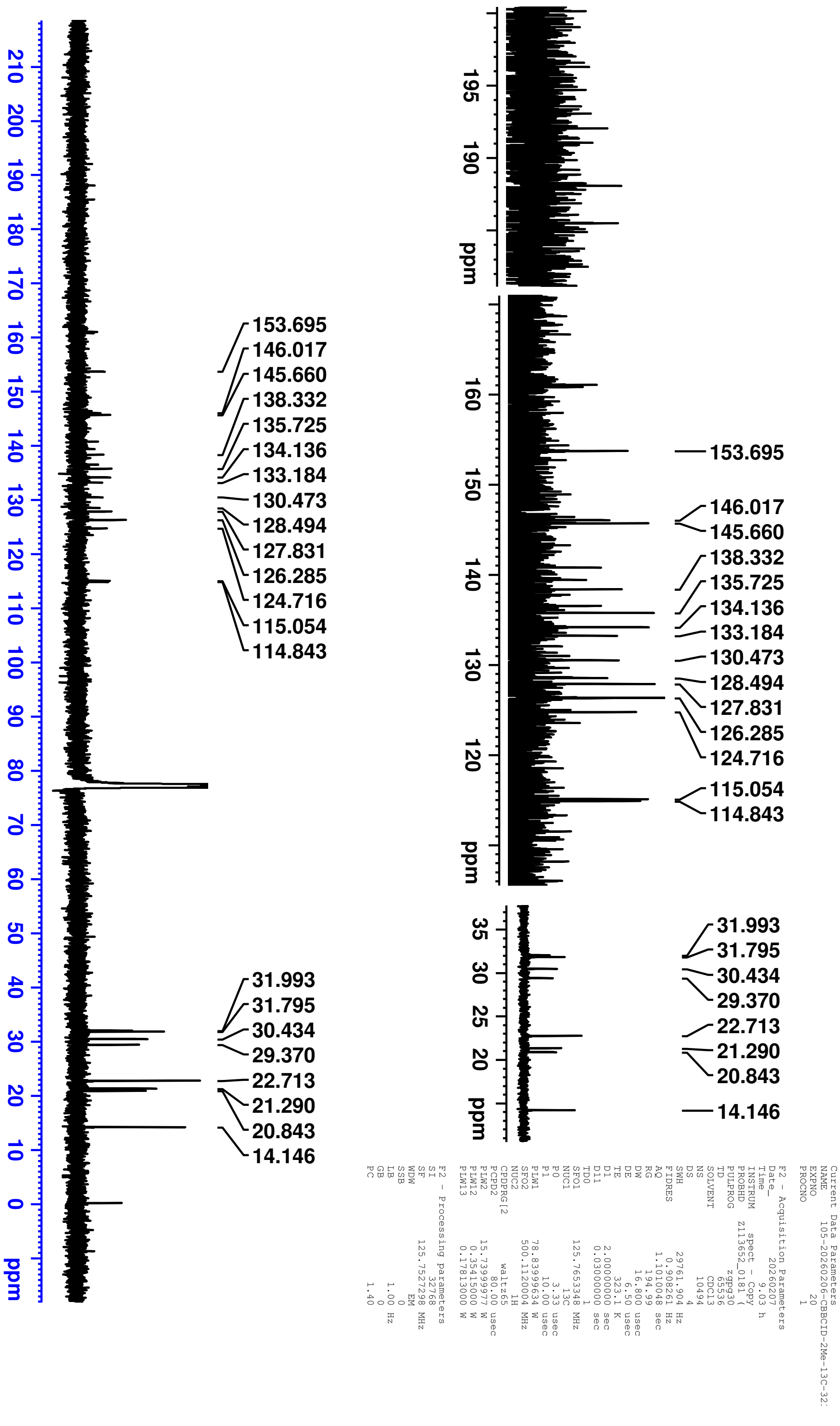

Figure S10. ^{13}C NMR spectrum of **1c** in $CDCl_3$ at 50 °C.

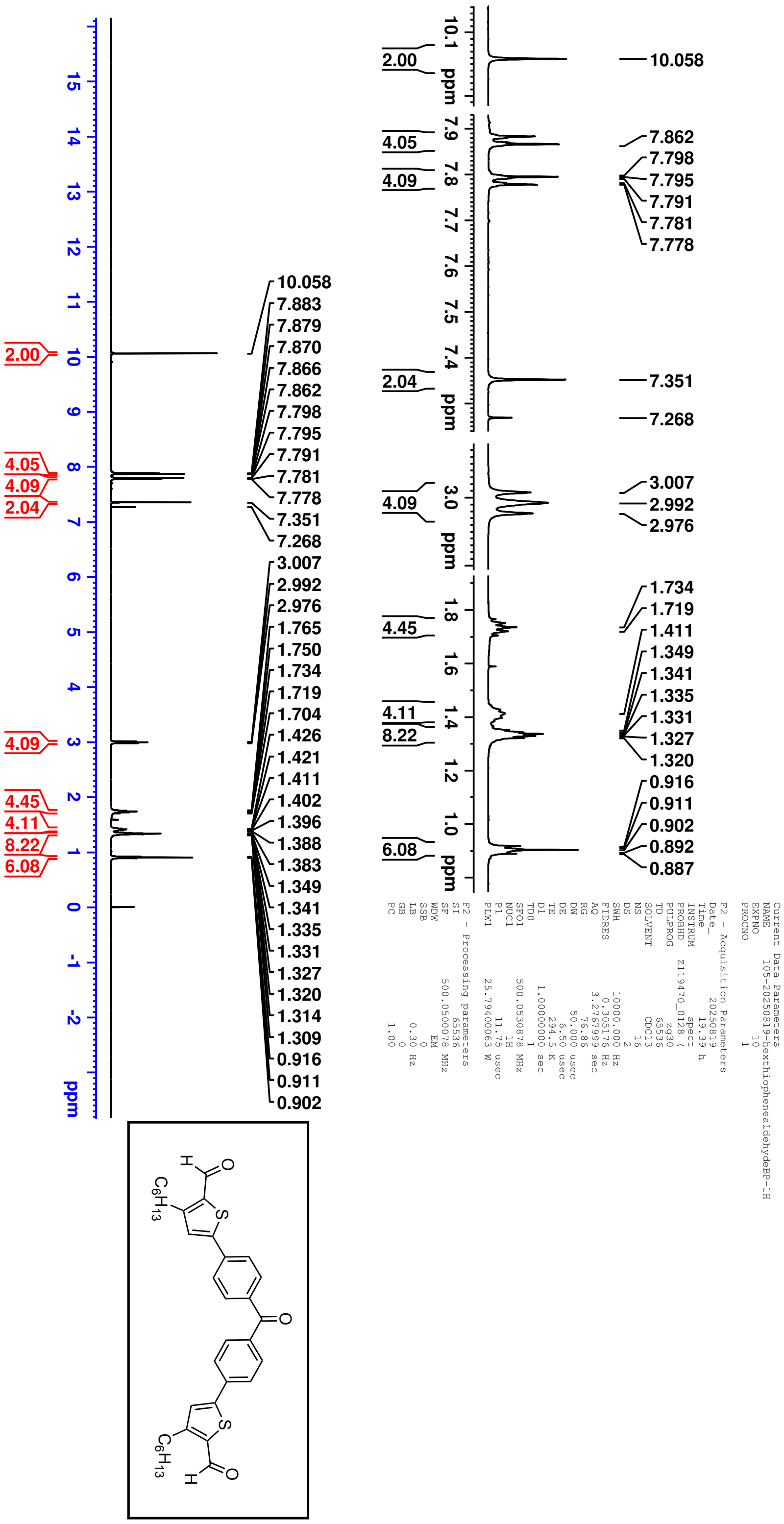

Figure S11. ^{1}H NMR spectrum of **S1** in $CDCl_3$ at 25 °C.

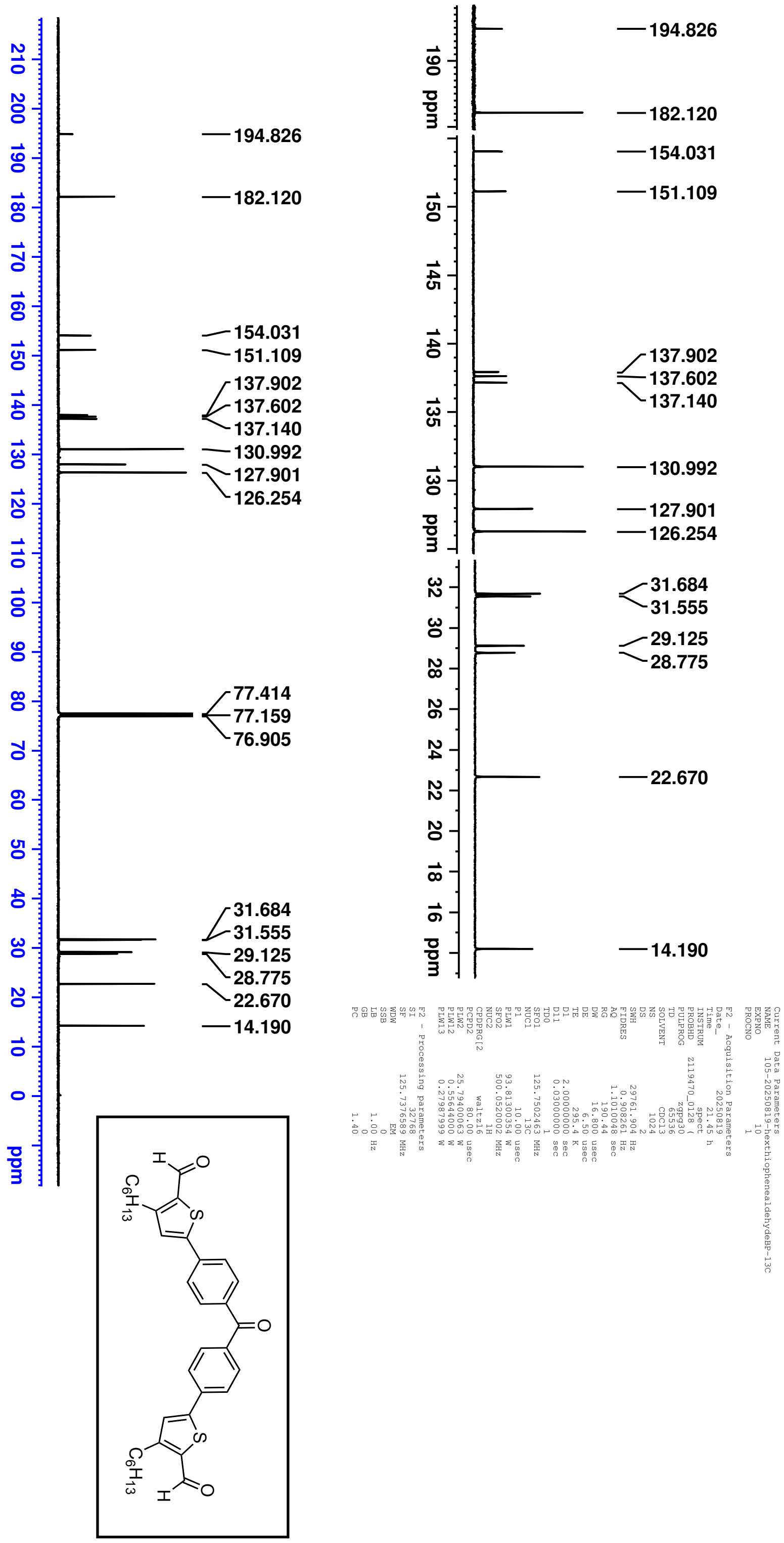

Figure S12. ^{13}C NMR spectrum of **S1** in $CDCl_3$ at 25 °C.

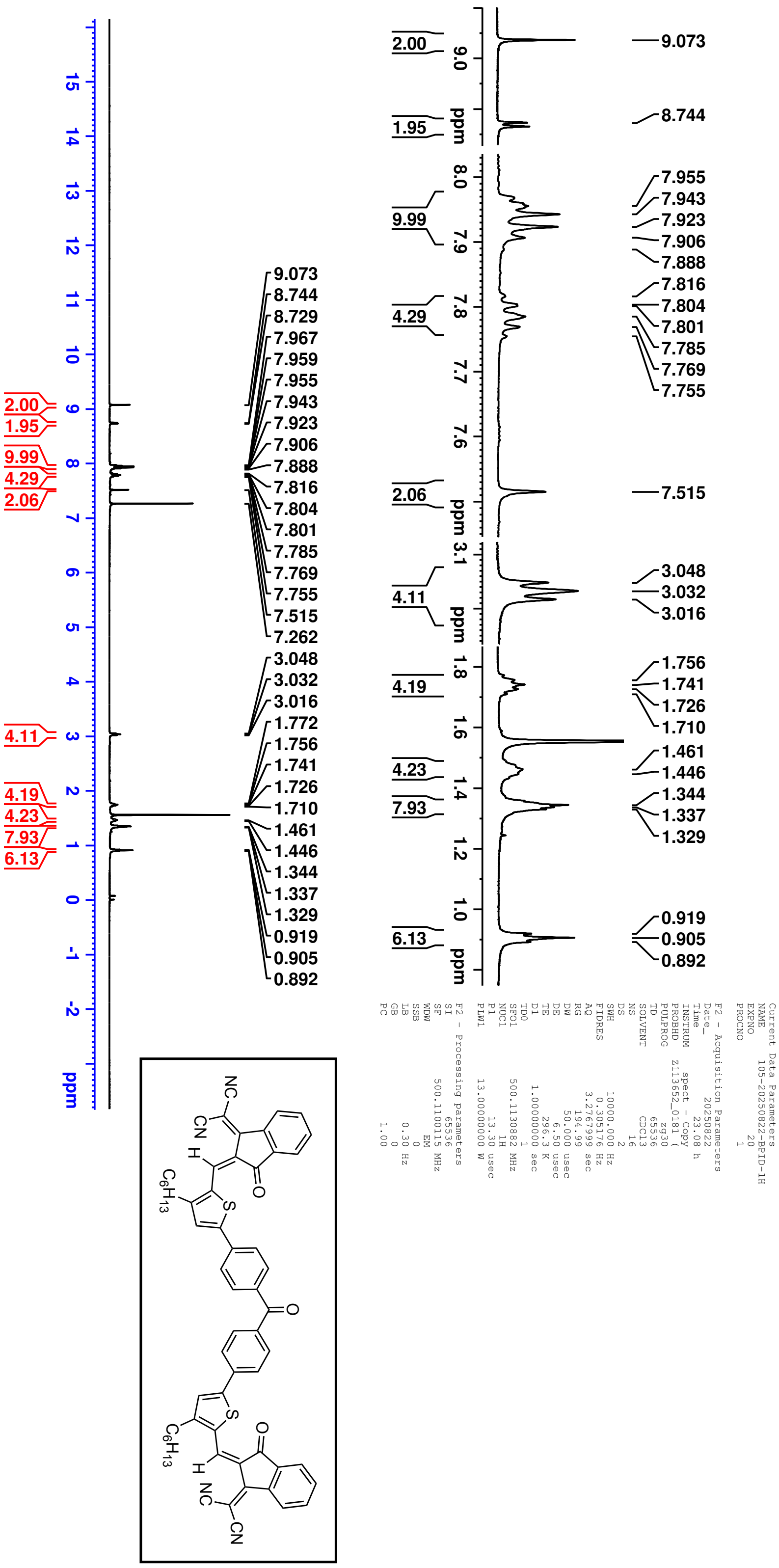

Figure S13. ^{1}H NMR spectrum of **8** in $CDCl_3$ at 25 °C.

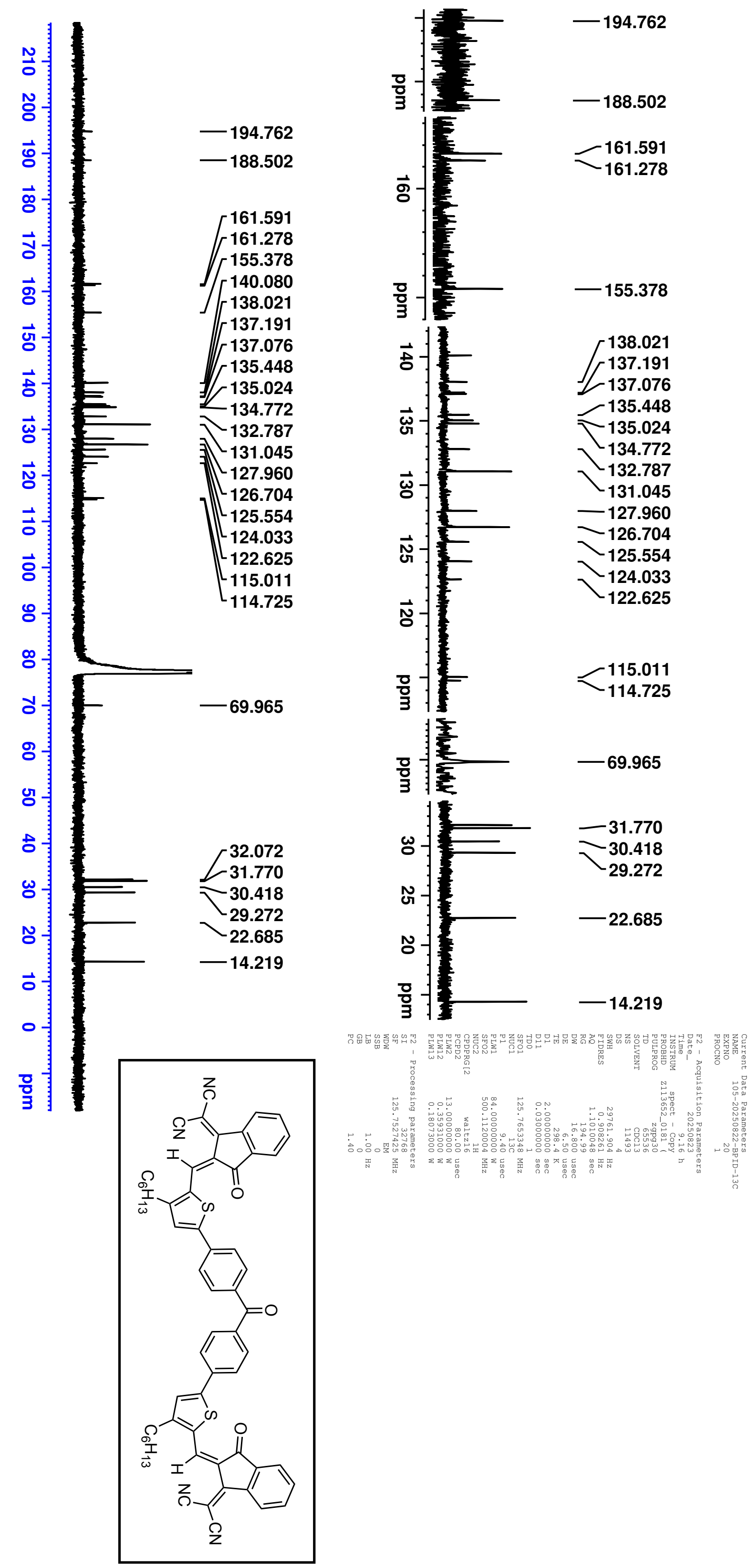

Figure S14. ^{13}C NMR spectrum of **8** in $CDCl_3$ at 25 °C.

4. Mass spectra

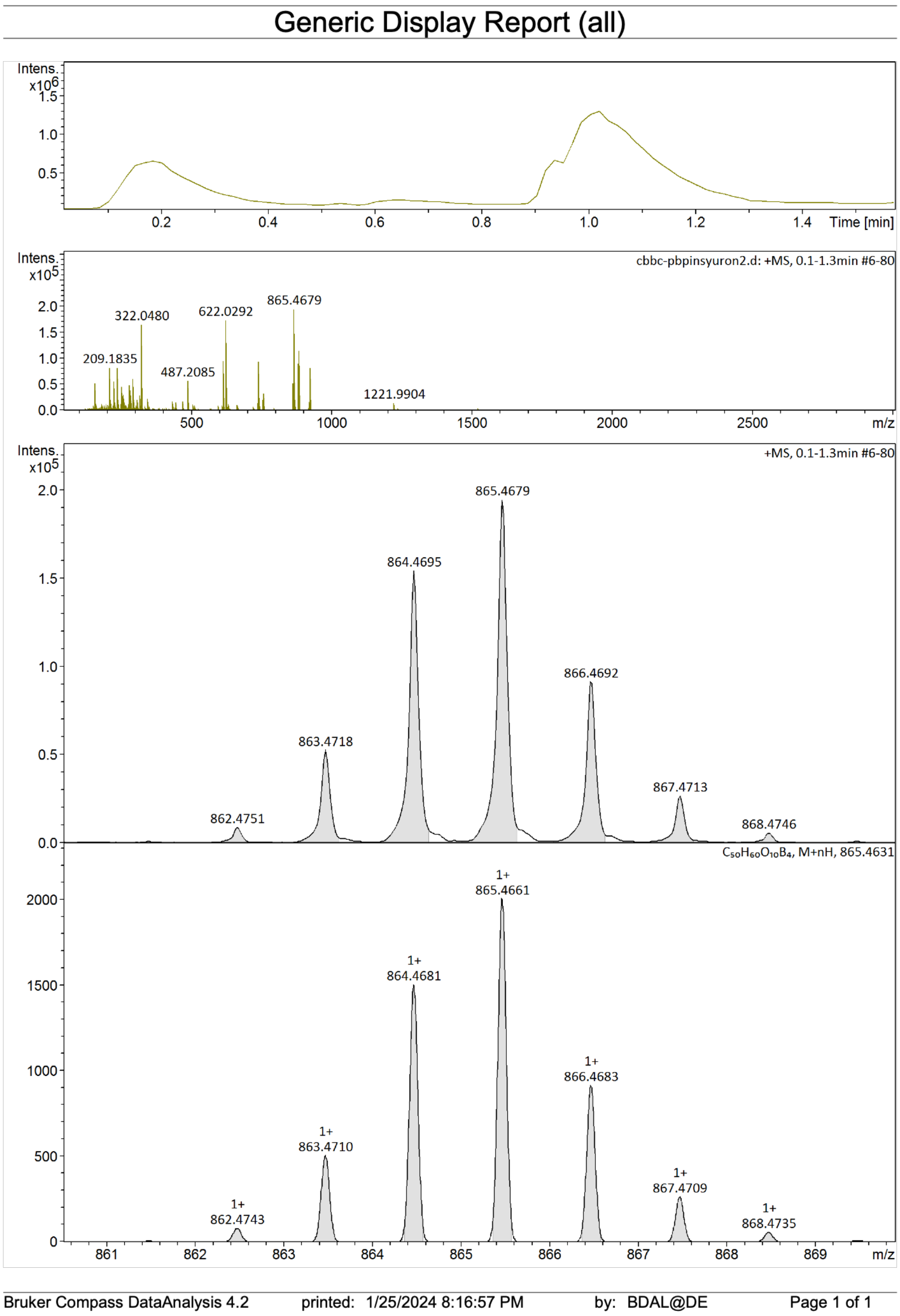

Figure S15. APCI-TOF mass spectrum of **3**.

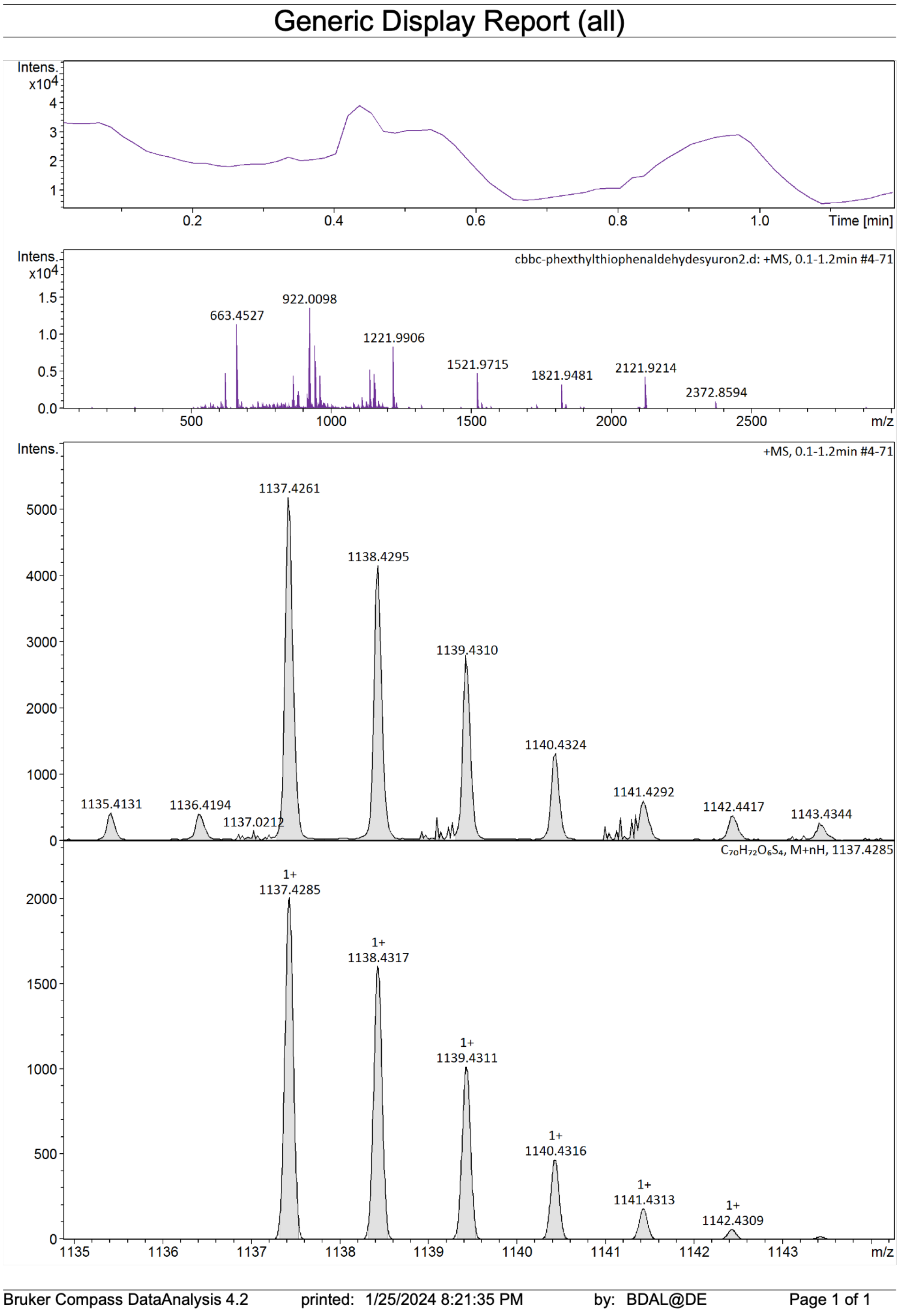

Figure S16. APCI-TOF mass spectrum of **5**.

Comment 1
Comment 2

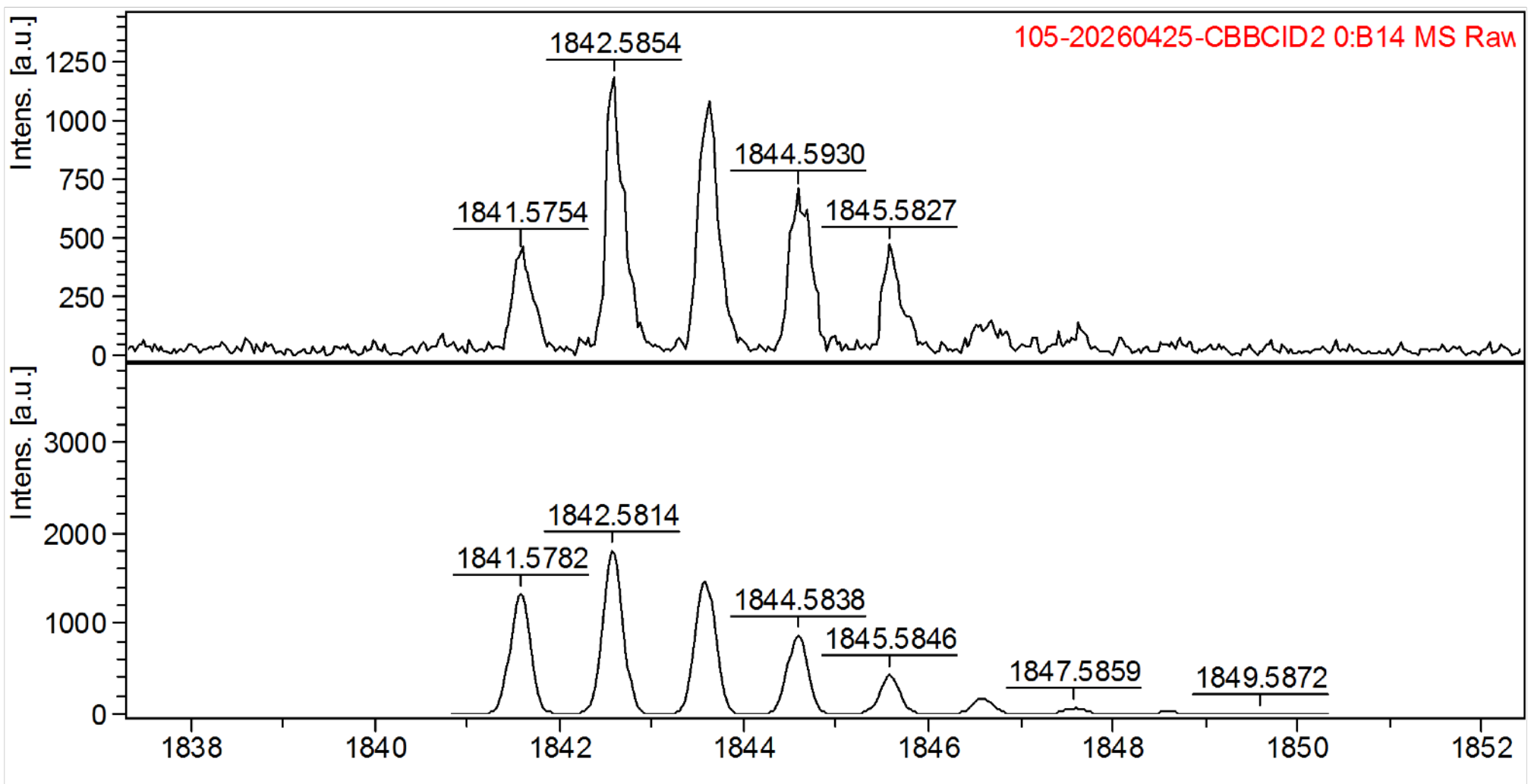

m/z	S/N	Quality Fac.	Res.	Intens.	Area
1841.5782			7414	1334	
1842.5814			7418	1800	
1843.5828			7422	1460	
1844.5838			7426	876	
1845.5846			7430	424	
1846.5853			7434	173	
1847.5859			7438	61.0	
1848.5865			7442	19.0	
1849.5872			7446	5.30	

Acquisition Parameter

Date of acquisition
Acquisition method name

Aquisition operation mode
Voltage polarity
Number of shots
Name of spectrum used for calibration
Calibration reference list used

Instrument Info

User	tof-user
Instrument	FLEX-PC
Instrument type	

Bruker Daltonics flexAnalysis printed: 4/25/2026 4:17:40 PM

Figure S17. MALDI-TOF mass spectrum of **1a**.

Comment 1
Comment 2

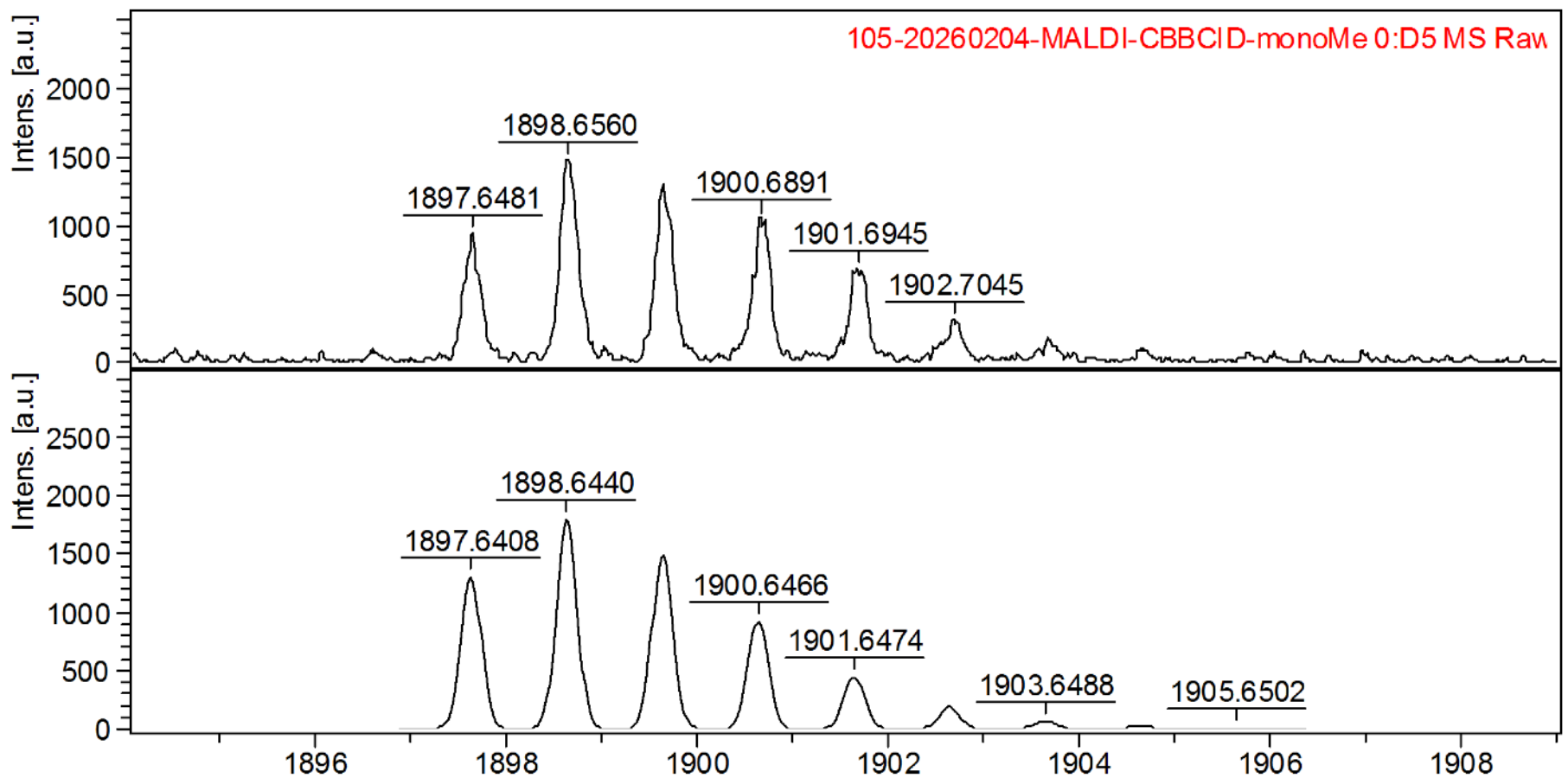

m/z	S/N	Quality Fac.	Res.	Intens.	Area
1897.6408			7639	1291	
1898.6440			7643	1800	
1899.6455			7648	1492	
1900.6466			7652	912	
1901.6474			7656	449	
1902.6482			7660	186	
1903.6488			7664	66.7	
1904.6495			7668	21.1	
1905.6502			7672	5.99	

Acquisition Parameter

Date of acquisition
Acquisition method name

Aquisition operation mode
Voltage polarity
Number of shots
Name of spectrum used for calibration
Calibration reference list used

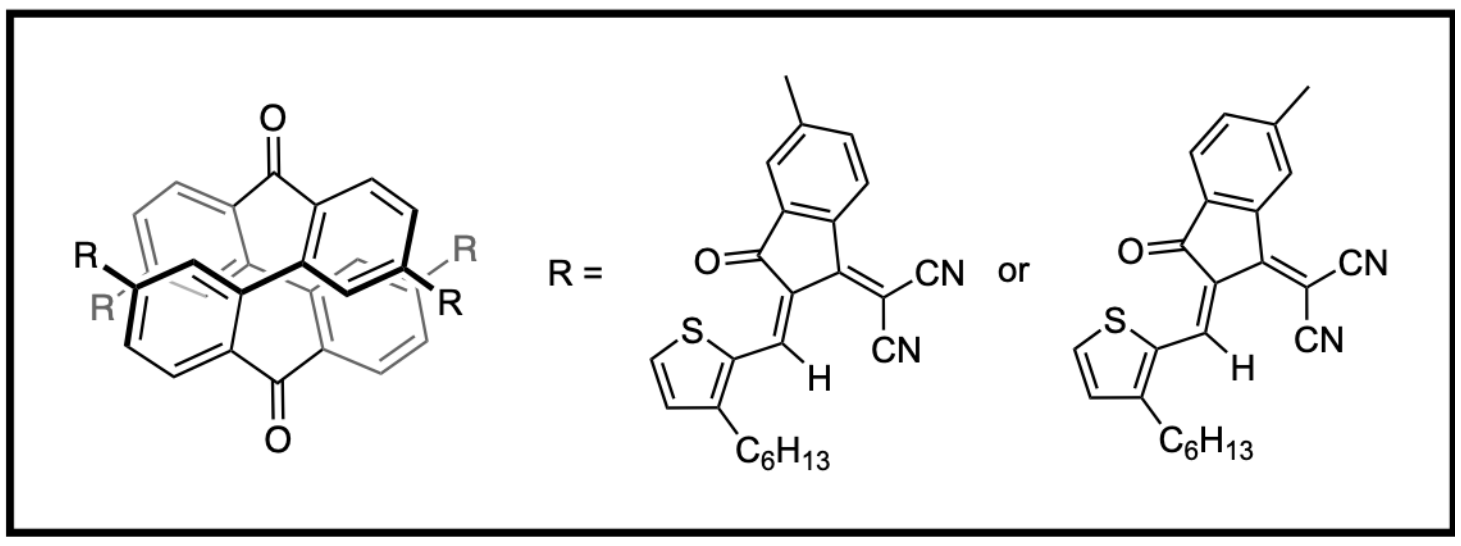

Instrument Info

User tof-user
Instrument FLEX-PC
Instrument type

Bruker Daltonics flexAnalysis printed: 2/4/2026 10:31:43 PM

Figure S18. MALDI-TOF mass spectrum of **1b**.

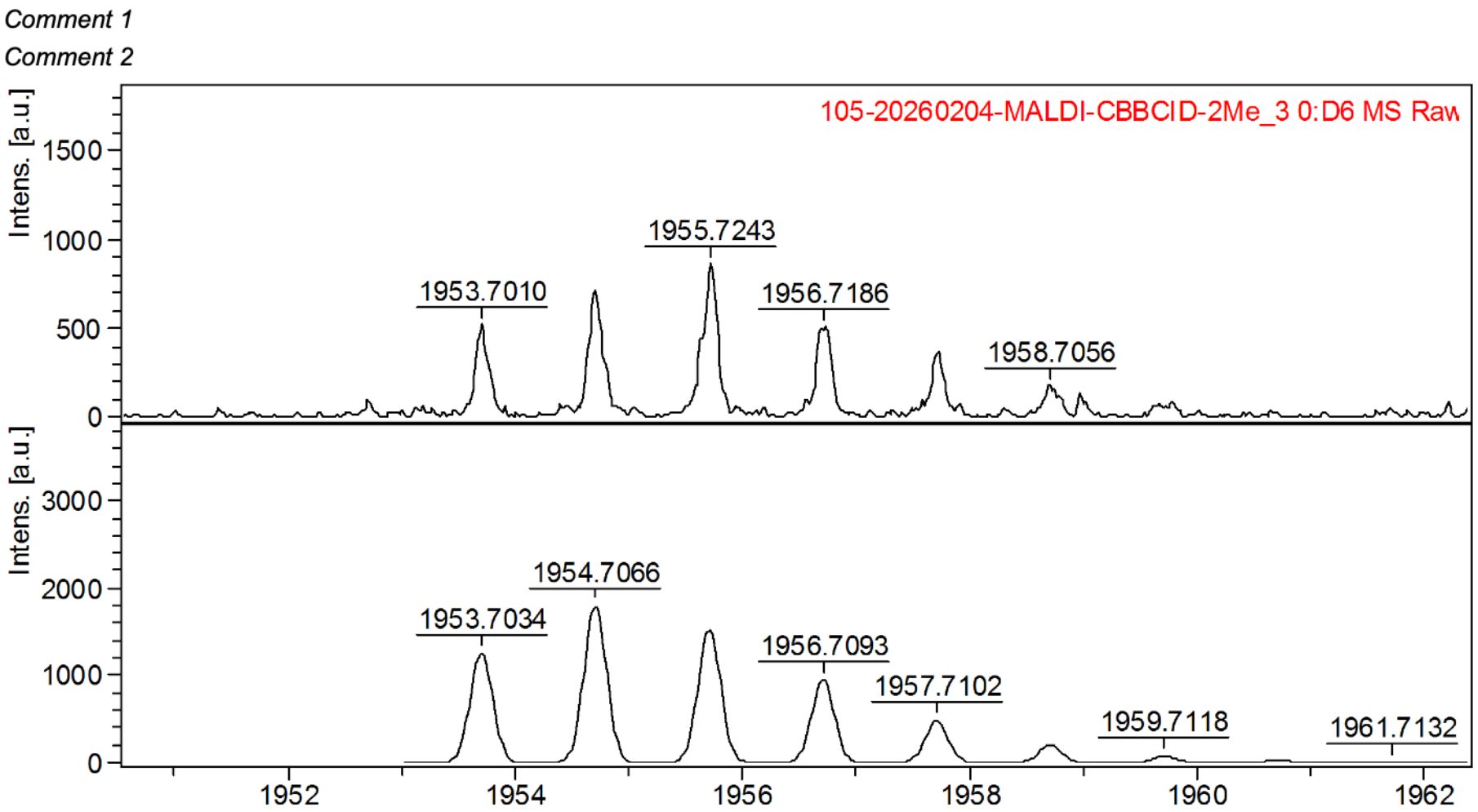

m/z	S/N	Quality Fac.	Res.	Intens.	Area
1953.7034			8652	1252	
1954.7066			8657	1800	
1955.7082			8661	1524	
1956.7093			8666	949	
1957.7102			8670	475	
1958.7110			8675	200	
1959.7118			8679	73.0	
1960.7125			8683	23.5	
1961.7132			8688	6.76	

Acquisition Parameter

Date of acquisition
Acquisition method name

Aquisition operation mode
Voltage polarity
Number of shots
Name of spectrum used for calibration
Calibration reference list used

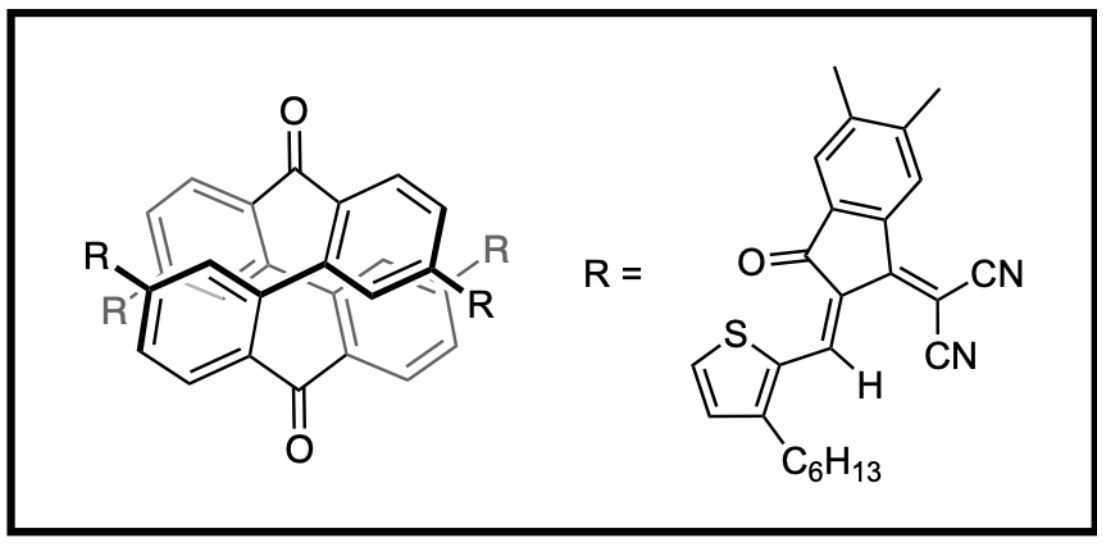

Instrument Info

User	tof-user
Instrument	FLEX-PC
Instrument type	

Figure S19. MALDI-TOF mass spectrum of **1c**.

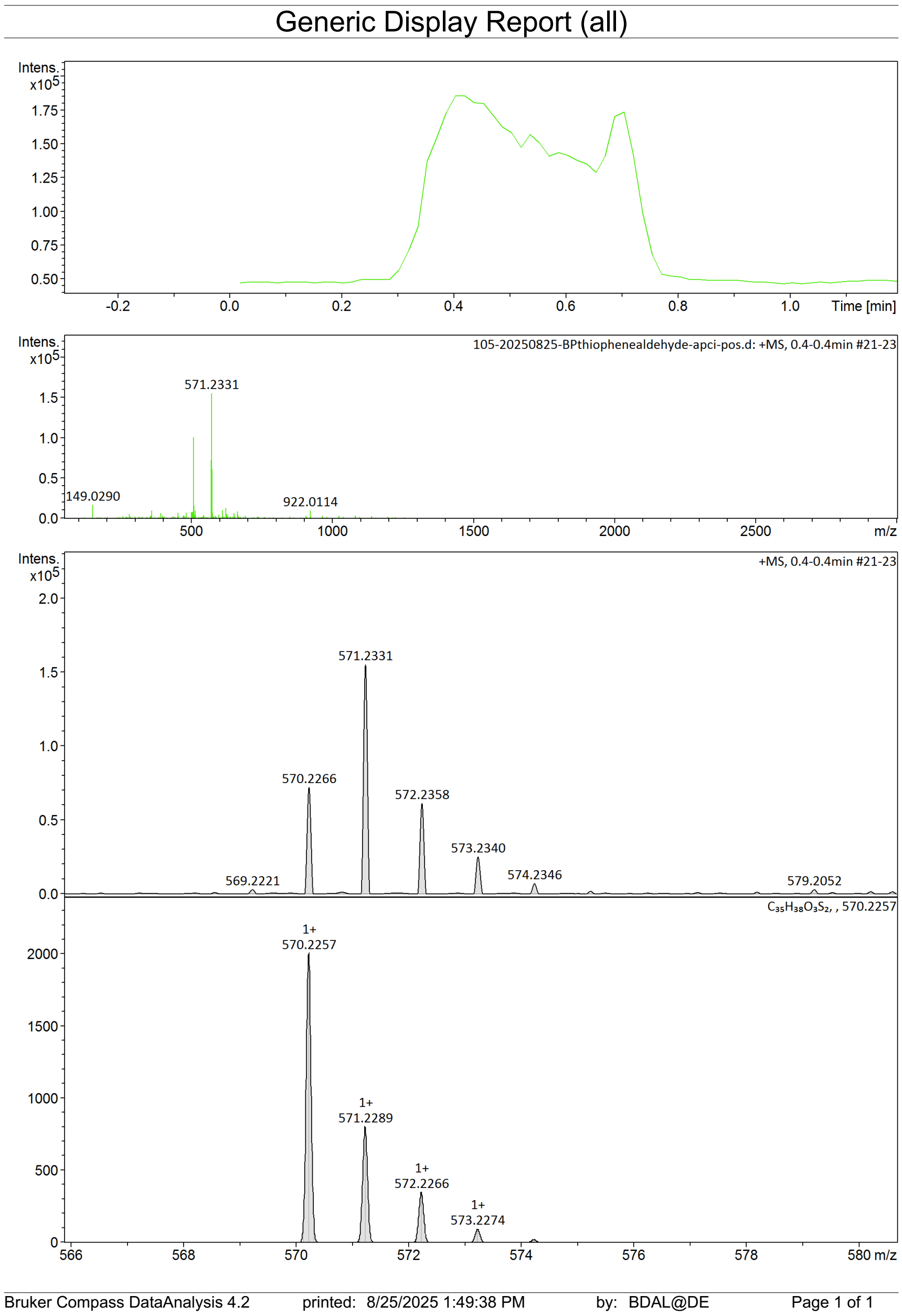

Figure S20. APCI-TOF mass spectrum of **S1**.

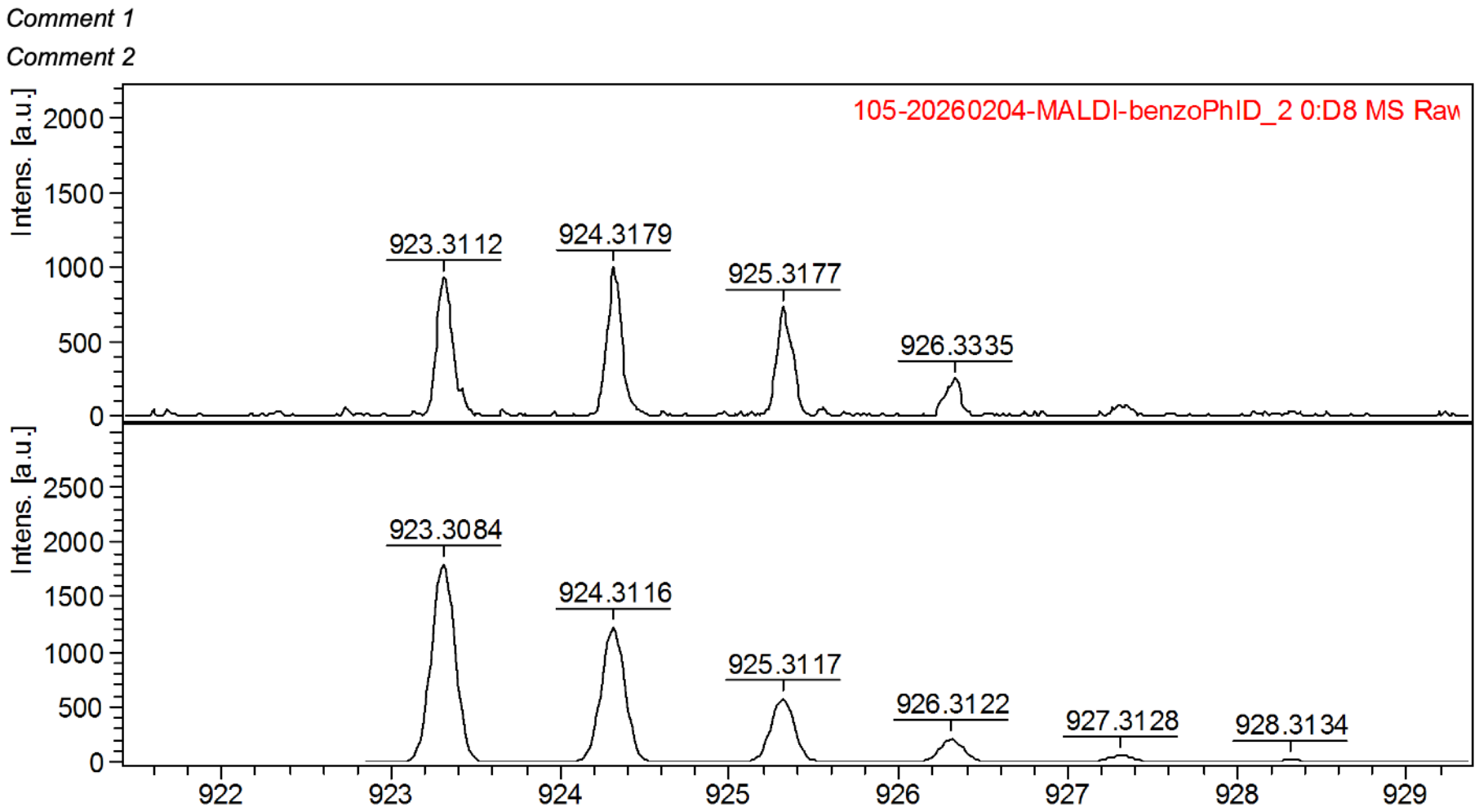

m/z	S/N	Quality Fac.	Res.	Intens.	Area
39.5757	58		2115	1846	37.2
41.5606	5		2517	167	3.88
86.4457	5		3520	175	4.87
102.4269	30		2730	1079	40.9
116.4051	104		2759	3850	169
117.4053	9		5187	339	10.3
118.3705	5		4076	201	8.58
136.0461	4		2799	150	5.56
150.2922	52		3409	1880	96.8
152.2794	17		3372	609	36.2
181.2099	11		2489	397	26.6
185.2059	8		3805	290	15.8
191.2032	9		3202	333	27.9
191.4074	4		1796	158	13.6
197.1854	5		4728	167	9.37
198.1617	4		3265	146	8.94
209.1762	9		3284	301	24.6
210.1837	8		2921	299	27.0
211.1956	5		4799	182	14.0
211.5018	6		15514	216	7.07
213.0150	46		1065	1605	333
213.9824	7		4209	263	16.7
214.0989	6		1192	222	28.8
225.1368	89		3714	3111	209
226.1526	455		3001	15843	1314
227.1689	3136		2522	109126	10831
227.9514	5		3545	163	13.7
228.1809	449		2848	15350	1336
228.3730	76		4028	2599	204
229.1843	34		3372	1147	104
229.3750	13		8648	456	23.0
239.1490	8		4346	274	17.8
241.1524	5		3732	178	16.7
242.1430	84		3953	2835	192
243.1455	11		4436	376	26.3
248.1959	5		15535	155	4.24
249.1394	11		4617	374	23.1
252.1427	4		10827	150	6.35
253.1579	5		3141	183	13.1
256.3739	83		4968	2737	177

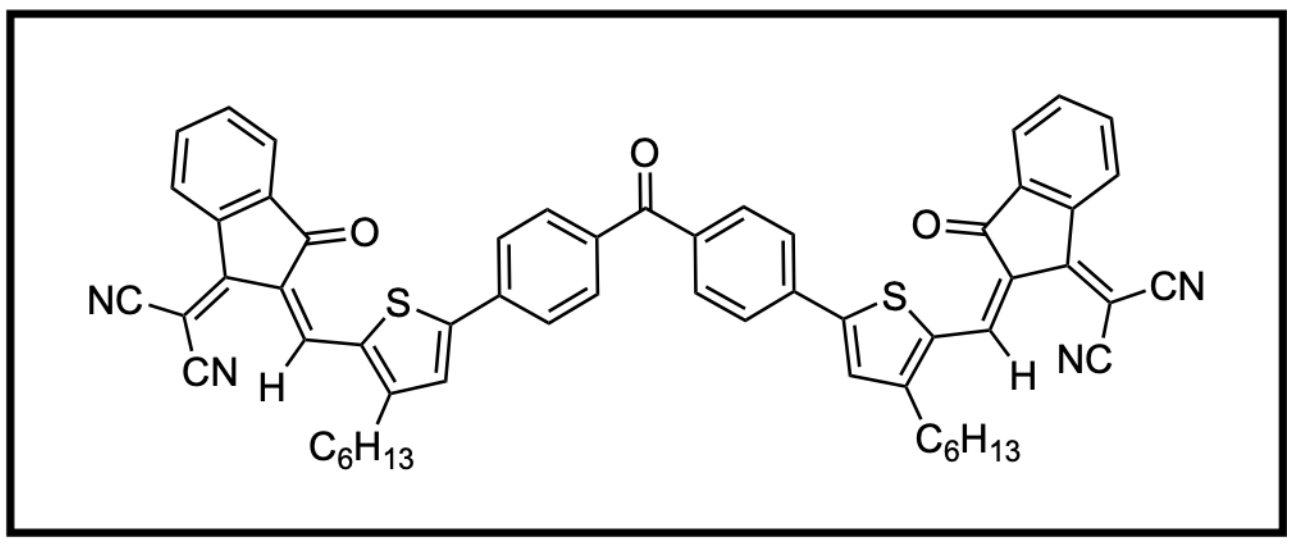

Bruker Daltonics flexAnalysis printed: 2/4/2026 10:19:05 PM

Figure S21. APCI-TOF mass spectrum of **8**.

5. Electrochemical properties

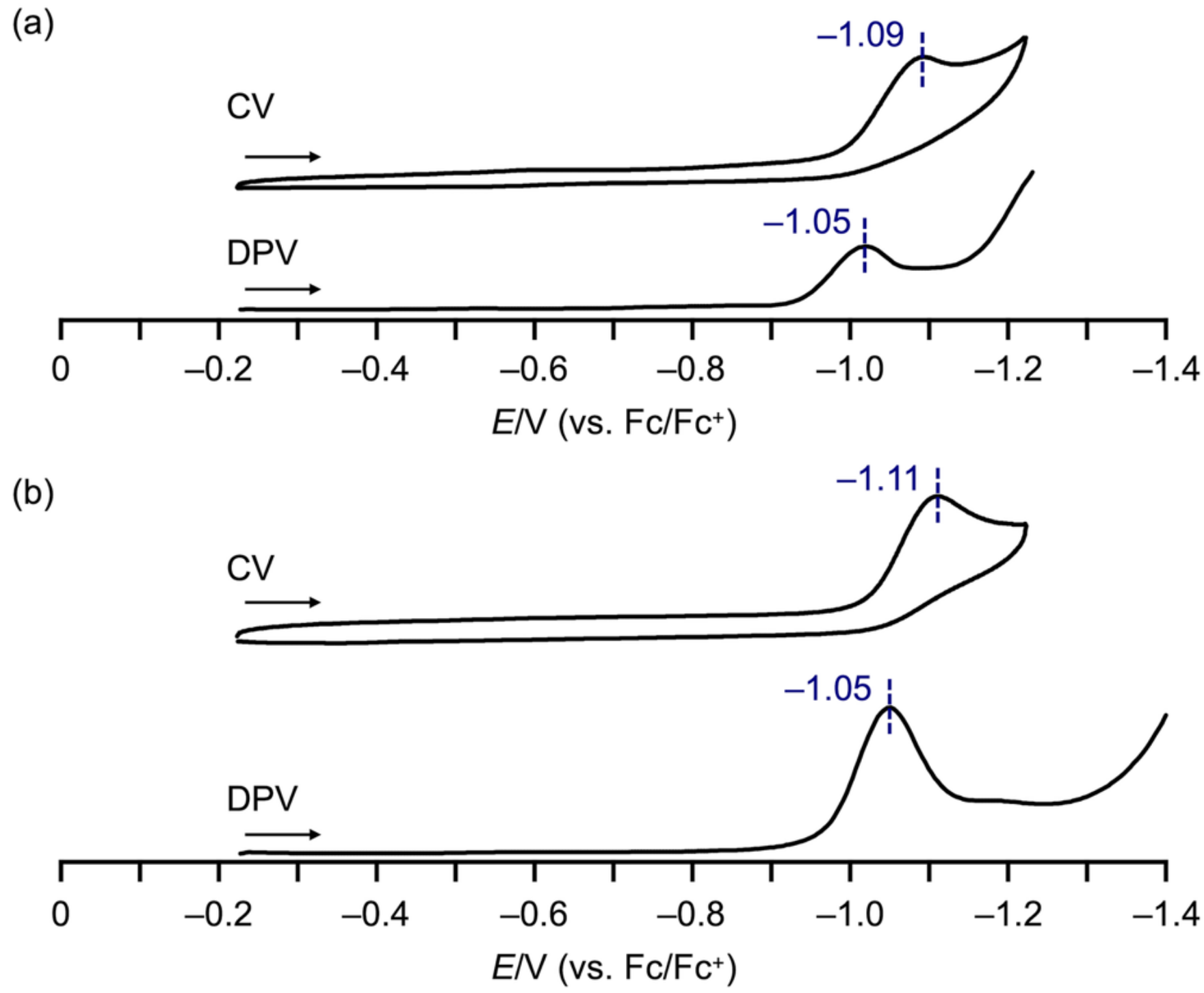

Figure S22. Cyclic and differential pulse voltammograms of (a) CBBC-TIC **1a** and (b) BP-TIC **8**.

6. Racemization

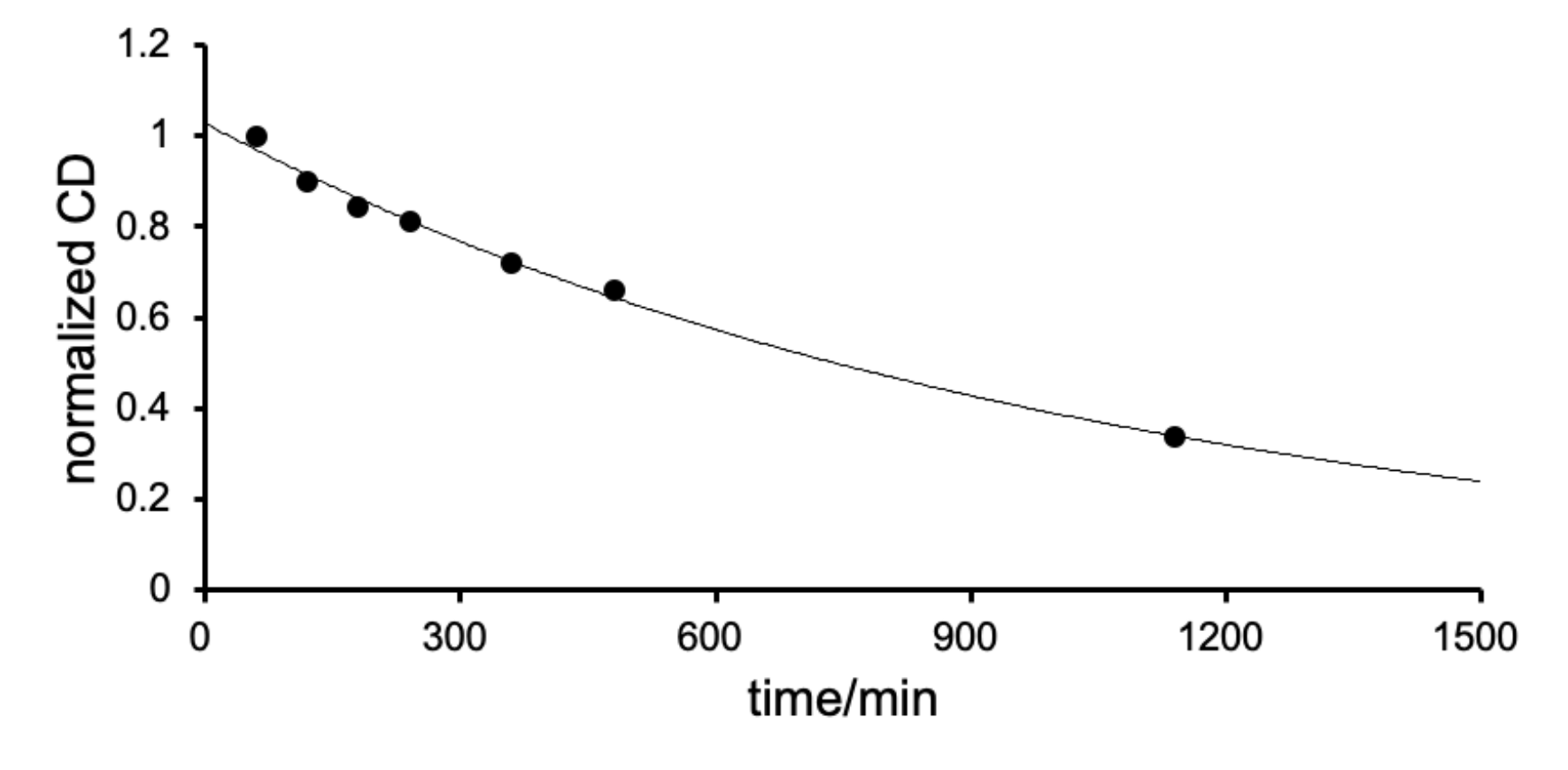

$k = 1.6 \times 10^{-5}\ \mathrm{s}^{-1}$

$t_{1/2} = 11.9$ h

$R^2 = 0.99301$

Figure S23. Plots of absolute values of CD intensities of **1a** versus time in TCE at 170 °C.

7. CD spectra

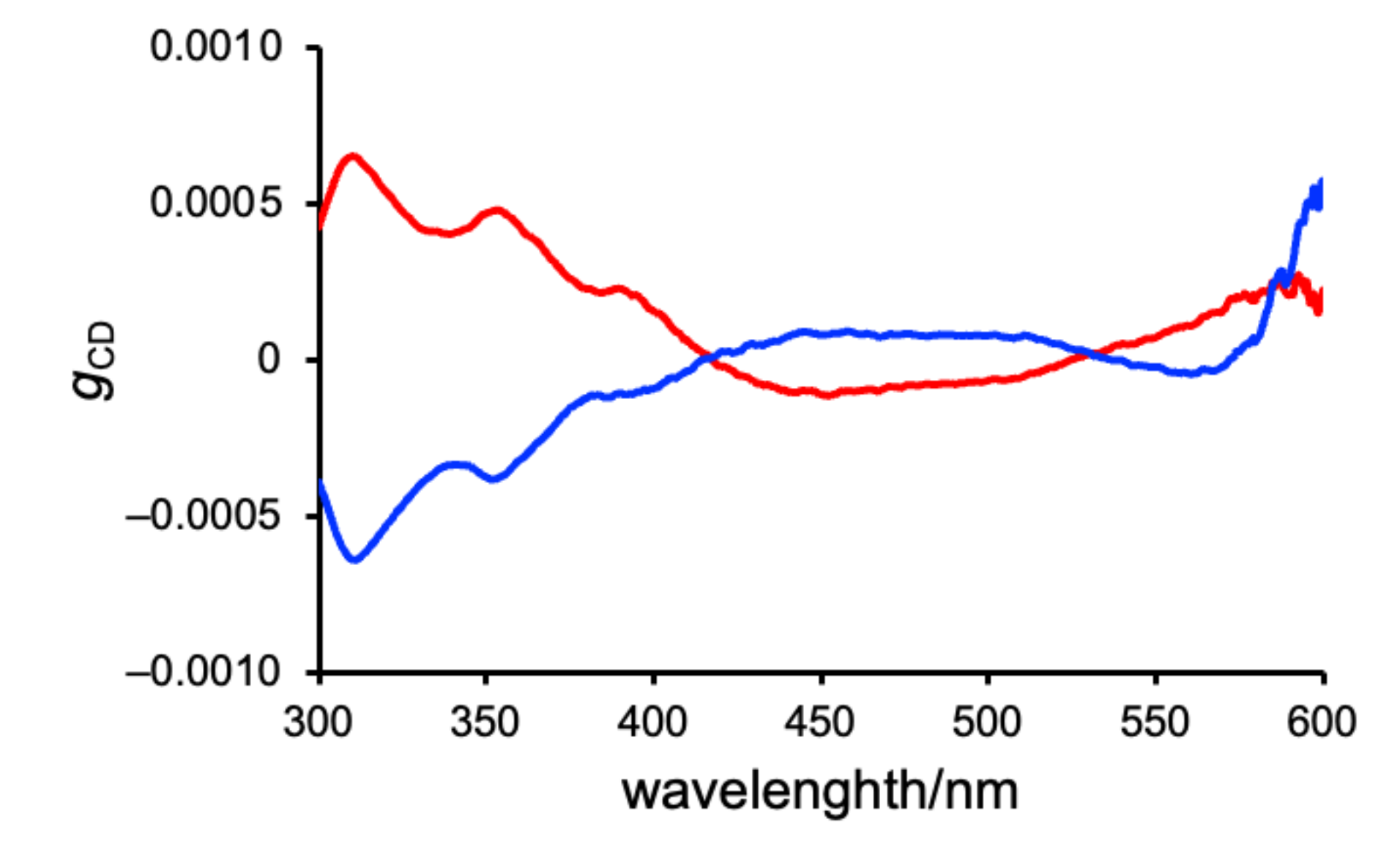

Figure S24. g_{CD} values of (*M*,*M*)-**1a** (red) and (*P*,*P*)-**1a**.

8. DFT calculations

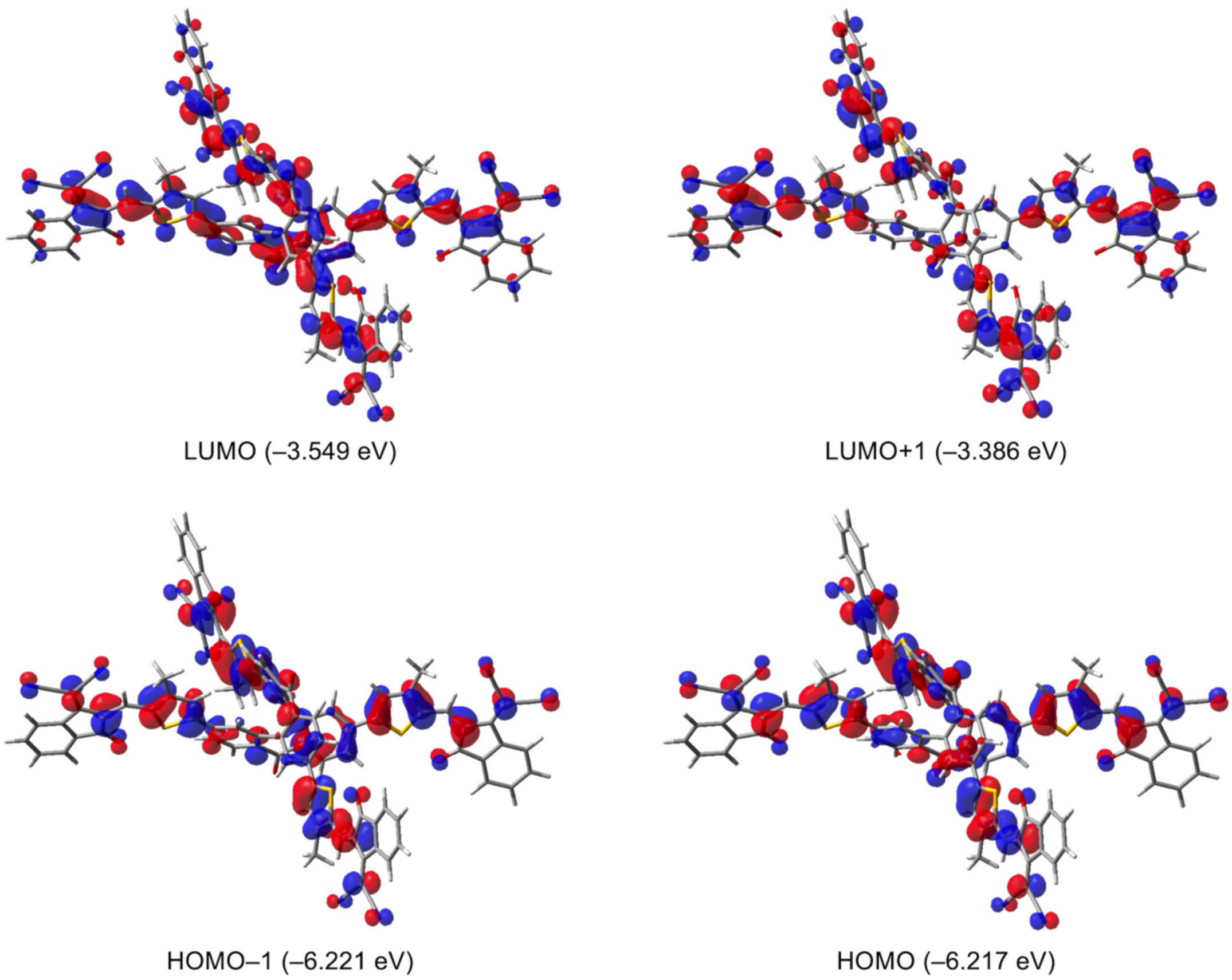

Figure S25. Calculated molecular orbitals of **1a'** (isovalue = 0.02).

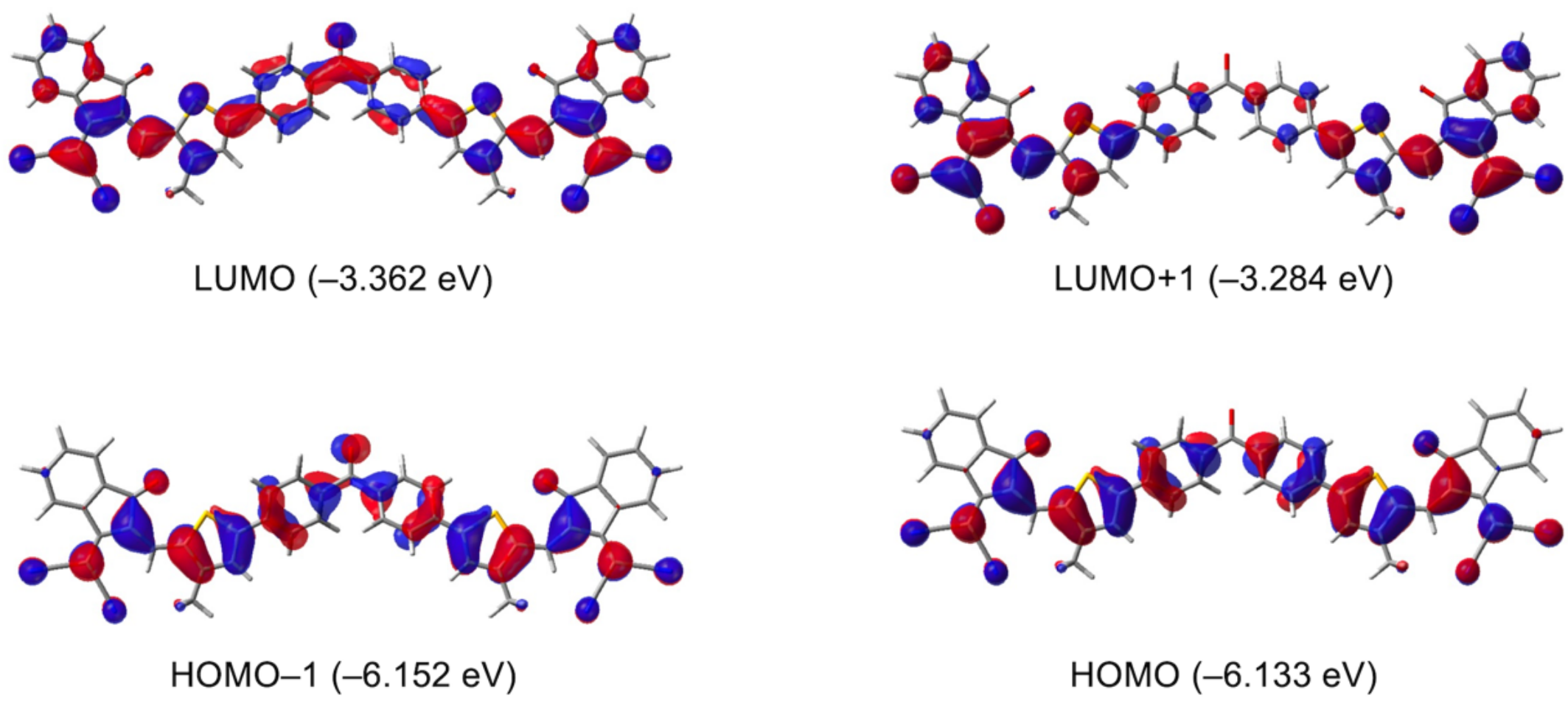

Figure S26. Calculated molecular orbitals of **8'** (isovalue = 0.02).

Table S1. Cartesian coordinate and geometry of **1a'**.

C	-0.960171	-0.892291	-1.541059	C	8.029018	-6.06767	-0.180124
C	-0.678605	-1.541671	-0.319352	C	9.178891	-6.920023	-0.517294
C	-1.665191	-2.338557	0.26728	C	8.086405	-5.796222	1.2774
H	-1.421954	-2.871596	1.18094	C	9.93232	-7.161503	0.74154
C	-2.933903	-2.510119	-0.315645	C	9.535416	-7.427895	-1.753949
C	-3.184777	-1.880715	-1.549552	C	9.281741	-6.494928	1.79609
H	-4.153671	-1.987704	-2.028103	O	7.305448	-5.125349	1.941961
C	-2.207952	-1.101212	-2.149534	C	11.095544	-7.891437	1.016334
H	-2.40061	-0.618698	-3.102035	C	8.808412	-7.193749	-2.961453
C	0.960174	0.892295	-1.541049	C	10.683648	-8.252897	-1.966002
C	0.678608	1.541658	-0.319332	C	9.745371	-6.529591	3.1043
C	1.665193	2.338539	0.267308	C	11.565987	-7.928924	2.332525
H	1.421957	2.871564	1.180977	H	11.63692	-8.423226	0.245883
C	2.933904	2.510112	-0.315616	N	8.244045	-7.026027	-3.96752
C	3.184777	1.880724	-1.549532	N	11.607628	-8.927157	-2.186509
H	4.15367	1.987722	-2.028083	C	10.90525	-7.259123	3.370098
C	2.207953	1.101227	-2.149523	H	9.206987	-5.998372	3.883334
H	2.40061	0.618727	-3.102031	H	12.467859	-8.493862	2.550178
C	0	0.000008	-2.275029	H	11.29869	-7.309834	4.381261
O	0.000003	0.000012	-3.501291	C	3.944547	3.335752	0.34814
C	0.960186	-0.892308	1.541048	C	4.01406	3.655323	1.696316
C	0.678623	-1.541672	0.319331	S	5.254002	4.052682	-0.54206
C	1.665213	-2.338545	-0.267311	C	5.110746	4.464511	2.037681
H	1.42198	-2.871573	-1.180979	H	3.306044	3.28898	2.431717
C	2.933926	-2.510109	0.315612	C	5.914224	4.784557	0.920741
C	3.184796	-1.880719	1.549527	C	7.072322	5.599626	1.054106
H	4.153691	-1.98771	2.028077	H	7.203393	5.903619	2.085192
C	2.207967	-1.10123	2.149519	C	8.028976	6.067711	0.180122
H	2.400622	-0.618729	3.102028	C	9.17884	6.92007	0.517304
C	-0.960176	0.892261	1.541062	C	8.086366	5.796283	-1.277406
C	-0.678618	1.541644	0.319354	C	9.932265	7.161579	-0.741527
C	-1.665213	2.338521	-0.267275	C	9.53536	7.427918	1.75397
H	-1.421983	2.871563	-1.180936	C	9.281696	6.495007	-1.796085
C	-2.933926	2.510071	0.315652	O	7.305424	5.1254	-1.941974
C	-3.184791	1.880663	1.549559	C	11.095479	7.891533	-1.016311
H	-4.153685	1.987643	2.028112	C	8.808357	7.193735	2.961467
C	-2.207957	1.101169	2.149539	C	10.683583	8.252928	1.966043
H	-2.400609	0.618653	3.10204	C	9.745328	6.529692	-3.104294
C	0.000007	-0.000027	2.27503	C	11.565923	7.929042	-2.332501
O	0.000004	-0.00004	3.501292	H	11.636847	8.423321	-0.245854
C	-3.944578	3.335709	-0.348091	N	8.243999	7.025952	3.967528
C	-4.014098	3.655297	-1.696264	N	11.607555	8.927193	2.186568
S	-5.254041	4.052612	0.542121	C	10.905197	7.259243	-3.370083
C	-5.11079	4.464484	-2.037614	H	9.206952	5.998474	-3.883335
H	-3.306082	3.28897	-2.431672	H	12.467787	8.493996	-2.550147
C	-5.914266	4.784513	-0.920667	H	11.298637	7.309971	-4.381245
C	-7.07236	5.59959	-1.054023	C	-3.944546	-3.335766	0.348102
H	-7.203424	5.903594	-2.085106	C	-4.014057	-3.655356	1.696275
C	-8.029016	6.067673	-0.18004	S	-5.254007	-4.05268	-0.542104
C	-9.932313	7.161533	0.741607	C	-5.110744	-4.464547	2.037632
C	-9.281757	6.494945	1.796162	H	-3.306039	-3.289025	2.431679
C	-11.095531	7.891482	1.016387	C	-5.914226	-4.784575	0.920688
C	-9.745407	6.529608	3.104365	C	-7.072325	-5.599644	1.054041
C	-11.565993	7.928969	2.33257	H	-7.203399	-5.903654	2.085122
H	-11.636889	8.423281	0.245929	C	-8.028975	-6.06771	0.180045
C	-10.90528	7.259154	3.37015	C	-9.178845	-6.920071	0.517202
H	-9.207041	5.998378	3.883405	C	-8.086354	-5.796254	-1.277478
H	-12.467861	8.493918	2.550214	C	-9.932266	-7.161548	-0.741638
H	-11.298733	7.309866	4.381308	C	-9.535374	-7.427944	1.753856
C	-9.178874	6.920045	-0.517219	C	-9.281687	-6.494956	-1.796179
C	-8.086421	5.79623	1.277485	O	-7.305407	-5.125356	-1.942026
O	-7.305489	5.125338	1.942056	C	-11.095483	-7.891488	-1.016445
C	-9.535383	7.427932	-1.753873	C	-8.808377	-7.193789	2.961362
C	-8.808376	7.19379	-2.961375	C	-10.683603	-8.252951	1.965906
N	-8.244054	7.02602	-3.967459	C	-9.745313	-6.529608	-3.10439
C	-10.683604	8.252951	-1.965921	C	-11.565921	-7.928964	-2.332637
N	-11.607576	8.927225	-2.186419	H	-11.636858	-8.42329	-0.246002
C	3.944574	-3.335741	-0.348146	N	-8.244023	-7.026032	3.96743
C	4.014088	-3.655312	-1.696323	N	-11.60758	-8.927215	2.186416
S	5.254037	-4.052661	0.542053	C	-10.905186	-7.259144	-3.370201
C	5.11078	-4.464493	-2.037689	H	-9.20693	-5.998376	-3.883417
H	3.30607	-3.288974	-2.431723	H	-12.467788	-8.493906	-2.550301
C	5.914261	-4.784533	-0.920749	H	-11.298622	-7.309848	-4.381366
C	7.072363	-5.599597	-1.054113	C	-5.380346	4.915132	-3.449456
H	7.203437	-5.903591	-2.085198	H	-6.351545	4.564148	-3.815998

H	-5.387604	6.007199	-3.539288	H	6.351531	-4.564123	-3.816078
H	-4.610198	4.530301	-4.123438	H	5.387596	-6.007181	-3.539391
C	5.380298	4.915138	3.449531	H	4.610183	-4.530276	-4.123513
H	6.351501	4.564158	3.816067	C	-5.380293	-4.915193	3.449476
H	5.387545	6.007203	3.539383	H	-6.351495	-4.564217	3.81602
H	4.610154	4.530287	4.123506	H	-5.387541	-6.007259	3.539312
C	5.380334	-4.915116	-3.449539	H	-4.610147	-4.530352	4.123454

Negative frequency = zero

Sum of electronic and thermal free energies = –6244.287466 Hartree

Table S2. Cartesian coordinate and geometry of **1b'**.

C	0.960041	-0.892238	1.541371	C	-9.186115	-6.910559	0.521223
C	0.678647	-1.541253	0.319404	C	-8.088931	-5.794323	-1.275864
C	1.665408	-2.337751	-0.26746	C	-9.934716	-7.15526	-0.736187
H	1.422294	-2.87051	-1.18132	C	-9.547455	-7.410614	1.760184
C	2.934273	-2.509156	0.315192	C	-9.285041	-6.493829	-1.79255
C	3.18491	-1.880172	1.549362	O	-7.306329	-5.1268	-1.941947
H	4.153922	-1.986997	2.027718	C	-11.0958	-7.886723	-1.020695
C	2.207884	-1.101217	2.1497	C	-8.82492	-7.171259	2.969055
H	2.400487	-0.618869	3.102302	C	-10.699089	-8.231162	1.970224
C	-0.959994	0.892276	1.541354	C	-9.744889	-6.533835	-3.100963
C	-0.678578	1.54128	0.319386	C	-11.55685	-7.925899	-2.336902
C	-1.665327	2.337779	-0.267498	H	-11.641644	-8.421517	-0.255227
H	-1.422197	2.870531	-1.181359	N	-8.264298	-6.999132	3.976568
C	-2.934199	2.509195	0.315134	N	-11.626883	-8.901579	2.186748
C	-3.184857	1.880225	1.549306	C	-10.905845	-7.259919	-3.392929
H	-4.153874	1.98706	2.027648	H	-9.198921	-6.004436	-3.877137
C	-2.207844	1.101269	2.149664	H	-12.456602	-8.496815	-2.553681
H	-2.400463	0.618929	3.102268	C	-3.945264	3.333841	-0.349222
C	0.000012	0.000013	2.2754	C	-4.01513	3.652701	-1.697304
O	0.000035	0.000048	3.501756	S	-5.255563	4.050554	0.540356
C	-0.959959	-0.892252	-1.541307	C	-5.112791	4.460905	-2.039128
C	-0.678559	-1.54126	-0.319338	H	-3.306856	3.286571	-2.432595
C	-1.665314	-2.337765	0.267528	C	-5.91637	4.780734	-0.922798
H	-1.422195	-2.870519	1.18139	C	-7.076573	5.593935	-1.056587
C	-2.934177	-2.509184	-0.315123	H	-7.208628	5.896661	-2.087967
C	-3.18482	-1.880206	-1.549295	C	-8.033178	6.060978	-0.183097
H	-4.153829	-1.987041	-2.027652	C	-9.186131	6.910516	-0.521294
C	-2.207801	-1.101243	-2.149635	C	-8.088909	5.794343	1.275808
H	-2.400408	-0.6189	-3.10224	C	-9.934775	7.155159	0.736107
C	0.960061	0.892278	-1.541297	C	-9.547423	7.410559	-1.760273
C	0.67864	1.541285	-0.319331	C	-9.285088	6.49375	1.792468
C	1.665381	2.337796	0.267549	O	-7.306363	5.126733	1.94187
H	1.422246	2.87055	1.181407	C	-11.095951	7.886503	1.020598
C	2.934252	2.509221	-0.315084	C	-8.824806	7.17119	-2.969093
C	3.184915	1.880247	-1.549253	C	-10.699054	8.231088	-1.970405
H	4.153932	1.987088	-2.027596	C	-9.744999	6.533664	3.100872
C	2.207909	1.101279	-2.149608	C	-11.557052	7.92559	2.336779
H	2.400533	0.618937	-3.102209	H	-11.641845	8.421228	0.25512
C	0.000061	0.000006	-2.27534	N	-8.26407	6.999025	-3.976537
O	0.000043	0.000033	-3.501696	N	-11.626829	8.901491	-2.18705
C	3.94531	3.33388	0.349267	C	-10.906002	7.259654	3.392821
C	4.015175	3.65275	1.697346	H	-9.199038	6.004257	3.877043
S	5.255617	4.050575	-0.540313	H	-12.456894	8.496375	2.553536
C	5.112822	4.460976	2.039163	C	3.945353	-3.333803	-0.349139
H	3.306904	3.286619	2.43264	C	4.015243	-3.652677	-1.697217
C	5.916389	4.780812	0.922827	S	5.255664	-4.050465	0.540461
C	7.076565	5.594053	1.05658	C	5.112903	-4.460894	-2.03901
H	7.208641	5.89681	2.087949	H	3.30698	-3.28656	-2.432524
C	8.033122	6.061091	0.183039	C	5.916457	-4.780724	-0.922662
C	9.934594	7.15536	-0.736313	C	7.07663	-5.593972	-1.056428
C	9.28485	6.493917	-1.792625	H	7.208673	-5.896713	-2.087804
C	11.095677	7.886803	-1.020914	C	8.033223	-6.061046	-0.182936
C	9.744615	6.533901	-3.101074	C	9.18615	-6.910626	-0.52113
C	11.556638	7.925954	-2.337146	C	8.088971	-5.794404	1.275968
H	11.641597	8.421578	-0.25549	C	9.934767	-7.15532	0.736273
C	10.905539	7.259982	-3.393129	C	9.547464	-7.410685	-1.760097
H	9.19861	6.004472	-3.8772	C	9.285104	-6.493883	1.792638
H	12.456401	8.496831	-2.553988	O	7.306414	-5.126823	1.942045
C	9.186073	6.910663	0.521153	C	11.095871	-7.88676	1.02077
C	8.088788	5.794391	-1.275854	C	8.824909	-7.171309	-2.968952
O	7.306141	5.126853	-1.941868	C	10.69908	-8.23125	-1.970166
C	9.547427	7.410713	1.760112	C	9.744982	-6.53386	3.101044
C	8.824896	7.1713	2.968974	C	11.556948	-7.925908	2.336965
N	8.264243	6.999062	3.976451	H	11.64172	-8.42154	0.255295
C	10.699034	8.231292	1.970183	N	8.264254	-6.999163	-3.976444
N	11.626784	8.901736	2.186805	N	11.626855	-8.901682	-2.186723
C	-3.945248	-3.333841	0.34921	C	10.905945	-7.259929	3.392999
C	-4.01512	-3.652733	1.697284	H	9.199029	-6.004449	3.87722
S	-5.25555	-4.050521	-0.540389	H	12.456724	-8.496792	2.553732
C	-5.112782	-4.460945	2.039083	C	5.382487	4.910911	3.451316
H	-3.306848	-3.286624	2.432587	H	6.354923	4.562481	3.816909
C	-5.916358	-4.780749	0.922743	H	5.386761	6.002944	3.542071
C	-7.076552	-5.593964	1.056518	H	4.613839	4.523448	4.125551
H	-7.208591	-5.896709	2.087893	C	-5.382472	4.910812	-3.451287
C	-8.033169	-6.061002	0.183032	H	-6.3549	4.562353	-3.81687

H	-5.386772	6.002843	-3.542058
H	-4.613817	4.523357	-4.125518
C	-5.382463	-4.910893	3.451228
H	-6.3549	-4.562461	3.816814
H	-5.386747	-6.002928	3.541968
H	-4.613818	-4.523445	4.125474
C	5.382594	-4.910838	-3.451155
H	6.355037	-4.562415	-3.81673
H	5.386866	-6.002872	-3.541901
H	4.61396	-4.523378	-4.125407
C	-11.460809	7.321488	4.794545
H	-12.375232	6.720557	4.882026
H	-10.742367	6.940574	5.526165
H	-11.72259	8.347979	5.075119
C	11.460234	7.321791	-4.794898
H	12.373897	6.719753	-4.882769
H	10.741239	6.942069	-5.526597
H	11.72325	8.348076	-5.075042
C	11.460797	-7.321641	4.794709
H	12.373712	-6.71849	4.882768
H	10.741386	-6.943086	5.526606
H	11.725106	-8.347706	5.074421
C	-11.460706	-7.321563	-4.794638
H	-12.372828	-6.717252	-4.883008
H	-10.740782	-6.944242	-5.526671
H	-11.726334	-8.347402	-5.073904

Negative frequency = zero

Sum of electronic and thermal free energies = –6401.468637 Hartree

Table S3. Cartesian coordinate and geometry of **1c'**.

C	0.959839	-0.892316	1.54149	C	-8.088287	-5.795146	-1.27862
C	0.678547	-1.540914	0.319258	C	-9.933225	-7.155627	-0.739194
C	1.665343	-2.337278	-0.267745	C	-9.545633	-7.413811	1.758255
H	1.422304	-2.869738	-1.181806	C	-9.282246	-6.494849	-1.792846
C	2.934184	-2.508993	0.314894	O	-7.304731	-5.126478	-1.94294
C	3.184671	-1.880419	1.549317	C	-11.094495	-7.882916	-1.022226
H	4.153671	-1.987438	2.027653	C	-8.822476	-7.17547	2.966986
C	2.207637	-1.101622	2.149809	C	-10.69597	-8.235877	1.969723
H	2.400209	-0.619481	3.102526	C	-9.753137	-6.541363	-3.096395
C	-0.959892	0.892192	1.541635	C	-11.585324	-7.941358	-2.333655
C	-0.678617	1.540995	0.31951	H	-11.634989	-8.410797	-0.247735
C	-1.665428	2.337443	-0.267356	N	-8.262032	-7.004815	3.974886
H	-1.422405	2.870054	-1.181334	N	-11.622261	-8.907649	2.188776
C	-2.934268	2.509039	0.315319	C	-10.913411	-7.266558	-3.38579
C	-3.184741	1.880253	1.549636	H	-9.214344	-6.013502	-3.878641
H	-4.153742	1.987177	2.027993	C	-3.945453	3.333571	-0.348955
C	-2.207691	1.101372	2.149995	C	-4.016189	3.651795	-1.696973
H	-2.400249	0.619074	3.102636	S	-5.255346	4.051028	0.541054
C	-0.000006	-0.0001	2.275619	C	-5.114151	4.460111	-2.038354
O	-0.00007	-0.000262	3.502057	H	-3.308528	3.285203	-2.432657
C	-0.959922	-0.892064	-1.541693	C	-5.916803	4.780619	-0.921879
C	-0.67864	-1.540858	-0.319562	C	-7.077186	5.594526	-1.055313
C	-1.665447	-2.337307	0.267309	H	-7.209606	5.896654	-2.086809
H	-1.422418	-2.869917	1.181287	C	-8.03272	6.062116	-0.181601
C	-2.934286	-2.508915	-0.315362	C	-9.185503	6.912205	-0.519313
C	-3.184765	-1.88014	-1.549684	C	-8.088281	5.795162	1.278611
H	-4.153765	-1.987074	-2.028038	C	-9.933248	7.155626	0.739242
C	-2.20772	-1.101258	-2.15005	C	-9.545692	7.413906	-1.758195
H	-2.400283	-0.618965	-3.102692	C	-9.282249	6.494828	1.792869
C	0.959833	0.892418	-1.541551	O	-7.304707	5.126488	1.942903
C	0.678564	1.541033	-0.319323	C	-11.094528	7.882886	1.022304
C	1.665386	2.337371	0.267672	C	-8.822556	7.17563	-2.966952
H	1.422366	2.869841	1.181732	C	-10.696049	8.235958	-1.969606
C	2.934231	2.509042	-0.314971	C	-9.75313	6.541294	3.096423
C	3.184697	1.880449	-1.549389	C	-11.585349	7.94128	2.333739
H	4.153698	1.987436	-2.027729	H	-11.635037	8.410781	0.247833
C	2.207637	1.101678	-2.149873	N	-8.262139	7.005038	-3.974877
H	2.40019	0.619527	-3.10259	N	-11.622361	8.907721	-2.188598
C	-0.000058	0.000251	-2.275679	C	-10.913416	7.266461	3.385848
O	0.000021	0.000263	-3.502116	H	-9.214323	6.013419	3.878649
C	3.945425	3.333456	0.349434	C	3.945352	-3.333433	-0.34952
C	4.016135	3.651511	1.697494	C	4.016056	-3.651469	-1.697584
S	5.255366	4.050984	-0.54045	S	5.255264	-4.05102	0.540357
C	5.11411	4.459755	2.039003	C	5.114005	-4.459744	-2.039104
H	3.308443	3.284854	2.433114	H	3.308379	-3.284774	-2.433201
C	5.9168	4.780378	0.922591	C	5.916681	-4.780413	-0.922692
C	7.077204	5.594232	1.056174	C	7.077059	-5.594303	-1.056266
H	7.209586	5.89622	2.087717	H	7.209453	-5.896291	-2.087806
C	8.032801	6.061914	0.182579	C	8.032616	-6.062009	-0.182641
C	9.933429	7.155506	-0.737961	C	9.185401	-6.91204	-0.520496
C	9.282474	6.494904	-1.791738	C	8.088202	-5.795264	1.277608
C	11.094761	7.882761	-1.02083	C	9.933181	-7.155618	0.738008
C	9.753445	6.541558	-3.095253	C	9.545563	-7.413576	-1.759453
C	11.585672	7.941344	-2.332223	C	9.282195	-6.494978	1.791742
H	11.635243	8.410507	-0.24624	O	7.304634	-5.126692	1.942011
C	10.91378	7.266721	-3.384485	C	11.094486	-7.88289	1.020941
H	9.214666	6.013831	-3.877599	C	8.822392	-7.175155	-2.96816
C	9.185588	6.911907	0.520504	C	10.695925	-8.235586	-1.970999
C	8.088444	5.795201	-1.277674	C	9.753109	-6.541613	3.095278
O	7.304895	5.126659	-1.942128	C	11.585341	-7.941453	2.332355
C	9.545683	7.413382	1.759504	H	11.634988	-8.410664	0.246383
C	8.822418	7.174905	2.968144	N	8.261951	-7.004449	-3.976053
N	8.261883	7.004092	3.975967	N	11.62224	-8.907309	-2.190105
C	10.696041	8.235366	1.971176	C	10.913418	-7.266793	3.384574
N	11.622343	8.907066	2.190401	H	9.214309	-6.013858	3.877589
C	-3.945465	-3.333451	0.348917	C	5.384738	4.908618	3.451379
C	-4.016203	-3.651658	1.696939	H	6.356521	4.558035	3.816731
S	-5.255344	-4.050935	-0.541089	H	5.391256	6.000576	3.542821
C	-5.114158	-4.459983	2.038325	H	4.615401	4.522271	4.125506
H	-3.308551	-3.285048	2.432621	C	-5.384805	4.909148	-3.450671
C	-5.9168	-4.780515	0.92185	H	-6.356609	4.558637	-3.816036
C	-7.077174	-5.594434	1.055296	H	-5.391292	6.001116	-3.541986
H	-7.209589	-5.896543	2.086799	H	-4.615499	4.522855	-4.124864
C	-8.032706	-6.062056	0.1816	C	-5.384815	-4.909005	3.450645
C	-9.185473	-6.912152	0.519348	H	-6.356624	-4.558497	3.816

H	-5.391294	-6.000972	3.541974
H	-4.615516	-4.522697	4.124838
C	5.38462	-4.90859	-3.451488
H	6.356416	-4.558035	-3.816831
H	5.391098	-6.000546	-3.542952
H	4.615298	-4.522201	-4.125609
C	-11.437459	7.326355	4.798752
H	-12.455349	6.922038	4.868642
H	-10.801107	6.75388	5.479169
H	-11.483251	8.35872	5.168304
C	11.437919	7.32682	-4.797346
H	12.45579	6.922455	-4.867237
H	10.801578	6.754496	-5.4779
H	11.483796	8.359245	-5.166722
C	11.437496	-7.326871	4.797458
H	12.455371	-6.92252	4.867384
H	10.801136	-6.754522	5.477973
H	11.483342	-8.359288	5.166858
C	-11.437463	-7.326504	-4.798689
H	-12.455353	-6.922187	-4.868586
H	-10.801116	-6.754055	-5.479131
H	-11.483262	-8.358883	-5.168202
C	12.840219	8.732009	-2.61469
H	13.627739	8.097633	-3.041159
H	12.654828	9.534302	-3.340248
H	13.233782	9.18841	-1.702689
C	-12.839845	8.731952	2.616417
H	-13.627366	8.097536	3.042825
H	-12.654376	9.534113	3.3421
H	-13.233445	9.188524	1.704517
C	-12.839806	-8.732065	-2.6163
H	-13.627337	-8.097684	-3.042742
H	-12.65432	-9.534258	-3.341944
H	-13.2334	-9.188601	-1.70438
C	12.83986	-8.732138	2.614893
H	13.627374	-8.09777	3.041384
H	12.65442	-9.534414	3.340457
H	13.233454	-9.188564	1.702917

Negative frequency = zero

Sum of electronic and thermal free energies = –6558.630772 Hartree

Table S4. Cartesian coordinate and geometry of **8'**.

C	1.303357	1.438811	0.012662	C	-12.092795	-1.816494	-0.145015
C	1.466106	0.170575	0.592249	C	-11.372348	-3.04304	-0.278318
C	2.7179	-0.433256	0.63635	N	-10.822146	-4.064875	-0.388227
H	2.827432	-1.395019	1.127477	C	-13.507998	-2.019028	-0.145088
C	3.84765	0.201613	0.086036	N	-14.653191	-2.232651	-0.149782
C	3.67891	1.479527	-0.48589	C	5.155481	-0.456215	0.119591
H	4.530152	1.988365	-0.928845	C	5.407687	-1.816695	0.225061
C	2.433925	2.090355	-0.506771	S	6.631705	0.456189	0.027982
H	2.311329	3.081033	-0.93183	C	6.772281	-2.148988	0.22553
C	-1.303364	1.438808	-0.012591	H	4.623142	-2.564216	0.268972
C	-1.466104	0.170569	-0.592175	C	7.605244	-1.012444	0.120581
C	-2.717896	-0.433265	-0.636288	C	9.021452	-1.134656	0.110838
H	-2.827419	-1.395029	-1.127412	H	9.311562	-2.174479	0.196565
C	-3.847653	0.201604	-0.085989	C	10.067653	-0.241922	0.01886
C	-3.678923	1.479521	0.485934	C	11.499999	-0.571198	0.029715
H	-4.530171	1.988358	0.928877	C	9.948234	1.230342	-0.116736
C	-2.433939	2.090351	0.506827	C	12.247241	0.707417	-0.105357
H	-2.311351	3.081031	0.931883	C	12.092779	-1.816503	0.144978
C	-0.000004	2.180065	0.000042	C	11.324712	1.766173	-0.190696
O	-0.000005	3.406331	0.000028	O	8.919382	1.894319	-0.162168
C	-5.155482	-0.456228	-0.119556	C	13.616681	0.996028	-0.156045
C	-5.407685	-1.816709	-0.225025	C	11.372312	-3.043036	0.278293
S	-6.631709	0.456173	-0.027965	C	13.507978	-2.01906	0.14504
C	-6.772277	-2.149004	-0.225509	C	11.715065	3.091844	-0.323104
H	-4.623137	-2.564227	-0.268926	C	14.014348	2.329929	-0.289658
C	-7.605244	-1.012461	-0.120572	H	14.370495	0.222983	-0.095551
C	-9.021452	-1.134674	-0.110845	N	10.822084	-4.064856	0.388211
H	-9.311563	-2.174495	-0.196573	N	14.653168	-2.232705	0.149726
C	-10.067649	-0.241934	-0.018881	C	13.081728	3.37149	-0.372679
C	-12.247229	0.707427	0.105312	H	10.963763	3.87336	-0.384378
C	-11.324691	1.766174	0.190658	H	15.075576	2.55832	-0.329454
C	-13.616667	0.99605	0.155988	H	13.425903	4.396583	-0.475634
C	-11.715033	3.091849	0.32306	C	-7.263218	-3.570217	-0.315961
C	-14.014323	2.329955	0.289594	H	-7.8532	-3.858986	0.56119
H	-14.370487	0.223012	0.095489	H	-7.903404	-3.731027	-1.190596
C	-13.081694	3.371507	0.372622	H	-6.417332	-4.259091	-0.390491
H	-10.963725	3.873358	0.384339	C	7.263226	-3.570199	0.31598
H	-15.075549	2.558356	0.32938	H	7.853194	-3.85897	-0.56118
H	-13.425861	4.396604	0.475571	H	7.903426	-3.731006	1.190605
C	-11.499999	-0.571196	-0.02975	H	6.417342	-4.259075	0.390525
C	-9.948218	1.23033	0.116711	H	0.61852	-0.333424	1.045205
O	-8.919359	1.894297	0.162151	H	-0.618512	-0.333429	-1.04512

Negative frequency = zero

Sum of electronic and thermal free energies = –3123.341548 Hartree

9. Cost of synthesis

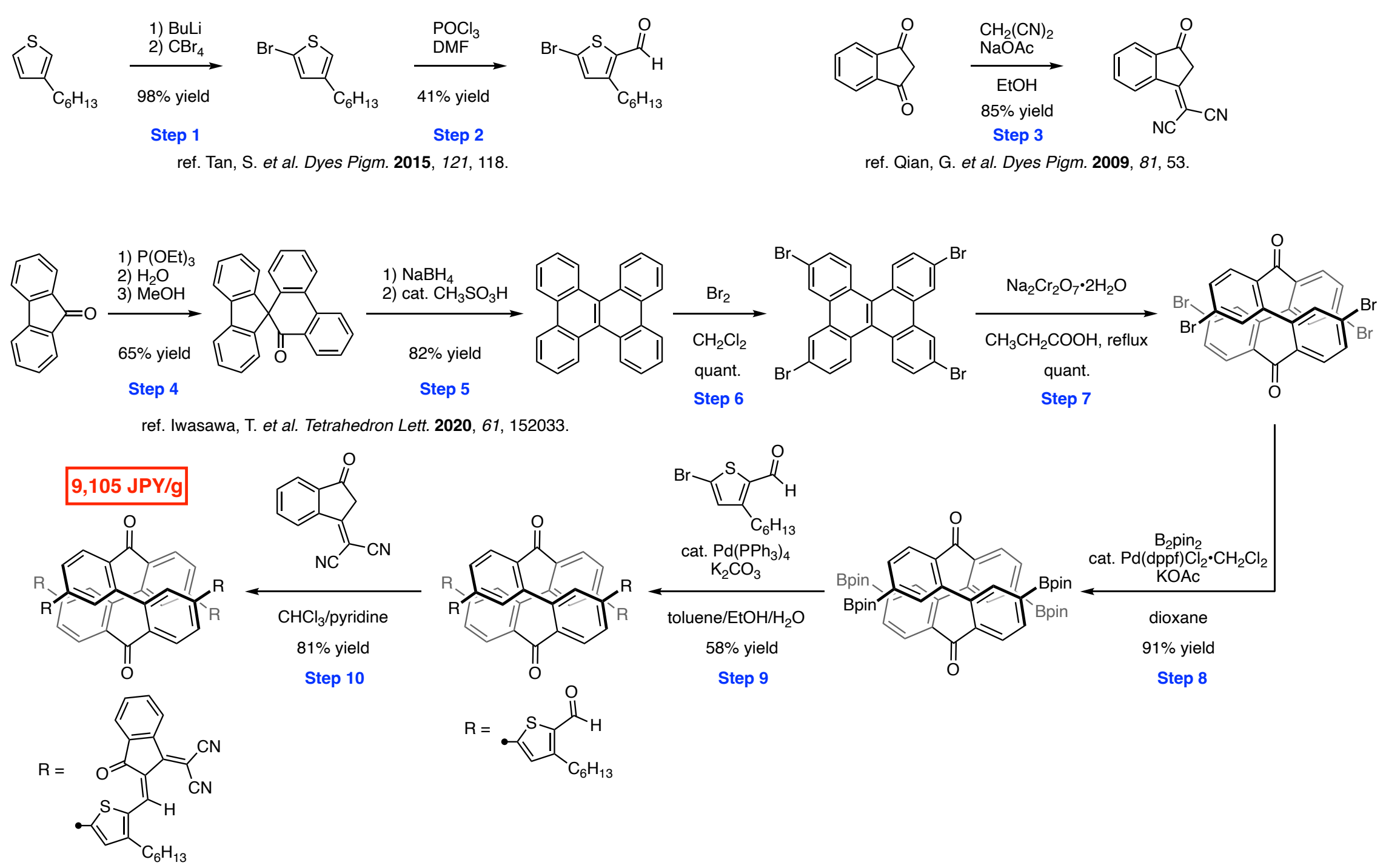

Figure S27. Synthetic route to CBBC-TIC **1a**.

Table S5. Detailed calculation of the synthesis cost of CBBC-TIC **1a**.

Step 1

Reagents	Used amounts	Cost	Price and amount	
3-hexylthiophene	8 g	6400 JPY	8000 JPY	10 g
THF	130 mL	1352 JPY	5200 JPY	500 mL
BuLi in hexane	23 mL	869.4 JPY	18900 JPY	500 mL
CBr_4	18.92 g	662.2 JPY	17500 JPY	500 mL
		9283.6 JPY		

Chemical yield	0.98	
Obtained product	46.58	mmol
Price of product	199.29	JPY/mmol

Step 2

Reagents	Used amounts	Cost	Price and amount	
bromohexylthiophene*	11.5 g	6654.36411 JPY	199.29 JPY	0.34441 g
DCE	100 mL	324 JPY	1620 JPY	500 mL
DMF	4.3 mL	24.08 JPY	2800 JPY	500 mL
$POCl_3$	5.2 mL	68.0536913 JPY	3900 JPY	298 mL
*Synthesized		7070.4978 JPY		

Chemical yield	0.41	
Obtained product	13.69	mmol
Price of product	516.47	JPY/mmol

Step 3

Reagents	Used amounts	Cost	Price and amount	
1,3-indandione	4.4 g	2904 JPY	16500 JPY	25 g
malononitrile	4 g	117.6 JPY	14700 JPY	500 g
EtOH	30 mL	130 JPY	13000 JPY	3000 mL
NaOAc	3.2 g	14.848 JPY	2320 JPY	500 g
		3166.448 JPY		

Chemical yield	0.85	
Obtained product	25.59	mmol
Price of product	123.74	JPY/mmol

Step 4

Reagents	Used amounts	Cost	Price and amount	
fluorenone	80 g	2304 JPY	14400 JPY	500 g
$P(OEt)_3$	153 mL	2999 JPY	9800 JPY	500 mL
water	160 mL	16 JPY	100 JPY	1000 mL
MeOH	900 mL	1215 JPY	4050 JPY	3000 mL
		6534 JPY		

Chemical yield	0.65	
Obtained product	144.28	mmol
Price of product	45.29	JPY/mmol

Step 5

Reagents	Used amounts	Cost	Price and amount	
spiroketone*	30 g	3945 JPY	45 JPY	0.34441 g
$NaBH_4$	1.32 g	41 JPY	15600 JPY	500 g
toluene	132 mL	213 JPY	4830 JPY	3000 mL
MeOH	24 mL	32 JPY	4050 JPY	3000 mL
acetone	0.64 mL	1 JPY	5100 JPY	3000 mL
water	300 mL	30 JPY	100 JPY	1000 mL
CH_3SO_3H	0.06 mL	2 JPY	13400 JPY	500 mL
*Synthesized		4264 JPY		

Chemical yield	0.82	
Obtained product	71.43	mmol
Price of product	59.69	JPY/mmol

Step 6

Reagents	Used amounts	Cost	Price and amount	
DBC*	16.4 g	2981 JPY	60 JPY	0.32841 g
Br_2	50 g	746 JPY	7460 JPY	500 g
I_2	1.3 mL	23 JPY	8810 JPY	500 g
CH_2Cl_2	2000 mL	889 JPY	8000 JPY	18000 mL
*Synthesized		4639 JPY		

Chemical yield	1.00	
Obtained product	49.94	mmol
Price of product	92.89	JPY/mmol

Step 7

Reagents	Used amounts	Cost	Price and amount	
DBC-4Br*	6.4 g	923 JPY	93 JPY	0.644 g
$Na_2Cr_2O_7{\cdot}2H_2O$	29.8 g	484 JPY	8120 JPY	500 g
CH_3CH_2COOH	500 mL	2050 JPY	2050 JPY	500 mL
*Synthesized		3457 JPY		

Chemical yield	0.96	
Obtained product	9.54	mmol
Price of product	362.36	JPY/mmol

Step 8

Reagents	Used amounts	Cost	Price and amount	
CBBC-4Br*	3.36 g	1812 JPY	362 JPY	0.672 g
B_2pin_2	6.1 g	3953 JPY	64800 JPY	100 g
$Pd(dppf)Cl_2{\cdot}CH_2Cl_2$	0.49 g	1931 JPY	98500 JPY	25 g
KOAc	2.94 g	23 JPY	3900 JPY	500 g
dioxane	100 mL	254 JPY	7630 JPY	3000 mL
*Synthesized		7972 JPY		

Chemical yield	0.91	
Obtained product	4.55	mmol
Price of product	1752.19	JPY/mmol

Step 9

Reagents	Used amounts	Cost	Price and amount	
CBBC-4Bpin*	0.184 g	373 JPY	1752 JPY	0.864 g
bromoformylhexylthiophene*	0.469 g	880 JPY	516 JPY	0.2752 g
$Pd(PPh_4)$	0.073 g	231 JPY	79200 JPY	25 g
toluene	4.2 mL	7 JPY	4830 JPY	3000 mL
EtOH	2.1 mL	9 JPY	13000 JPY	3000 mL
H_2O	2.1 mL	0 JPY	100 JPY	1000 mL
*Synthesized		1501 JPY		

Chemical yield	0.58	
Obtained product	0.12	mmol
Price of product	12149.29	JPY/mmol

Step 10

Reagents	Used amounts	Cost	Price and amount	
precursor*	0.068 g	727 JPY	12149 JPY	1.136 g
dicyano indandione*	0.093 g	59 JPY	124 JPY	0.1942 g
pyridine	0.21 mL	1 JPY	16600 JPY	3000 mL
$CHCl_3$	9 mL	25 JPY	8400 JPY	3000 mL
*Synthesized		813 JPY		

Chemical yield	0.81	
Obtained product	0.05	mmol
Price of product	16764.95	JPY/mmol
=	9106.44	JPY/g

10. Electron transport simulations

Method. Amorphous aggregate structures of enantiopure CBBC-TIC **1a**, racemic CBBC-TIC **1a**, and BP-TIC **8**, each comprising 4000 molecules in a cubic periodic cell, were constructed via molecular dynamics (MD) simulations under the NVT ensemble at 573 K for 0.1 ns, and then under the NPT ensemble at 300 K and 1.0×10^{-9} bar for 2.0 ns., using Dreiding force field with atomic point charges determined via the Merz–Singh–Kollman scheme at geometries optimized at the B3LYP/6-31G(d) level of theory. The resulting density was 1.0 g cm^{-3} for all three aggregates. A CBBC-TIC **1a** molecule can be viewed as a dimer of BP-TIC **8** molecules. In what follows, the two BP-TIC **8** fragments comprising each CBBC-TIC **1a** molecule were treated as separate BP-TIC **8** molecules. Reorganization energies for electron transfer were determined with quantum mechanics/molecular mechanics (QM/MM) calculations for randomly sampled 15 (for enantiopure CBBC-TIC **1a**), 14 (for racemic CBBC-TIC **1a**), and 20 (for BP-TIC **8**) molecules from the aggregate structures. The QM and MM regions were treated at the ωB97XD/Def2SVP level and the Universal Force Field, respectively, where the MM region comprised molecules within 30 Å of the QM region and its geometry was fixed. Electrostatic embedding was not applied. The resulting average reorganization energies were 318.5±20.2 meV (for enantiopure CBBC-TIC **1a**), 353.3±15.0 meV (for racemic CBBC-TIC **1a**), and 305.7±19.6 meV (for BP-TIC **8**), where the uncertainties indicate the standard errors. Calculations of electronic couplings between neighboring molecular pairs, rate constants for intermolecular electron hopping, and the kinetic Monte Carlo (kMC) simulations were performed as previously reported.[69,70] Intermolecular electron transfer was assumed to occur between the LUMOs of each molecule, and the contribution of higher orbitals (e.g. LUMO+1) was neglected for simplicity. MD simulations and quantum chemical calculations were conducted using LAMMPS and Gaussian 16 program packages, respectively, and the kMC simulations were performed using an in-house program.

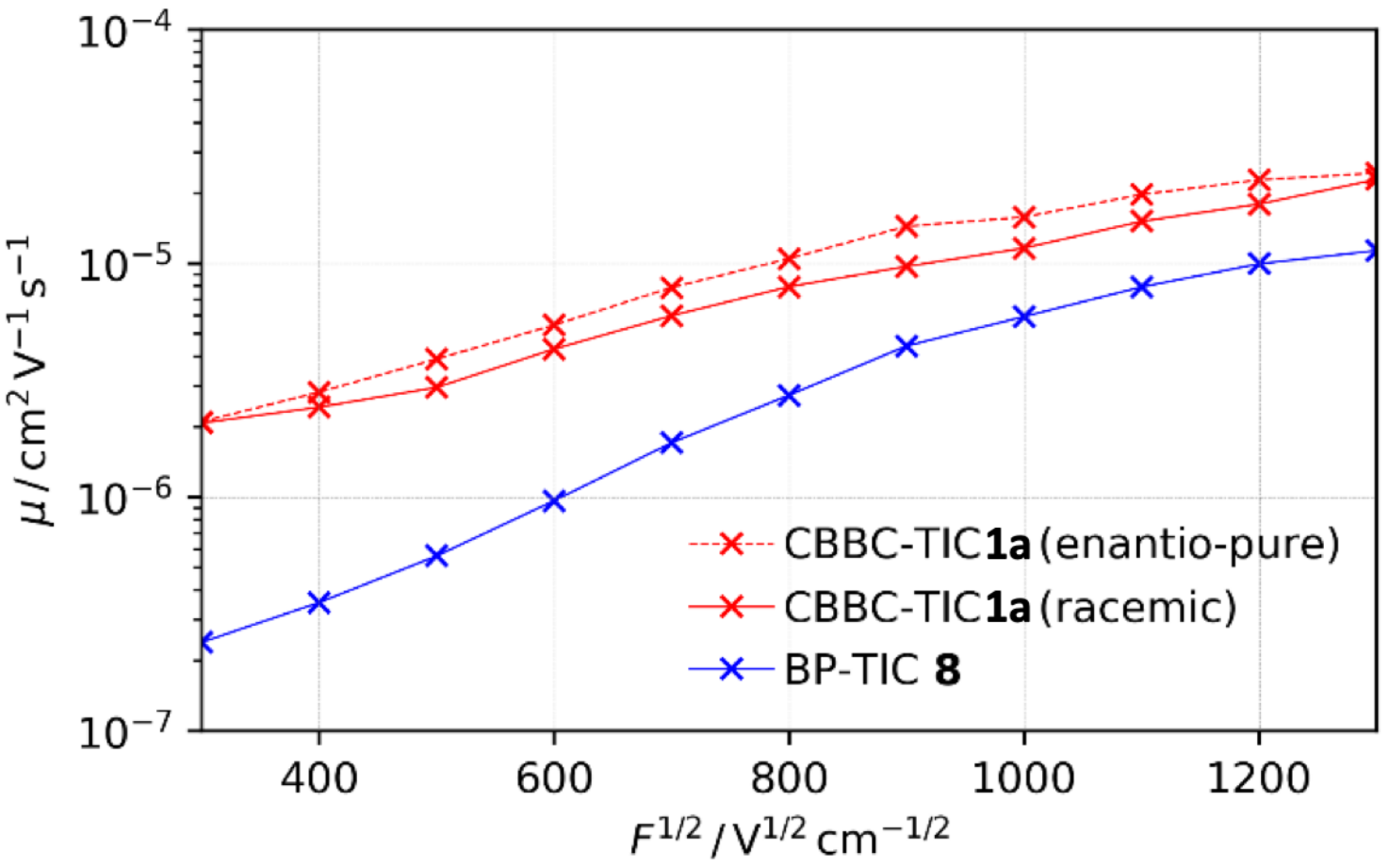

Figure S28. Simulated electron mobilities (μ_e) of BP-TIC **8** (blue), racemic CBBC-TIC **1a** (red, solid), and enantiopure CBBC-TIC **1a** (red, dashed), as a function of the square root of the applied electric field ($F^{1/2}$).

11. Fabrication of perovskite solar cells

Materials for perovskite solar cell fabrication.

Cesium iodide (CsI, >99%), methylammonium bromide (MABr, >98%, low water content), lead(II)iodide (PbI_2, 99.99%, trace metals basis), lead(II) bromide ($PbBr_2$, >98%, for perovskite precursor), formamidinium iodide (FAI, >98%, low water content), bathocuproine (BCP, 99%, purified by sublimation), and [[5H-Diindolo[3,2-a:3',2'-c]carbazole-5,10,15-triyl]tris(propane-3,1-diyl)]tris(phosphonic Acid) (3PATAT-C3, >98%) were purchased from Tokyo Chemical Industry Co., Ltd. (TCI). Ethylenediamine dihydroiodide ($EDAI_2$, >98%), and alminum oxide (Al_2O_3) nanoparticles (dispersed in IPA, 20 wt%, <50 nm particle size) were purchased from Sigma-Aldrich Co., Ltd. (Sigma-Aldrich). [6,6]-phenyl-C_{61}-butyric acid methyl ester ($PC_{61}BM$, 99.5%) was purchased from Frontier Carbon Corporation. Dimethylsulfoxide (DMSO, super dehydrated) and isopropyl alcohol (IPA, super dehydrated) was purchased from FUJIFILM Wako Pure Chemical Co., Ltd. Dimethylformamide (DMF), chlorobenzene, and toluene were purchased from Kanto Chemical Co., Inc. All of these solvents were degassed by argon (Ar) gas bubbling for 1 h and further dried over molecular sieves (3 Å) in an Ar-filled glove box (H_2O, O_2 <1 ppm) before use.

Device fabrication.

Glass/indium tin oxide (ITO, 10 Ω sq^{-1}, Geomatec Co., Ltd.) substrates (2.5 cm × 2.5 cm) were etched with zinc powder and 6 M hydrochloride (HCl) aqueous solution. They were consecutively cleaned in an ultrasonic bath for 15 min each with acetone, water, detergent solution (Semico Clean 56, Furuuchi chemical), water, and IPA. After drying with hot air, the substrates were cleaned by plasma treatment to remove organic residuals. The substrates were transferred to a nitrogen-filled glovebox. A 0.1 mM solution of 3PATAT-C3 in DMF was filtered through a 0.45 μm PTFE filter and spun onto the plasma-treated ITO surface using the following program: slope 5 s, 3000 rpm for 30 s, slope 5 s. The films were subsequently annealed on a hot plate at 110 °C for 10 min. After cooling to room temperature, a dispersion of Al_2O_3 nanoparticles in IPA (4 mg mL^{-1}) was spun using the following program: slope 5 s, 4000 rpm for 30 s, slope 5 s. The films were subsequently annealed on a hot plate at 100 °C for 15 min.

A 1.3 M precursor solution of $Cs_{0.05}FA_{0.80}MA_{0.15}PbI_{2.75}Br_{0.25}$ was prepared by dissolving CsI (51 mg, 0.20 mmol), MABr (66 mg, 0.59 mmol), PbI_2 (1.708 g, 3.71 mmol), $PbBr_2$ (72 mg, 0.20 mmol), and FAI (536 mg, 3.12 mmol) in a mixed solvent of DMF (2.31 mL) and DMSO (0.69

mL). After stirring at 40 °C overnight, the solution was filtered through a 0.45 μm PTFE filter. To fabricate thin film, 190 μL of the solution was spin-coated onto a substrate using the following program: slope 1 s, 1000 rpm for 10 s, slope 5 s, 6000 rpm for 20 s, slope 5 s. Chlorobenzene (300 μL) was dripped onto the rotating substrate 4 s prior to the end of the spinning step at 6000 rpm. The films were subsequently annealed on a hot plate at 150 °C for 10 min.

For the $EDAI_2$ surface treatment, $EDAI_2$ was dissolved in IPA/toluene (1:1) at a concentration of 0.5 mg mL^{-1} and stirred at 70 °C overnight. After cooling to room temperature, the solution was filtered through a 0.45 μm PTFE filter. 100 μL of the solution was spin-coated onto the perovskite films using the following program: slope 3 s, 4000 rpm for 20 s, slope 3 s. The films were subsequently annealed on a hot plate at 100 °C for 10 min.

To prepare the electron transport layer, CBBC-TIC **1a** or BP-TIC **8** was dissolved in tetrachloroethane (TCE) under reflux at a concentration of 15 mg mL^{-1}. PCBM was dissolved in chlorobenzene at 40 °C at a concentration of 25 mg mL^{-1}. After cooling to room temperature, the solutions were filtered through a 0.45 μm PTFE filter. 90 μL of the solution was spin-coated onto the perovskite films using the following program: slope 1 s, 1000 rpm for 30 s, slope 1 s. The films were subsequently annealed on a hot plate at 100 °C for 10 min.

8 nm of bathocuproine (BCP) was deposited by thermal evaporation at a rate of 0.1 Å s^{-1}. Finally, a 100 nm of silver (Ag) metal electrode was deposited through a shadow mask. The deposition rate was initially set as 0.03 Å s^{-1} until 2 nm, increased to 0.1 Å s^{-1} until 10 nm, 0.2 Å s^{-1} until 20 nm, and finally 0.9 Å s^{-1} until 100 nm. The overlap area between the top Ag electrode and the bottom ITO was approximately 0.15 cm^2.

Characterization.

Current density–voltage (*J*–*V*) curves were measured in a nitrogen-filled glovebox (H_2O, O_2 < 1 ppm) with an OTENTO-SUN-P1G solar simulator (BUNKOUKEIKI Co., Ltd.) and a Keithley 2400 SourceMeter. The light intensity of the illumination source was calibrated using a standard silicon photodiode. External quantum efficiency (EQE) was measured by a SMO-250III system equipped with an SM-250 diffuse reflection unit (BUNKOUKEIKI Co., Ltd.). The light intensity of the illumination source was calibrated with a standard SiPD S1337-1010BQ silicon photodiode. The active area of the devices was 0.0985 cm^2 as defined by the aperture of the shadow mask placed between the light source and test cells.

Impedance spectroscopy data was obtained with an E4990A impedance analyser (Keysight) with an oscillator voltage of 30 mV, in the frequency range of 20-200,000 Hz. The measurements

were performed with the devices exposed to AM1.5G-equivalent radiation in an inert atmosphere, with a 0.1 cm^2 shadow mask. The impedance data was fit by a series resistor (r_s) along with a capacitor (c_p) in parallel with a resistor (r_p).

Two-dimensional grazing-incidence X-ray diffraction (2D-GIXD) measurements were performed on Rigaku SmartLab equipped with a hybrid pixel array detector HyPix-3000.

Scanning electron microscopy (SEM) was performed with a SU-8600 (Hitachi High-Technologies Co.) instrument.

Atomic force microscopy (AFM) was performed with a Picoscan Plus AFM instrument used in AC-mode with Nanoworld NCST probes.

Ellipsometry measurement was conducted by Stokes ellipsometer LSE-100 (Gaertner Scientific).

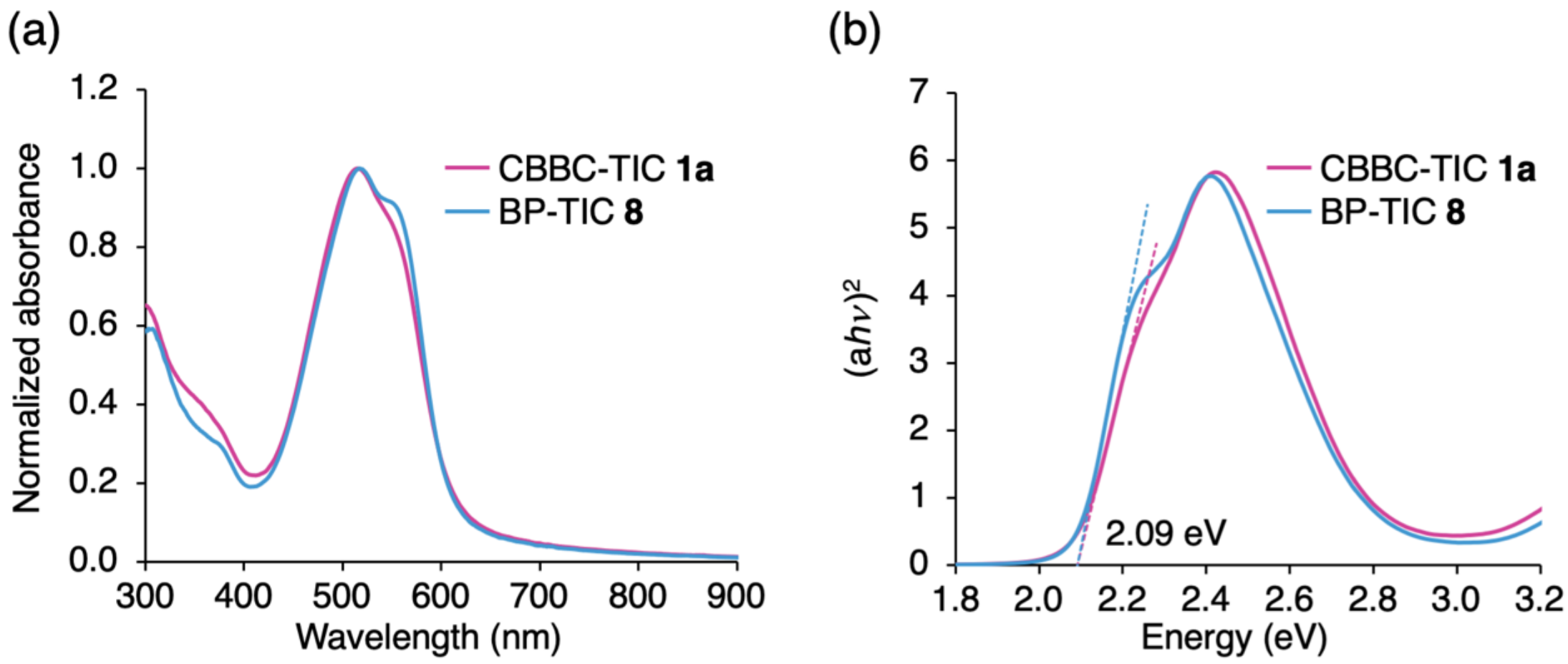

Figure S29. (a) Absorption spectra and (b) Tauc plots of the spin-coated films of CBBC-TIC **1a** and BP-TIC **8**.

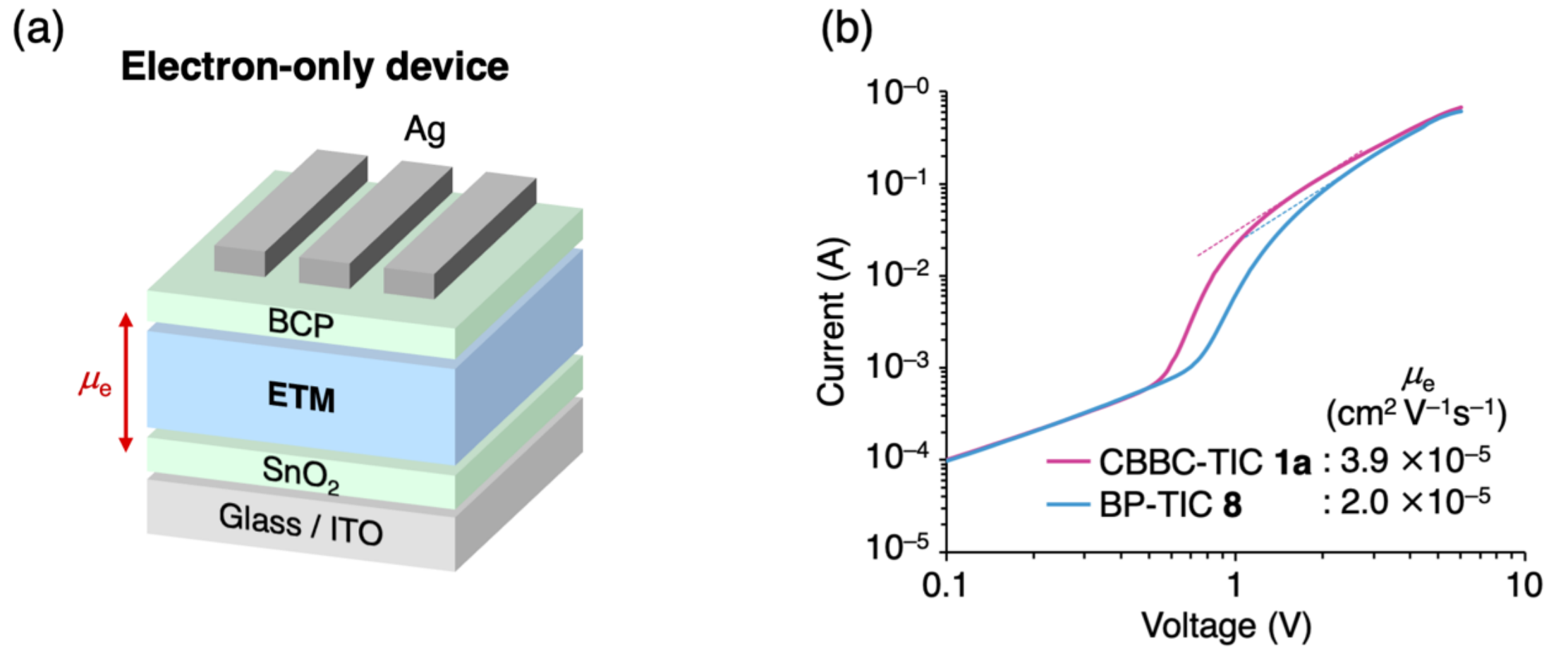

Figure S30. (a) Structure of electron-only device and (b) space-charge-limited current (SCLC) of CBBC-TIC **1a** and BP-TIC **8**.

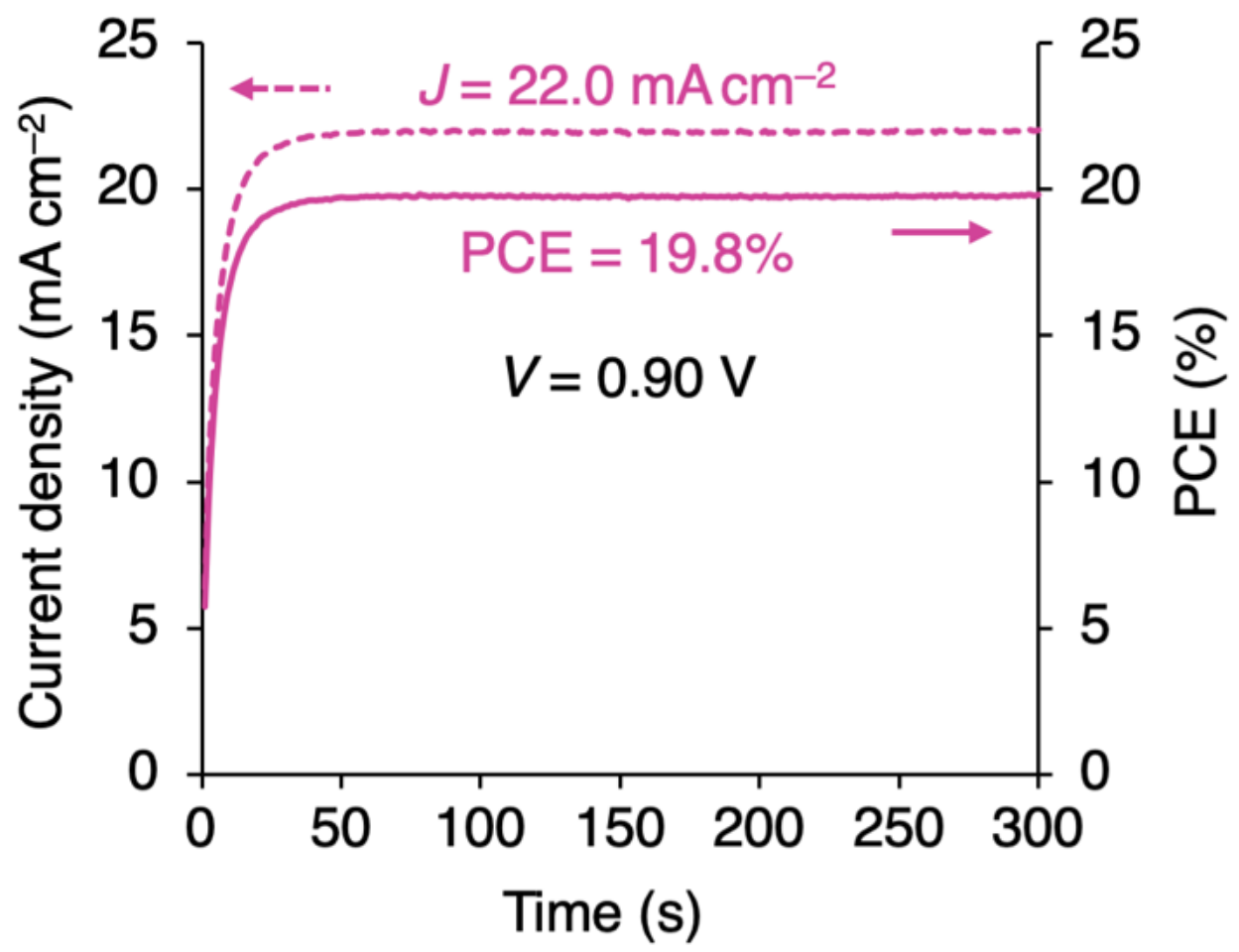

Figure S31. (a) Stabilized current density (dashed line) and stabilized power output (solid line) of a representative perovskite solar cell using CBBC-TIC **1a**, measured at a bias of 0.90 V under AM 1.5G, 100 mW cm^{-2} irradiation.

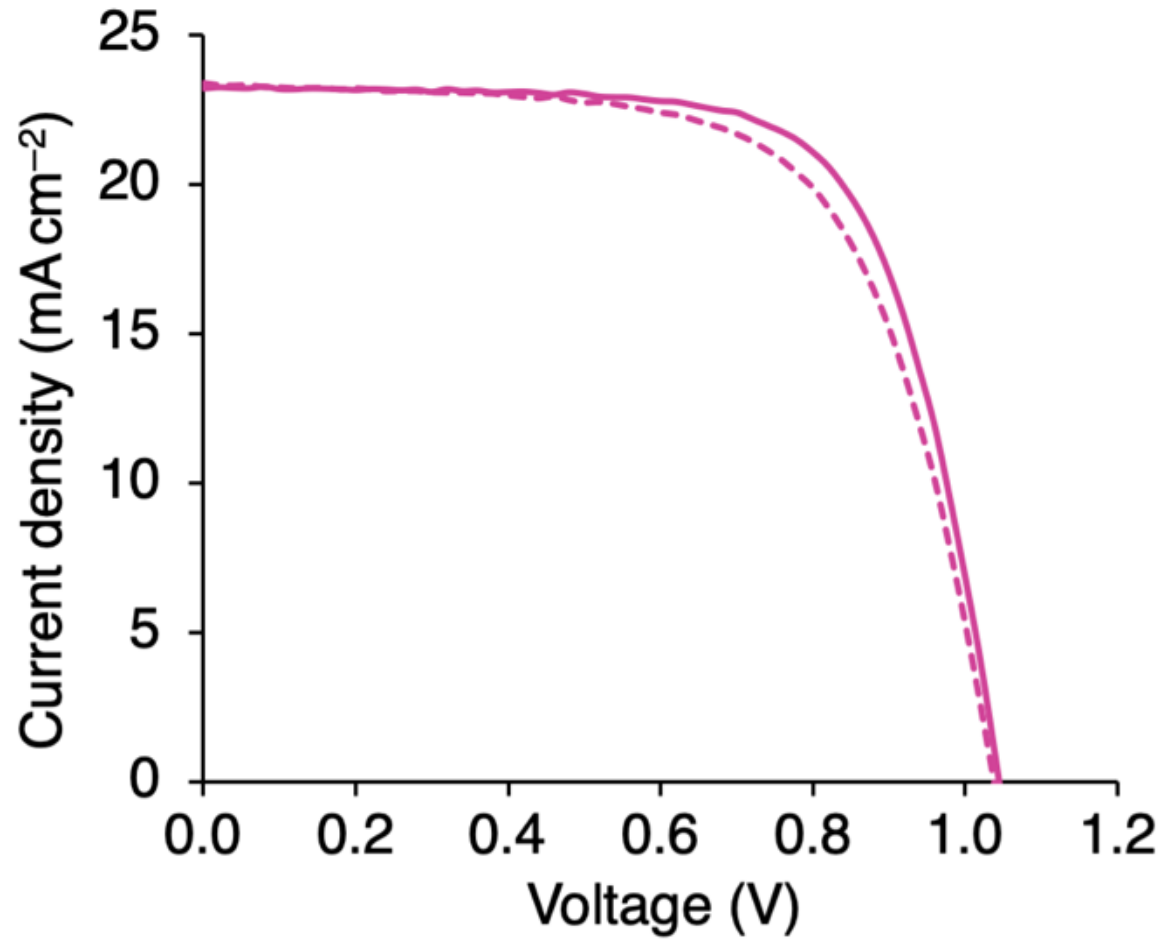

Figure S32. Current–voltage (*J*–*V*) curves of forward (J_{SC} to V_{OC}, dotted line) and reverse (V_{OC} to J_{SC}, solid line) scans for the solar cell using enantiopure CBBC-TIC, (*P*,*P*)-**1a** as the ETM.

Table S6. Photovoltaic parameters of PSCs using enantiopure CBBC-TIC, (*P*,*P*)-**1a** as the ETM.

Scan	J_{SC} (mA cm^{-2})	V_{OC} (V)	FF	PCE (%)
Forward	23.4<br>(23.2±0.2)	1.04<br>(1.02±0.01)	0.66<br>(0.61±0.02)	15.9<br>(14.5±0.8)
Reverse	23.2<br>(23.0±0.2)	1.04<br>(1.03±0.01)	0.70<br>(0.65±0.02)	16.9<br>(15.5±0.8)

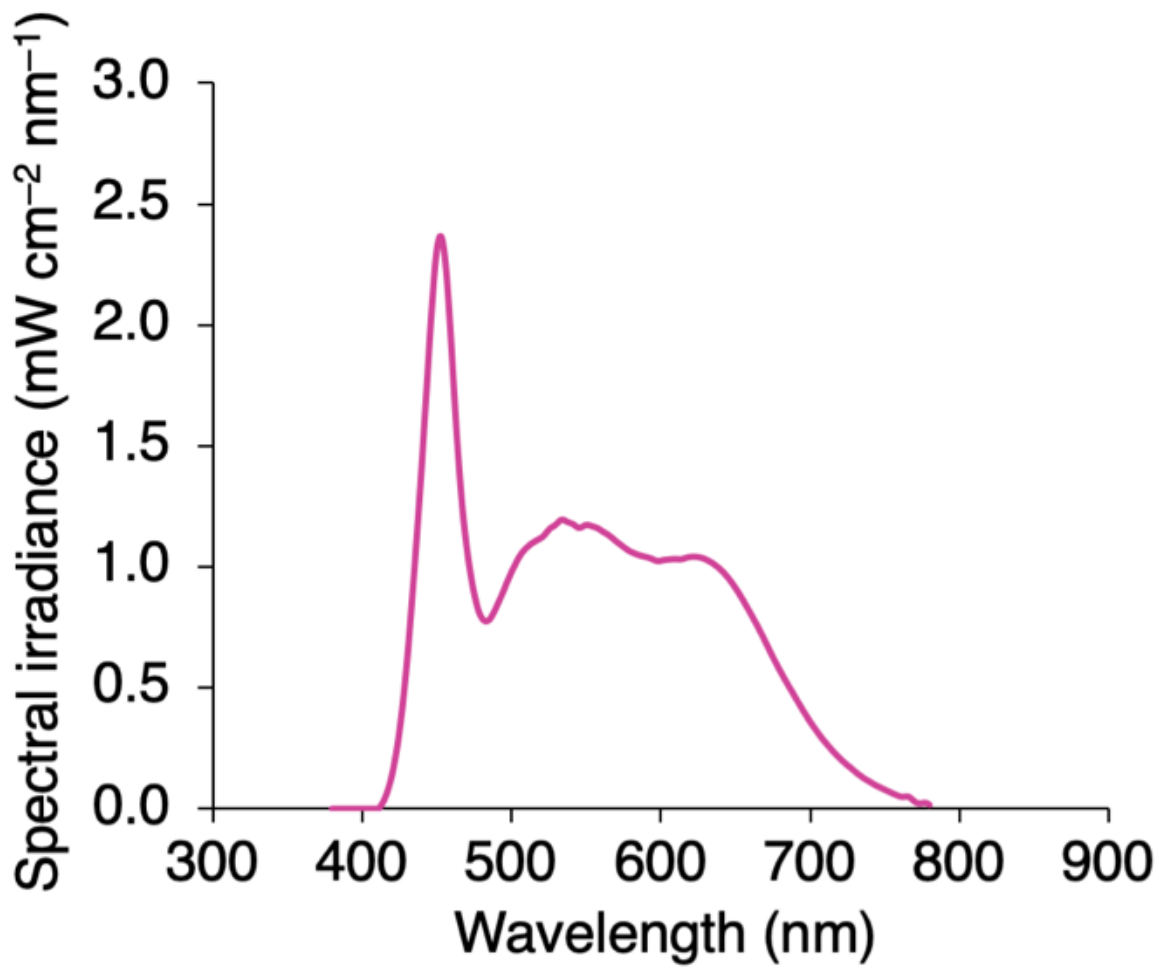

Figure S33**.** Spectrum of 800 lux white LED.

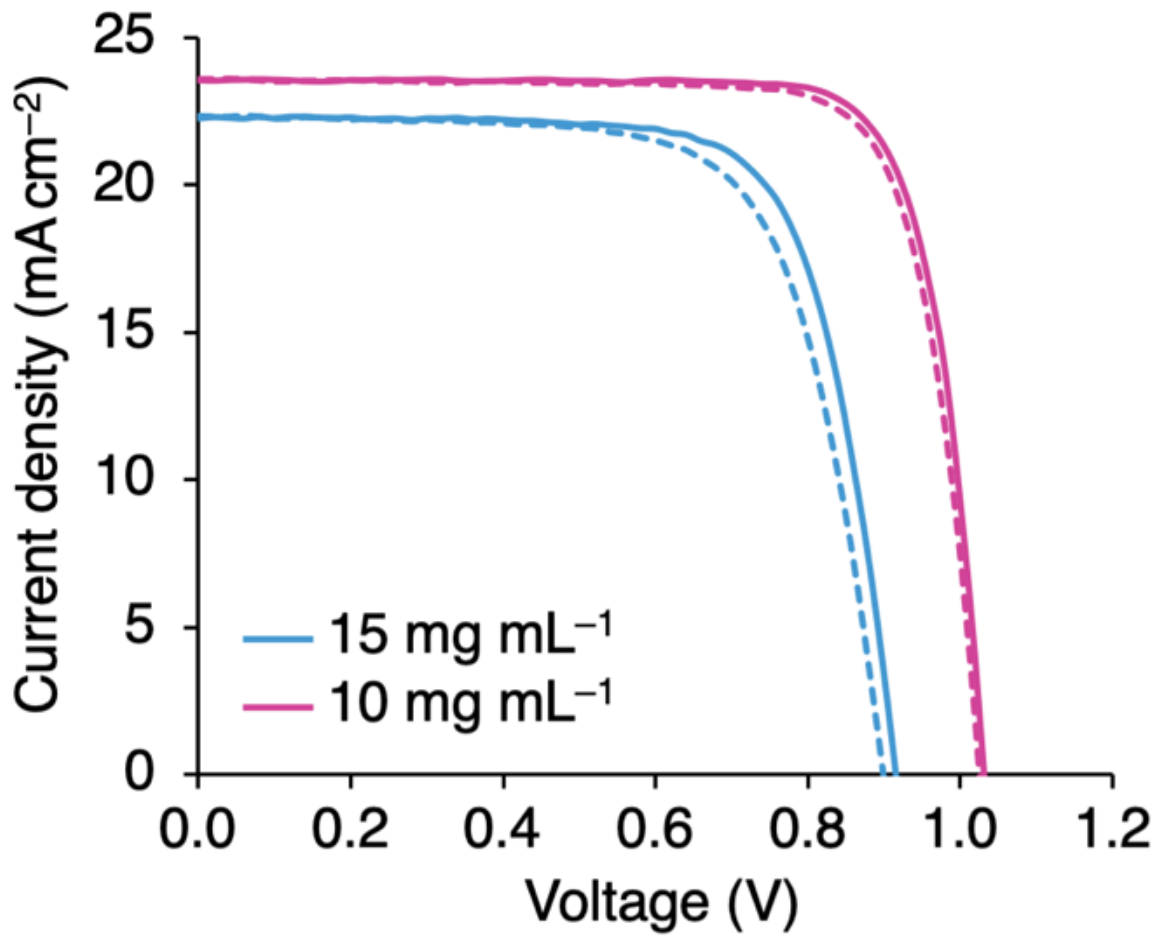

Figure S34. Current–voltage (*J*–*V*) curves of forward (J_{SC} to V_{OC}, dotted lines) and reverse (V_{OC} to J_{SC}, solid lines) scans for the solar cell using tetramethyl CBBC-TIC **1b** as the ETM at different solution concentrations.

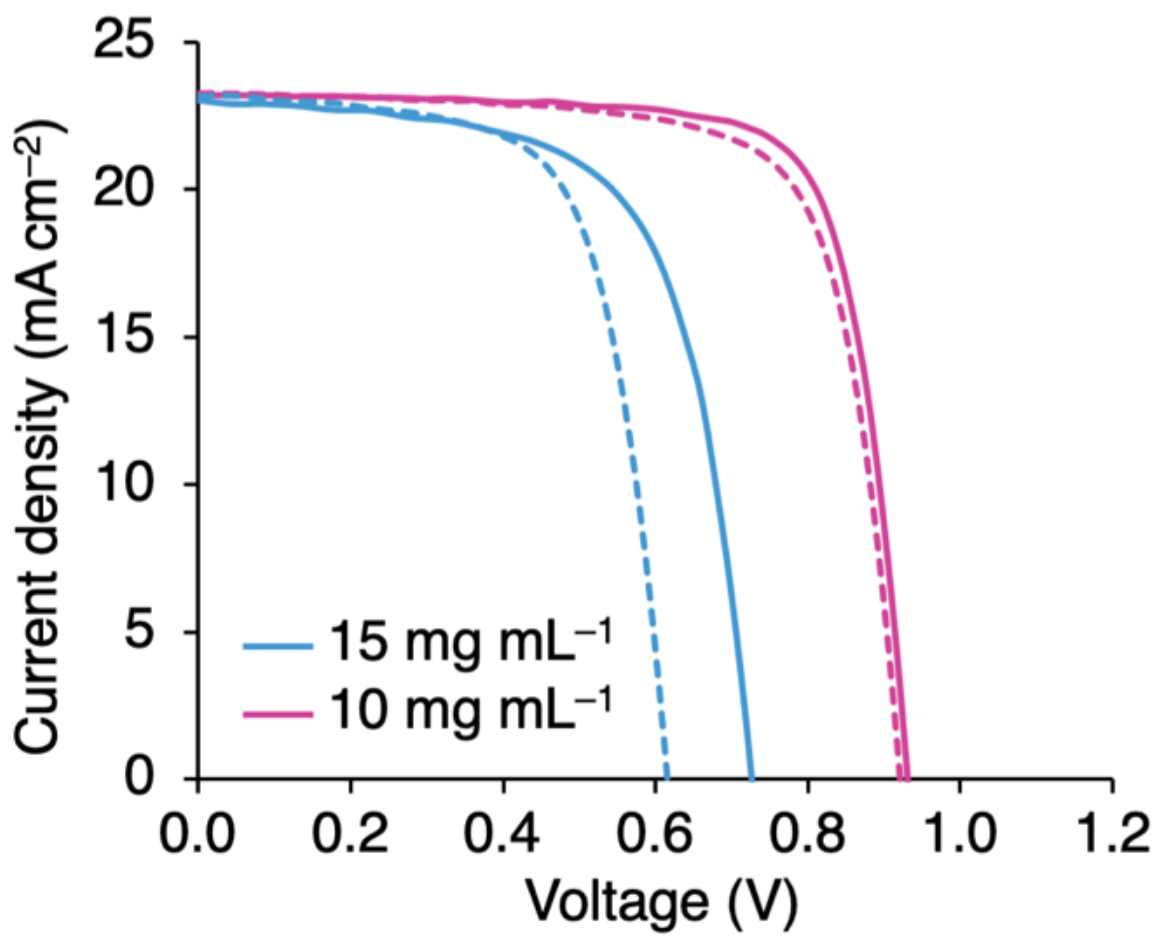

Figure S35. Current–voltage (*J*–*V*) curves of forward (J_{SC} to V_{OC}, dotted line) and reverse (V_{OC} to J_{SC}, solid line) scans for the solar cell using octamethyl CBBC-TIC **1c** as the ETM at different solution concentrations.

12. Magnetic conductive-AFM measurement

CISS measurements were performed using the same methodology as described in our previous study.[40] Samples were fabricated by spin-coating 30 μL of a 3.0 mg/mL 1,1,2,2-tetrachloroethane solution of (*M*,*M*)-**1a** or (*P*,*P*)-**1a** onto highly oriented pyrolytic graphite (HOPG) at 1000 rpm followed by thermal annealing at 70 °C for 1 h. The film thicknesses on HOPG were determined using the same AFM instrument operated in tapping mode with a silicon cantilever tip (Bruker OLTESPA-R3), by measuring the step height of partially scratched films. The film thickness was measured at five or more different positions, and the average value was used. The thicknesses of the (*M*,*M*)-**1a** and (*P*,*P*)-**1a** films were 23.85 ± 5.42 nm and 19.81 ± 5.35 nm, respectively. Spin-polarized conductive-AFM measurements were performed on a Bruker Innova atomic force microscope equipped with a nonmagnetic sample holder (a Bruker APSH-0020) and a CoCr-cantilever (a Bruker MESP-V2). A disc-shaped Nd magnet was put with up or down magnetization directions on the CoCr-cantilever at a distance of ca. 6 mm and the *I*-*V* curves were recorded in contact with the surface. We measured more than 100 *I*-*V* curves at different locations for each sample. HOPG substrates were purchased from Bruker (ZYB grade) and freshly cleaved just before use. The cantilever was replaced when changing thin films to keep using a fresh cantilever. The mean magnetic conductance asymmetry (EMA) values were determined by averaging the EMA values in the voltage ranges of –5 to –1 V and +2 or +1 to +5 V.

The original obtained data for (*P*,*P*)-**1a** afforded an EMA value of–56.3 ± 2.2%. This EMA value was largely deviated from that of (*M*,*M*)-**1a** (+80.1 ± 7.5%). To address this issue, we systematically reanalyzed the dataset and found that the original *I*–*V* data for the (*P*,*P*)-enantiomer included a subset of unusually low-intensity curves. We attribute these anomalously low-current curves to transient contact failure between the conductive tip and the organic film during the automated data acquisition process. After carefully inspecting the dataset, these low-current artifacts were excluded to ensure data integrity. Even after this exclusion, a statistically robust set of 236 independent *I*-*V* curves remained in the dataset, which is fully sufficient to provide reliable averaged *I*-*V* curves and the resulting EMA values. After excluding these non-physical artifacts from the analysis, the recalculated EMA value improved to −76.6 ± 3.4%.

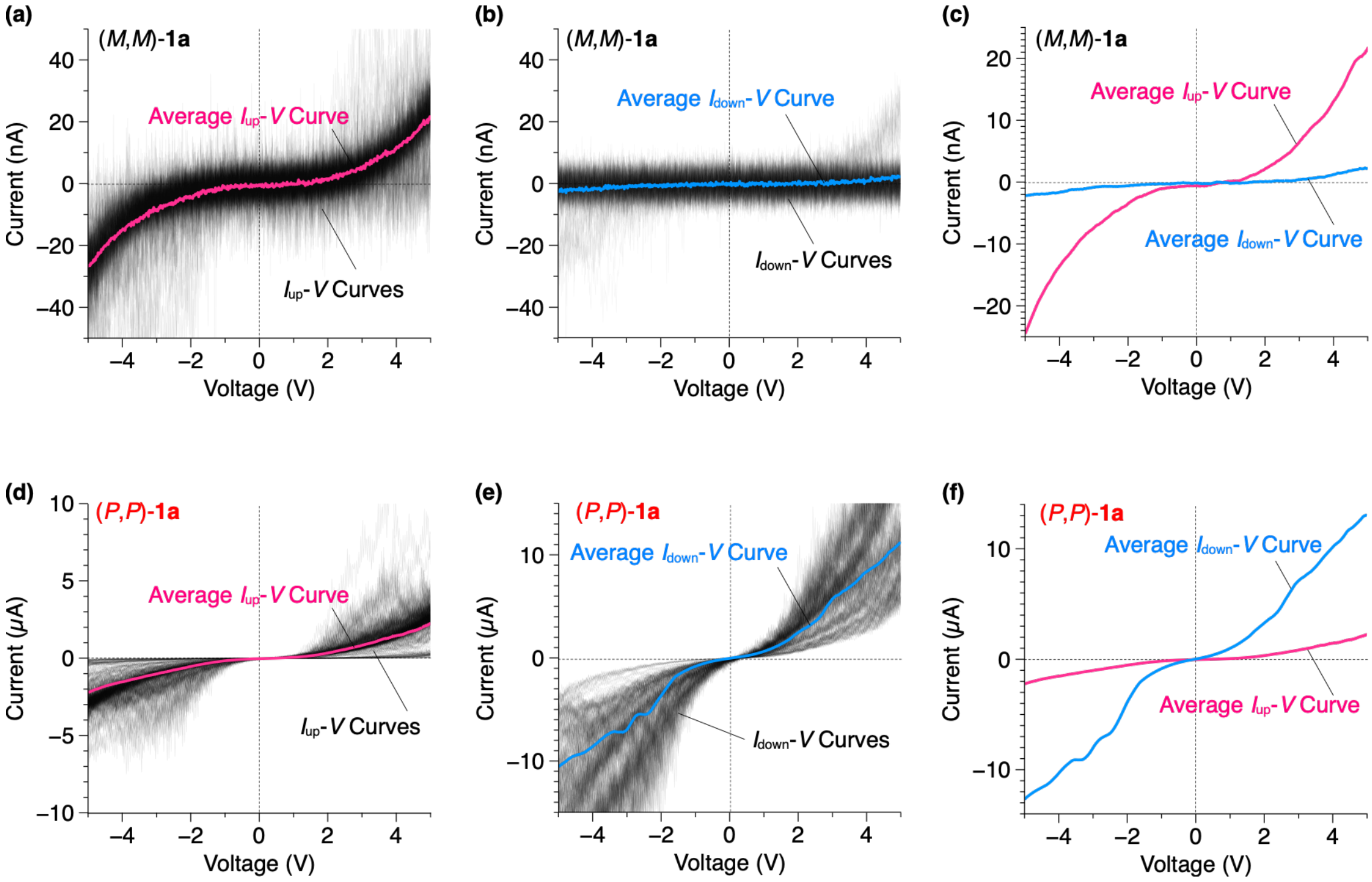

Figure S36. (a) Unaveraged (black curves) and averaged I_{up}-V (pink curve) curves of (*M*,*M*)-**1a**. (b) Unaveraged (black curves) and averaged I_{down}-V (sky blue curve) curves of (*M*,*M*)-**1a**. (c) Averaged I_{up}-V and I_{down}-V curves of (*M*,*M*)-**1a**. (d) Unaveraged (black curves) and averaged I_{up}-V (pink curve) curves of (*P*,*P*)-**1a**. (e) Unaveraged (black curves) and averaged I_{down}-V (sky blue curve) curves of (*P*,*P*)-**1a**. (f) Averaged I_{up}-V and I_{down}-V curves of (*P*,*P*)-**1a**.

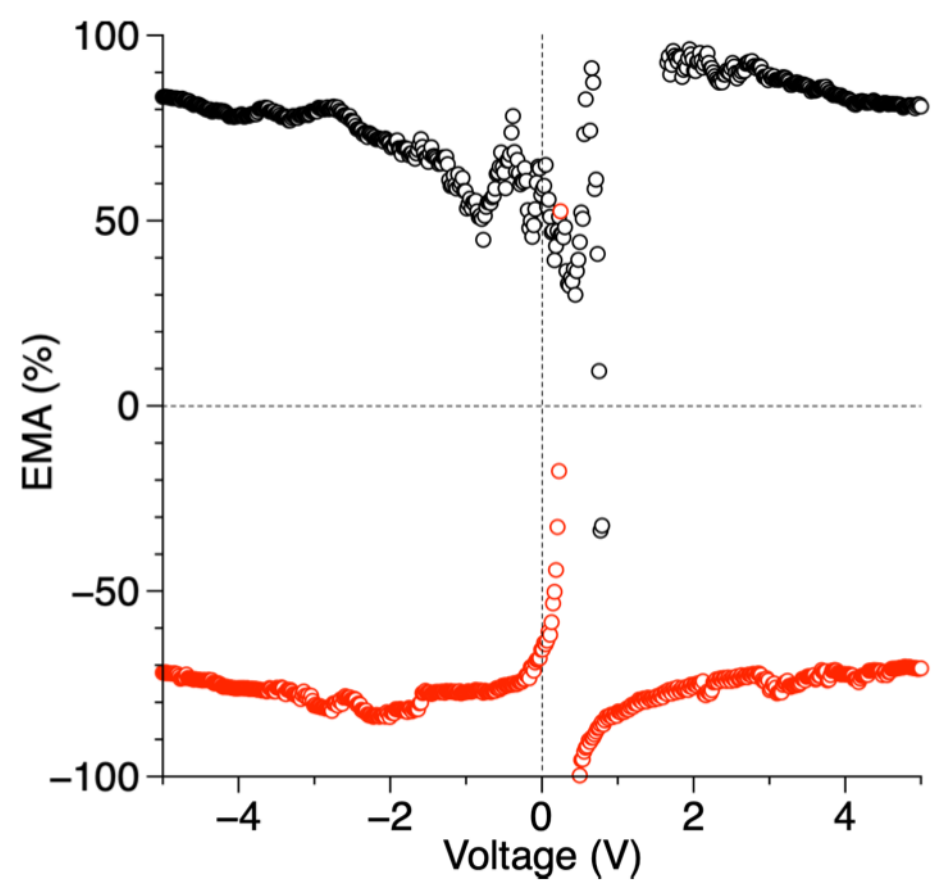

Figure S37. Enantiospecific magnetic conductance asymmetry (EMA) of (*M*,*M*)-**1a** (black circles) and (*P*,*P*)-**1a** (red circles).

13. References

[74] X. Liu, J. Long, G. Wang, Y. Pei, B. Zhao, S. Tan, "Effect of Structural Modification on the Performances of Phenothiazine-Dye Sensitized Solar Cells", *Dyes Pigm.* **2015,** *121,* 118.

[75] (a) Y. Cui, H. Ren, J. Yu, Z. Wang, G. Qian, "An Indanone-Based Alkoxysilane Dye with Second Order Nonlinear Optical Properties", *Dyes Pigm.* **2009,** *81,* 53. (b) S. Li, L. Ye, W. Zhao, S. Zhang, S. Mukherjee, H. Ade, J. Hou, "Energy-Level Modulation of Small-Molecule Electron Acceptors to Achieve over 12% Efficiency in Polymer Solar Cells", *Adv. Mater.* **2016**, *28*, 9423.

[76] X. Miao, Z. Cai, J. Li, L. Liu, J. Wu, B. Li, L. Ying, F. Silly, W. Deng, Y. Cao, "Elucidating Halogen-Assisted Self-Assembly Enhanced Mechanochromic Aggregation-Induced Emission", *ChemPhotoChem* **2021,** *5,* 626.

[77] M. J. Frisch, G. W. Trucks, H. B. Schlegel, G. E. Scuseria, M. A. Robb, J. R. Cheeseman, G. Scalmani, V. Barone, G. A. Petersson, H. Nakatsuji, X. Li, M. Caricato, A. V. Marenich, J. Bloino, B. G. Janesko, R. Gomperts, B. Mennucci, H. P. Hratchian, J. V. Ortiz, A. F. Izmaylov, J. L. Sonnenberg, D. Williams-Young, F. Ding, F. Lipparini, F. Egidi, J. Goings, B. Peng, A. Petrone, T. Henderson, D. Ranasinghe, V. G. Zakrzewski, J. Gao, N. Rega, G. Zheng, W. Liang, M. Hada, M. Ehara, K. Toyota, R. Fukuda, J. Hasegawa, M. Ishida, T. Nakajima, Y. Honda, O. Kitao, H. Nakai, T. Vreven, K. Throssell, J. A. Montgomery Jr., J. E. Peralta, F. Ogliaro, M. J. Bearpark, J. J. Heyd, E. N. Brothers, K. N. Kudin, V. N. Staroverov, T. A. Keith, R. Kobayashi, J. Normand, K. Raghavachari, A. P. Rendell, J. C. Burant, S. S. Iyengar, J. Tomasi, M. Cossi, J. M. Millam, M. Klene, C. Adamo, R. Cammi, J. W. Ochterski, R. L. Martin, K. Morokuma, O. Farkas, J. B. Foresman, D. J. Fox, "Gaussian 16, Revision B.01", Gaussian, Inc., Wallingford, CT, 2016.

[78] (a) A. D. Becke, "Density-Functional Exchange-Energy Approximation with Correct Asymptotic Behavior", *Phys. Rev. A* **1988,** *38,* 3098; (b) C. Lee, W. Yang, R. G. Parr, "Development of the Colle–Salvetti Correlation-Energy Formula into a Functional of the Electron Density", *Phys. Rev. B* **1988,** *37,* 785.
